# A Brief Web-Based and Mobile Intervention of Intermittent Fasting With Meal Support for Weight Loss Among Adults With Overweight and Obesity in Japan: Pilot Randomized Controlled Trial

**DOI:** 10.2196/58930

**Published:** 2026-01-26

**Authors:** Takashi Noda, Tomonari Shimamoto, Kosuke Kiyohara, Mie Imanaka, Norihiro Nishioka, Yukiko Tateyama, Taku Iwami

**Affiliations:** 1 Department of Preventive Services School of Public Health, Graduate School of Medicine Kyoto University Kyoto Japan; 2 Department of Food Science Faculty of Home Economics Otsuma Women’s University Tokyo Japan; 3 Department of Health and Nutrition Faculty of Nursing and Nutrition The University of Shimane Izumo Japan

**Keywords:** intermittent fasting, eHealth, mHealth, mobile health, weight loss, diet, overweight, obesity, randomized controlled trial

## Abstract

**Background:**

Intermittent fasting emerges as a promising dietary approach against obesity, offering a cost-effective strategy for implementation via web-based platforms. We developed a Brief Online Intermittent Fasting Program (OIF), featuring a self-administered, weekly 1-day fasting regimen with replacement meals delivery, online guidance, and app messaging to support adherence.

**Objective:**

This pilot study aimed to assess the preliminary effectiveness, feasibility, and safety of the OIF on weight loss in adults with overweight and obesity in Japan. Secondary objectives were to assess its effects on body composition and metabolic markers.

**Methods:**

This 12-week, 1:1 randomized controlled trial recruited adults with overweight and obesity (BMI from 23 to <35) in 1 university, 1 hospital, and 2 company offices. Participants were randomized into 2 groups stratified by sex and age (<40 or ≥40 years). The intervention group received very low-calorie (407 kcal) meal replacements for weekly intermittent fasting, online guidance via Zoom (Zoom Video Communications, Inc), and app messages encouraging fasting and healthy lifestyles. The control group received app messages promoting healthy lifestyles only. Interventions were administered by a nonblinded researcher. The primary outcome was the change in body weight after 12 weeks, analyzed using intention-to-treat principles and adjusted for sex, age, and baseline weight. Secondary outcomes encompassed body composition, blood pressure, biomarkers (eg, hemoglobin A_1c_, triglycerides, and cholesterol), quality of life, physical activity, intervention adherence, and adverse events.

**Results:**

A total of 57 individuals were enrolled (28 in the intervention group and 29 in the control group). At 12 weeks, 25 participants in the intervention group and 27 participants in the control group completed follow-up. The baseline median weight was 75.8 (IQR 68.3-80.6) kg for the intervention group and 74.8 (IQR 69.8-81.8) kg for the control group. The mean weight change was –0.9 (SD 1.9) kg in the intervention group and +0.6 (SD 1.4) kg in the control group. The adjusted between-group difference in weight change was statistically significant at –1.6 (95% CI –2.5 to –0.8) kg. Fat mass change was not statistically significant (–0.1, 95% CI –1.3 to 1.4 kg), but muscle mass reduction was implied (–1.3, 95% CI –2.5 to –0.2 kg). Intervention adherence was 79% (22/28) in the intervention group. No serious adverse events were reported, and there were no significant changes in key biomarkers, such as hemoglobin A_1c_ or quality of life.

**Conclusions:**

The OIF demonstrated effectiveness in promoting modest weight loss among adults with overweight and obesity over 12 weeks, with high feasibility and safety indicated by low dropout rates and absence of serious adverse events. However, the observed reduction in muscle mass indicates a need for program refinement, such as incorporating exercise guidance, to optimize health outcomes.

**Trial Registration:**

UMIN-CTR UMIN000050437; https://tinyurl.com/4x5h2t2x

## Introduction

Obesity is a well-known public health challenge, which increases the risk of cardiovascular diseases and mortality [1]. According to the World Health Organization, at least 2.8 million people die each year as a result of being overweight or obese [2], with the obesity epidemic still escalating at an alarming rate [3]. Moreover, the recent COVID-19 pandemic has further deteriorated our lifestyles and made the development of remote obesity interventions, using the internet, apps, and other web-based technologies, an even more urgent issue [4-6]. Although many web-based programs have been developed, with some degree of effectiveness acknowledged [7,8], few have been successfully implemented in society to yield significant results. Also, criticism exists toward the previous clinical trial approaches, which have focused too much on internal validity [9], indicating a demand for the development of highly feasible online weight loss programs that are easily applied and disseminated into society.

Recently, intermittent fasting (IF) has emerged as a popular strategy for combating obesity [10]. IF is a simple dietary restriction regimen that incorporates periodic fasting (energy restriction of less than 600 kcal) into normal eating habits [11,12]. Recent systematic reviews and meta-analyses have demonstrated the effectiveness of IF for weight loss compared to ad libitum eating [13]. The mechanisms behind weight loss with IF are related to energy restriction, increased fat metabolism, and improved glucose metabolism [14], which are common to any dietary restriction. IF may also provide cardiovascular health benefits. For example, studies with women with obesity show that a weekly IF intervention over 2 months significantly reduced resting heart rate, blood glucose, insulin, and homocysteine levels. These effects are likely associated with reductions in adipokines, including leptin, IL-6, tumor necrosis factor-alpha, and insulin-like growth factor-1, suggesting that IF could mitigate cardiovascular disease risk [15,16]. Additional benefits reported in recent studies include decreased hunger, reduced inflammation, improved cellular repair, regulated hormone production, enhanced immune function, adjusted circadian rhythms, and a more diverse gut microbiome [14,17-21]. Therefore, IF holds significant potential as a promising nonpharmacological approach to improving health at the population level [18]. We particularly focused on the simplicity and feasibility of IF, because a truly successful weight loss approach needs to be simple and sustainable, capable of being incorporated into daily life as lifelong dietary customs [18,21]. Since IF does not require detailed examination of food content or energy calculations, it is easy to implement with brief web-based guidance, having a high possibility of being applied as a cost-effective population approach. However, to the best of our knowledge, no studies have implemented IF as a web-based intervention, leaving a potentially significant solution to obesity unexplored.

To assess the effect of web-based IF administration, we have developed the “Brief Online Intermittent Fasting Program (OIF),” which features self-led fasting practice supported with meal delivery, online guidance, and app messages. The purposes of this pilot study were to evaluate the preliminary effectiveness, feasibility, and safety of the 12-week OIF on weight loss in adults with overweight and obesity in Japan. Secondary objectives were to assess its effects on body composition and metabolic markers.

## Methods

### Study Design

This pilot study is a 1:1 parallel-group randomized controlled trial (RCT) comparing the intervention group following the OIF with the control group receiving minimal care.

### Participants and Setting

#### Eligibility Criteria

In order to benefit from weight reduction and ensure the safe and stable administration of the OIF, the following eligibility criteria were established.

The inclusion criteria are as follows: individuals with overweight or obesity and with a BMI between 23 and under 35, adults aged 20 years to less than 65 years at the time of consent, those capable of communicating in Japanese, owners of a smartphone with iOS or Android, those who were familiar with using apps on a smartphone and using email and Zoom (Zoom Video Communications, Inc) on either a smartphone or a personal computer, those willing to visit one of the research facilities twice, and consenting to provide blood samples through self-puncture at the measurements. We defined overweight as having a BMI of 23 or above, while the global standard for overweight is a BMI of 25 or above. This adjustment aligns with guidelines from the National Institute for Health and Care Excellence, which recommend lowering the overweight threshold to 23 for Asian populations to enhance metabolic disease prevention in populations with lower obesity prevalence [22].

The exclusion criteria include any of the following: individuals with a history of heart disease, kidney disease, psychiatric disorders, or any serious medical conditions, individuals using diabetes medication (to minimize the risk of hypoglycemia), smokers or heavy drinkers (consuming alcohol on average more than 4 drinks per day or 14 drinks per week), night shift workers, individuals who may face significant environmental changes during the trial period, individuals with eating disorders, food allergies or alcohol dependence who require specific dietary supervision, women who are breastfeeding, pregnant, or planning to become pregnant during the study, lean individuals with a body fat percentage below 10% for men and 20% for women, athletes engaging in more than 12 hours of training or sports practice per week or those belonging to university sports clubs or professional teams aiming for competition, and any other individuals deemed unsuitable for participation by the researchers.

#### Setting and Recruitment Procedure

Participant recruitment, measurement, and registration were conducted at Kyoto University and 3 collaborating facilities in Japan: Kusaka Hospital, Cold Storage Japan Inc, and Buddy Training Co, Ltd. We recruited participants through our website (Department of Preventive Services at School of Public Health, Kyoto University), our X (formerly Twitter; X Corp), posters and flyers, and the OReC—a web platform dedicated to research recruitment [23]. Further details on the recruitment procedure and materials are presented in Multimedia Appendix 1.

### Sample Size

As this is a pilot RCT, a formal power-based sample size calculation for definitive hypothesis testing was not the primary objective. The main goals were to obtain estimates of effect size to inform the design of a future, larger trial. Based on recommendations for pilot study sample sizes [24], a range of 10-30 participants per group is often considered sufficient for these purposes. To ensure our sample size was within a reasonable range, we referred to previous literature [25,26] and pre-estimated the effect size to be approximately Cohen *d*=0.5. A sample of 10 participants per group would provide a preliminary indication of such an effect. To account for the potentially high dropout rate common in web-based intervention trials, we determined our target sample size to be 20 participants per group, for a total of 40 participants.

### Randomization

#### Allocation

The researcher (TN) weekly sent the information (ID, sex, and age) of the registered participants to another researcher (KK) via email. He conducted a 1:1 stratified randomization using computer-generated random sequences, with sex (male or female) and age (<40 or ≥40 years) as stratifying factors. The block sizes were randomly chosen to be either 2 or 4. Allocation concealment was maintained throughout the study.

#### Blinding

Participants were not blinded due to the nature of the dietary intervention. The implementer of the intervention was also not blinded since it was carried out by the researcher (TN), who knew the allocation results. Although the messages sent via app were also administered by TN and not blinded, the timing of delivery and the number of characters in the messages were standardized across both groups. However, to minimize potential bias, outcome assessors and a statistician analyst were blinded, as suggested by best practices in behavioral studies [27].

### Intervention

A nonblinded researcher (TN) administered all the interventions for both groups. Detailed information on interventions was found in Multimedia Appendix 2.

#### Intervention Group (OIF)

**Figure 1:**
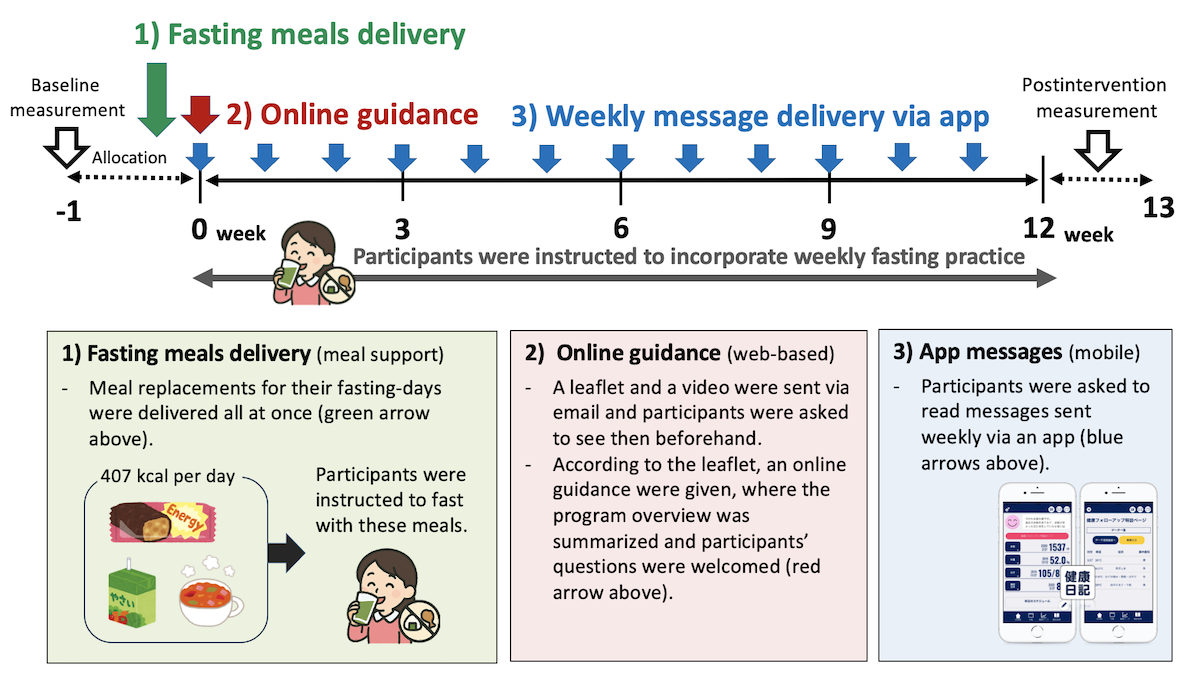
Overview of the 12-week “Brief Online Intermittent Fasting Program (OIF).”.

Participants in the intervention group were engaged in the OIF (Figure 1). In this study, fasting was defined as consuming the provided fasting meals by 8 PM without any additional food intake. The OIF comprises three components: (1) meal support (nonweb-based component), (2) a web-based component, and (3) a mobile component. Further details are described in Multimedia Appendix 2.

1. Fasting meals delivery: After the allocation, a 12-day supply of fasting meals was delivered to the intervention group in one batch. Fasting meals consisted of commercially available products that were low in energy, low in saturated fat, and plant-based and were constructed based on previous studies [28,29]. Briefly, it included 1 bottle of tomato juice, 2 bottles of fruit and vegetable juice, 2 servings of instant miso soup, 1 soy bar, and 1 multivitamin-mineral supplement, totaling 407 kcal per day, with 13% protein, 26% fat, and 61% carbohydrates. We developed this 12-week regimen in order to theoretically promote weight loss of 2.4-2.7 kg, which corresponds to a 3% loss of initial weight for a potential participant with a weight of 80 kg and 90 kg, respectively.
2. Online fasting guidance: We sent participants a leaflet detailing OIF (an A4 sheet, double-sided) via email upon allocation. They were directed to watch the prerecorded video (about 9 minutes) before the online guidance and then attended the guidance session via Zoom, which was held on their first day of the trial period and averaged 3.5 (SD 4.1) minutes per participant. They were instructed to replace one day’s meals with the provided fasting meals, with a recommendation to fast on a holiday to minimize their burden.
3. Weekly message delivery via app: Participants were instructed to install an app, “Kenko-Nikki (Healthtech Laboratory [HTL], Inc)” [30], detailed in Multimedia Appendix 3. Participants received weekly messages in Japanese (mean 382, SD 126 characters) containing general health advice on diet, exercise, sleep, and encouragement and reminders for their fasting practice through the app.

#### Control Group—Minimal Care

Similar to the intervention group, participants were directed to read weekly messages containing general health advice on diet, exercise, and sleep. The content of these messages differed partly from those sent to the intervention group, as outlined in Multimedia Appendix 2.

### Outcome Definition and Measurement

#### Data Collection

##### Overview

Sex, age, medical history, current medical visits, and medication status were collected via a web form at the time of preregistration. This information was reverified at the baseline measurement, along with identity confirmation. The following assessment data were measured at 2 time points—baseline and after 12 weeks, with the exception of adverse events and mild symptoms, adherence, and participants’ feedback, which were measured once at 12 weeks, and of self-recorded weight and step counts and fasting status, which were supposed to be measured every day.

##### Anthropometric Measures

Body weight, fat mass, muscle mass, and basal metabolic rate (BMR) were measured by using the body composition analyzer (MC-780A-N; TANITA Corporation). Height was measured 3 times at the baseline, barefoot, using a portable height measure (Seca 213 l; Seca Japan), and the median value was used. The BMI was calculated based on these data.

##### Blood Pressure

Measurements of systolic blood pressure (SBP) and diastolic blood pressure (DBP) were taken twice on the upper arm opposite the dominant hand using a blood pressure monitor (HEM–7511T; OMRON Corporation) while the participant was seated. The average value of these two measurements was used.

##### Blood Biomarkers

Hemoglobin A_1c_ (HbA_1c_), triglycerides, total cholesterol, high-density-lipoprotein cholesterol (HDL-C), and low-density-lipoprotein cholesterol (LDL-C) were measured using a point-of-care biochemical analysis device (cobas b 101 plus; Roche Diagnostics KK). Blood sampling involved participants self-puncturing the fingertip of the middle finger on the opposite side of their dominant hand using a spring-loaded lancet (Nipro LS lancet 30G 1.0 mm; Nipro Corporation). Subsequently, research staff aspirated the blood onto the reagent disk for analysis in the device.

##### Quality of Life

Quality of life (QOL) scores were also evaluated, which were measured using an electronic scoring system for the Short-Form 12-Item Survey-version 2 (SF-12v2) Japanese version [31] (Qualitest). Participants responded to questions on the web system regarding their lives over the past month via their smartphones. These responses were then calculated through the system to obtain physical and mental 2-component summary scores, which are standardized T-scores with a mean of 50 (SD 10), where higher scores indicate better health.

To assess participants’ subjective health status, responses to the first question of the SF-12v2 questionnaire, “In general, would you say your health is:,” were further analyzed. Response options included “(1) Excellent,” (2) Very good,” (3) Good,” (4) Unsatisfactory,” and “(5) Poor.”

##### Physical Activity

Participants’ physical activities were self-reported using the short form of the International Physical Activity Questionnaire [32]. The questions were prepared on a Google Form, and participants were asked to respond via smartphone about their physical activity over the past month at the time of final measurement. The amount of total physical activity was calculated as metabolic equivalent of task-minutes per week (METs).

##### Adverse Events and Mild Symptoms

For the evaluation of the safety, the number of adverse events and the number of individuals experiencing them were investigated. Participants were instructed to consult anytime throughout the study regarding any adverse events or symptoms. In addition, they were asked at the 12-week measurement about the incidence of 20 symptoms prelisted from a prior study [33] (sleep difficulty, hunger, fatigue, headache, diarrhea, sensitivity to cold, dry mouth, back pain, bad breath, muscle pain, abdominal bloating, cravings, vertigo, blurred vision, restless leg, skin rash, nausea, palpitation, dyspepsia, and muscular cramp) via a web form. We primarily evaluated the difference in the number of these 20 events and the number of participants who reportedly experienced them. Other adverse events were also self-reported separately through a free-text web questionnaire at the measurement at 12 weeks.

##### Adherence

Adherence in the intervention group was defined by the number of actual fasts, based on self-report via a web questionnaire at the 12-week measurement. Adherence in the control group was defined by the read status of messages on the app, with data obtained from the server. The definition of adherence is detailed in the statistical analysis section.

##### Participants’ Feedback

Participants’ feedback on each intervention was collected through a web questionnaire at the 12-week measurement.

##### Self-Recorded Weight and Step Counts

Participants were directed to record their self-measured weight and step counts via the app every day throughout the trial, which was predetermined to be assessed only exploratorily.

##### Fasting Status

Participants in the intervention group were directed to record the following information on their fasting day: the date, the consumption status of each fasting meal (the extent to which they ate and whether they ate other food or not), and the planned date of the next fasting day. This was also to be exploratorily assessed.

#### Outcome

The primary outcome was the change in body weight. The changes in BMI, fat mass, muscle mass, BMR, SBP, DBP, HbA_1c_, triglyceride, HDL-C, and LDL-C were evaluated as secondary outcomes. After formal analysis execution, the number of participants achieving a minimal clinically important change (MIC) was added as an exploratory outcome for post hoc analysis.

### Statistical Analysis

#### Overview

The statistical analysis plan (Multimedia Appendix 4) was fixed before data locking. After finalizing the data, the precoded analysis script (R [R Foundation for Statistical Computing], version 4.3.2) was executed. Statistical estimations were presented with 95% CI. Our predetermined α level was .05. Missing data in outcomes were imputed using the Baseline Observation Carried Forward (BOCF) approach. As this was a pilot study, no formal interim analyses or prespecified stopping guidelines were planned.

#### Baseline Analysis and Definition of Analysis Population

Baseline characteristics were presented as the number and percentage for sex (male), while continuous variables were presented as median and IQR.

Under the intention-to-treat (ITT) principle, all the allocated participants were regarded as the primary analysis population (ITT).

The per protocol set (PPS) included individuals in the intervention group who adhered to the fasting regimen (reportedly had fasted as instructed at least 10 out of 12 times) and participants in the control group who adhered to the minimal care (had read at least 10 out of the 12 app messages, as confirmed by server data). Additionally, within the intervention group, participants who reportedly had fasted 10 or more times out of 12 in accordance with our instructions were defined as “adherent,” while those who reported fasting 12 times were categorized as “fully adherent.”

Safety analyses were conducted on the completers set (CS), defined as participants who were followed up at the 12-week measurement.

In the discussion, we also presented the mean (SD) values of QOL scores for considering generalizability. Additionally, we assessed the subjective health status of participants by analyzing responses to the SF-12v2 questionnaire and comparing them with those from a similar question in a national annual survey conducted in 2022 [34]. We compare the proportions of participants who self-reported positive health status in the SF-12v2 questionnaire’s first question, “In general, would you say your health is:” with those from the reference survey, which asked, “How do you rate your current health status?” with similar responses ranging from 1 to 5. In the SF-12v2, the response options were “(1) Excellent, (2) Very good, (3) Good, (4) Unsatisfactory, (5) Poor,” while in the reference survey, they were “(1) Good, (2) Satisfactory, (3) Average, (4) Unsatisfactory, (5) Poor.” Recognizing the differences in response expression between the 2 surveys, we focused on respondents who considered themselves healthy, combining responses categorized as 1-3. This comparison was predetermined in the protocol.

#### Analysis of Primary Outcome

The change in body weight was presented as the mean and SD for each group. The primary analysis was to estimate the between-group mean difference under the ITT principle, adjusted for sex, age, and baseline weight using a generalized linear regression model, presented along with a 95% CI and *P* value. The regression coefficients for each covariate were also reported with their 95% CIs. Sensitivity analyses included unadjusted comparisons of crude values (2-tailed Welch *t* test) and per-protocol analysis within the PPS (using the same adjustment as the primary analysis). Additionally, in case of missing weight data, an analysis incorporating the worst-case scenario (imputing the maximum weight gain for the intervention group and the maximum weight loss for the control group from observed data) was predetermined to be added to the sensitivity analysis.

#### Analysis of Secondary Outcomes

For continuous secondary outcomes, crude (unadjusted) between-group mean differences and 95% CIs were calculated using the Welch *t* test. Total physical activity was also assessed to identify within-group differences, using the Wilcoxon signed-rank test for paired comparisons. In the safety analysis, the number of adverse events and the number of individuals experiencing them were investigated, and the between-group differences in incidence proportions were analyzed by the chi-square test. Additionally, within-group differences of QOL were analyzed by the Wilcoxon signed-rank test. The proportion of follow-up rate, adherence rate, and message-read rate was presented as percentages of the ITT population.

#### Exploratory Analysis

Self-recorded weight and step counts were predetermined to be assessed for discussion as needed, in cases of high dropout or missing data, for instance. As a post hoc analysis for the primary outcome, the number of participants achieving a MIC was compared between groups, with *P* values reported from the Fisher exact test. In addition, we included an additional sensitivity analysis for CS, which only analyzed the participants who completed the 12-week follow-up.

### Methods Alteration After Study Commencement

After the recruitment began, 3 collaborative facilities were added, and the initial eligibility criterion of having a BMI between 25 and less than 35 was modified to a BMI between 23 and less than 35. This change required a revision of the protocol and a subsequent review and reapproval by the Ethics Committee. Aspects of the statistical analysis not explicitly outlined in the original protocol were detailed in the statistical analysis plan (Multimedia Appendix 4), with its final version being fixed prior to the data lock.

### Ethical Considerations

This study was conducted with the approval of the Kyoto University Graduate School and Faculty of Medicine Ethics Committee and under the authorization of the dean (approval number C1625). The study was registered in the University Hospital Medical Information Network-Clinical Trials Registry (UMIN-CTR), a clinical trial registration service that meets the standards of the International Committee of Medical Journal Editors, on February 27, 2023 (UMIN000050437). Written informed consent was obtained from all participants. To protect privacy, participant data were pseudonymized. The correspondence table linking personal information to the data was stored on a separate USB drive and kept in a locked cabinet within a locked room. This table was deleted after the analysis to prevent reidentification. Participants received a 5000 Japanese yen Amazon gift card as compensation. Other ethical considerations and the consent document were described in Multimedia Appendix 1. This trial was conducted in accordance with the CONSORT (Consolidated Standards of Reporting Trials) 2025 and CONSORT-EHEALTH (Consolidated Standards of Reporting Trials of Electronic and Mobile Health Applications and Online Telehealth) guidelines (Multimedia Appendix 5). There was no patient or public involvement in the design, conduct, or reporting of the study.

## Results

### Participants Flow

Participant recruitment took place from July 20, 2023, to October 10, 2023. The follow-up measurement of all the participants ended on January 12, 2024.

**Figure 2:**
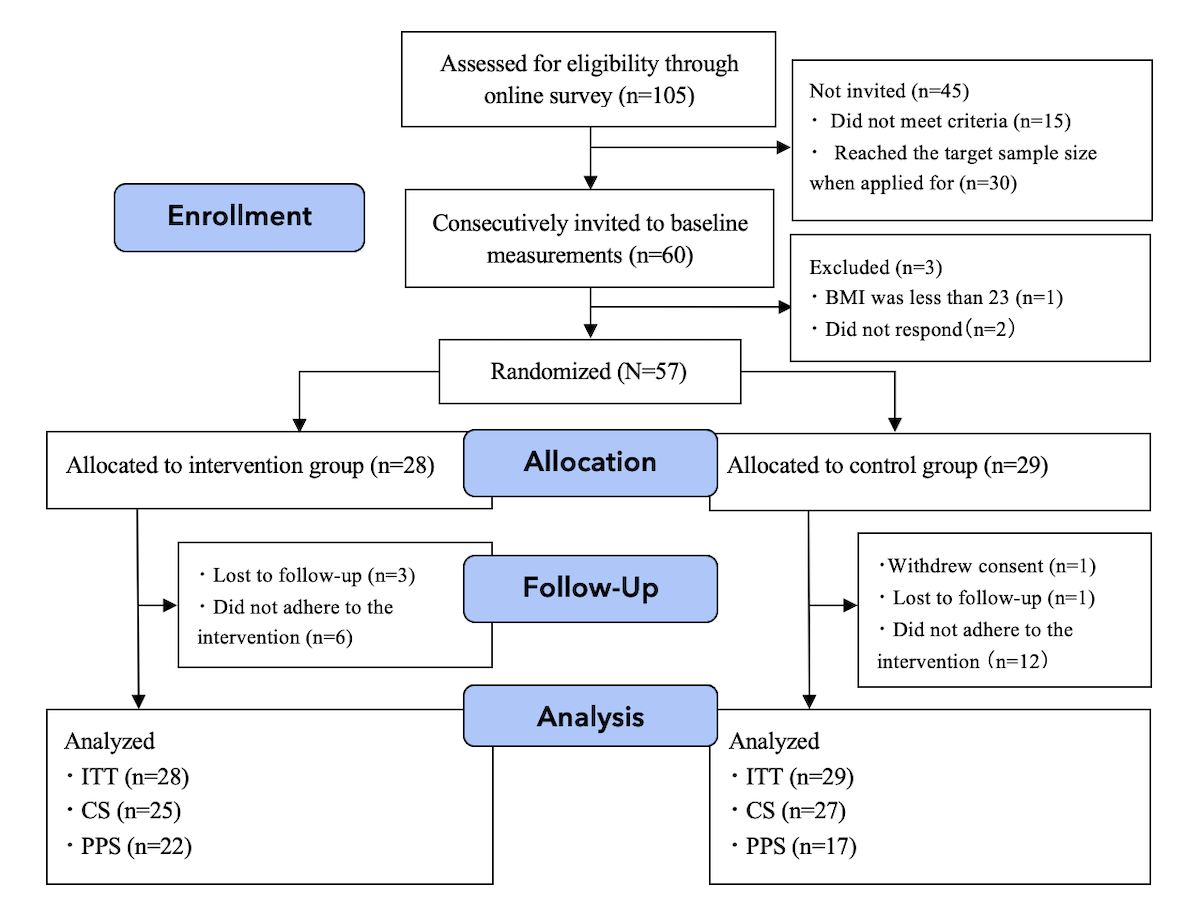
Participants flowchart. CS: completers set; ITT: intention-to-treat; PPS: per protocol set.

Among the 60 individuals invited to the baseline measurement, 57 were eventually registered, with 28 allocated to the intervention group and 29 to the control group (Figure 2). In the intervention group, 3 participants were lost to follow-up (dropped out) due to discontinued contact. In the control group, a participant withdrew consent due to “difficulties in continuing participation,” but agreed to our using his baseline data, allowing us to include him in the ITT population. Another participant was lost to follow-up (dropped out) in the control group. Consequently, of the 57 participants in the ITT population, 89% (25/28) in the intervention group and 93% (27/29) in the control group were followed up (defined together as the CS). The PPS was defined based on adherence, with 22 (vs 17) participants in the intervention (vs control) group. Among the 28 participants in the intervention group, 22 (79%) participants were defined as “adherent” (those who fasted 10 times or more; included in PPS), while 19 (68%) were defined as “fully adherent” (those who fasted 12 times). Among the 29 participants in the control group, 17 (59%) were adherent (who had read 10 app messages or more) and thus were included in PPS. Similarly, 15 (54%) participants in the intervention group had read app messages.

### Participants Characteristics

Table 1 presents the baseline characteristics of participants in each group. The proportion of men was high in both groups (19/28, 68% in the intervention group vs 19/29, 66% in the control group). The median age was 34 (IQR 29-47) vs 38 (IQR 29-52) years, the median body weight was 75.8 (IQR 68.3-80.6) vs 74.8 (IQR 69.8-81.8) kg, and the median BMI was 27.1 (IQR 24.8-29.8) vs 26.6 (IQR 24.7-28.5) for the intervention (vs control) group. The number of registered participants by facility was as follows: 22 (12 vs 10) participants at Kyoto University, 11 (7 vs 4) participants at Kusaka Hospital, 10 (4 vs 6) participants at Cold Storage Japan Inc, and 14 (5 vs 9) participants at Buddy Training Co, Ltd. Significant imbalances were not observed between the 2 groups across all characteristics.

**Table 1:** Baseline characteristics of the intervention and control groups (N=57)a.

Characteristic		Intervention (n=28)	Control (n=29)
Sex (male), n (%)		19 (68)	19 (66)
Age (years), median (IQR)		34 (29-47)	38 (29-52)
Weight (kg), median (IQR)		75.8 (68.3-80.6)	74.8 (69.8-81.8)
BMI (kg/cm^2^), median (IQR)		27.1 (24.8-29.8)	26.6 (24.7-28.5)
Height (cm), median (IQR)		168 (163-171)	170 (163-175)
Fat mass (kg), median (IQR)		22 (18-28)	22 (18-29)
Muscle mass (kg), median (IQR)		52 (42-56)	52 (41-57)
BMR^b^ (kcal), median (IQR)		1529 (1382-1682)	1563 (1369-1703)
SBP^c^ (mm Hg), median (IQR)		128 (118-141)	130 (117-142)
DBP^d^ (mm Hg), median (IQR)		87 (79-96)	90 (80-99)
HbA_1c_^e^ (%), median (IQR)		5.4 (5.2-5.6)	5.4 (5.2-5.5)
TG^f^ (mg/dL), median (IQR)		107 (70-209)	131 (111-208)
Total-C^g^ (mg/dL), median (IQR)		187 (165-212)	196 (176-220)
HDL-C^h^ (mg/dL), median (IQR)		57 (45-65)	57 (48-66)
LDL-C^i^ (mg/dL), median (IQR)		103 (89-122)	112 (80-128)
Total physical activity (METs^j^), median (IQR)		999 (399-1706)	1074 (827-1497)
**QOL^k^ score^l^, median (IQR)**			
	Physical component summary	50 (47-52)	52 (48-54)
	Mental component summary	59 (54-63)	55 (49-58)
**Subjective health status^m^, n (%)**			
	Excellent	3 (11)	3 (10)
	Very good	9 (32)	5 (17)
	Good	15 (54)	15 (52)
	Fair	0 (0)	5 (17)
	Poor	1 (4)	1 (3)
**Registered facility, n (%)**			
	Kyoto University	12 (43)	10 (35)
	Kusaka Hospital	7 (25)	4 (14)
	Cold Storage Japan Inc	4 (14)	6 (21)
	Buddy Training Co, Ltd.	5 (18)	9 (31)

^a^Data were presented as numbers for sex (male) and registered facilities, and as medians (IQR) for all other variables. The values were rounded for all the variables except for weight, BMI, and hemoglobin A_1c_.

^b^BMR: basal metabolic rate.

^c^SBP: systolic blood pressure.

^d^DBP: diastolic blood pressure.

^e^HbA_1c_: hemoglobin A_1c._

^f^TG: triglyceride.

^g^Total-C: total cholesterol.

^h^HDL-C: high-density-lipoprotein cholesterol.

^i^LDL-C: low-density-lipoprotein cholesterol.

^j^METs: metabolic equivalent of task-minutes per week.

^k^QOL: quality of life.

**^l^**SF-12v2 (Short-Form 12-Item Survey-version 2) Japanese version was used to obtain each 2-component summary score, which is standardized T-scores with a mean of 50 (SD 10), where higher scores indicate better health.

^m^Calculated from the responses to the first question of the SF-12v2 questionnaire, “In general, would you say your health is:” with options ranging from (1) Excellent, (2) Very good, (3) Good, (4) Unsatisfactory, and (5) Poor.

### Changes in Primary Outcome

#### Primary Analysis

Table 2 shows the primary results of this study. After 12 weeks, the mean weight change in the intervention group was –0.9 (SD 1.9) kg, while it was 0.6 (SD 1.4) kg in the control group. The primary analysis revealed an adjusted mean difference of –1.6 (95% CI –2.5 to –0.8; *P*<.001) kg. The regression coefficients for covariates were as follows: baseline weight=–0.2 (95% CI –0.6 to 0.2) kg per 10-kg increase; sex (male)=0.5 (95% CI –0.5 to 1.4) kg; and age=–0.4 (95% CI –0.7 to 0.0) kg per 10-year increase.

**Table 2:** The effect of the intervention between the intervention and control groups (N=57)a.

Variable	Intervention (n=28)	Control (n=29)	Adjusted mean difference (95% CI)^b^	*P* value
Weight at baseline (kg), median (IQR)	75.8 (68.3-80.6)	74.8 (69.8-81.8)	—^c^	—
Weight at 12 weeks (kg), median (IQR)	74.5 (66.3-79.5)	74.8 (69.1-83.1)	—	—
Weight change (kg), mean (SD)	–0.9 (1.9)	0.6 (1.4)	–1.6 (–2.5 to –0.8)^b^	<.001

^a^Intention-to-treat analysis. Data were presented as median (IQR) for each measured weight and as mean (SD) for the weight change.

^b^Adjusted for stratifying factors (age and sex) and baseline weight by using the generalized linear regression model.

^c^Not applicable.

#### Sensitivity Analysis

Among the sensitivity analyses, the crude comparison showed a mean difference of –1.5 (95% CI –2.4 to –0.7; *P*=.001) kg, and the per-protocol analysis revealed a mean difference of –1.8 (95% CI –2.9 to –0.6; *P*=.004) kg. Additional sensitivity analysis for CS presented similar results; a mean difference of –1.8 (95% CI –2.7 to –0.9; *P*=.004) kg. In a worst-case scenario, where missing data were imputed (+3.3 kg to the weight change for the 3 missing participants in the intervention group and –4.7 kg to the weight change for the 2 missing in the control group), the adjusted mean difference was –1.0 (95% CI –2.1 to 0.2; *P*=.10) kg, and the crude mean difference was –0.9 (95% CI –2.0 to 0.3; *P*=.13) kg.

#### Exploratory Analysis

The post hoc analysis revealed that more participants (8/28, 29%; *P*=.01) achieved a MIC (3% reduction in initial weight) in the intervention group compared to the control group (1/29, 3%).

### Changes in Secondary Outcomes

#### Changes in Continuous Variables

The results for secondary outcomes were presented in Table 3, showing the within-group changes and between-group differences. In line with weight changes, the BMI decreased more in the intervention group compared to the control group (mean difference=–0.4, 95% CI –0.1 to –0.8 kg). However, no significant difference was observed in fat mass changes (mean difference=–0.1, 95% CI –1.3 to 1.4 kg), whereas a decrease in muscle mass was implied (mean difference=–1.3, 95% CI –2.5 to –0.2 kg). In alignment with the changes in body composition, a decrease was suggested in the BMR (mean difference=–37.8, 95% CI –67.5 to –8.2 kcal). No significant differences were observed in other outcomes. The mean differences (95% CI) for each outcome were as follows: SBP= –4.6 (–10.6 to 1.3) mm Hg, DBP=–1.1 (–5.6 to 3.3) mm Hg, HbA_1c_=–0.0 (–0.2 to 0.1) %, TG=–30.8 (–71.3 to 9.7) mg/dL, total cholesterol=–8.2 (–22.7 to 6.4) mg/dL, HDL-C=2.2 (–2.7 to 7.1) mg/dL, LDL-C=–4.1 (–17.1 to 8.9) mg/dL, physical QOL=0.6 (–2.6 to 3.9), mental QOL=–0.7 (–4.0 to 2.3), subjective health status=–0.2 (–0.5 to 0.3), and total physical activity=291 (–508 to 1090) METs.

**Table 3:** The changes in secondary outcomes from baseline to 3 months. (N=57)a.

Variable			Intervention (n=28), mean (SD)		Control (n=29), mean (SD)		Between-group difference^b^, mean difference (95% CI)
BMI (kg/cm^2^)			–0.3 (0.7)		0.2 (0.5)		–0.4 (–0.8 to –0.1)
Fat mass (kg)			–0.2 (1.7)		–0.3 (3.1)		0.1 (–1.3 to 1.4)
Muscle mass (kg)			–0.6 (1.0)		0.8 (2.8)		–1.3 (–2.5 to –0.2)
BMR^c^ (kcal)			–17.9 (31.9)		19.9 (72.0)		–37.8 (–67.5 to –8.2)
SBP^d^ (mm Hg)			–3.3 (9.4)		1.3 (12.7)		–4.6 (–10.6 to 1.3)
DBP^e^ (mm Hg)			–3.6 (6.6)		–2.4 (9.8)		–1.1 (–5.6 to 3.3)
HbA_1c_^f^ (%)			–0.1 (0.2)		–0.0 (0.3)		0.0 (–0.2 to 0.1)
TG^g^ (mg/dL)			1.1 (64.8)		31.9 (86.4)		–30.8 (–71.3 to 9.7)
Total-C^h^ (mg/dL)			3.4 (24.4)		11.6 (30.2)		–8.2 (–22.7 to 6.4)
HDL-C^i^ (mg/dL)			2.4 (7.4)		0.2 (10.8)		2.2 (–71.3 to 9.7)
LDL-C^j^ (mg/dL)			0.9 (25.3)		5.0 (23.7)		–4.1 (–17.1 to 8.9)
**QOL^k^ score^l^**							
	Physical component summary	0.5 (5.8)		–0.1 (6.3)		0.6 (–2.6 to 3.9)	
	Mental component summary	–0.5 (6.8)		0.2 (4.6)		–0.7 (–4.0 to 2.3)	
Subjective health status^m^			–0.3 (0.6)		–0.1 (0.9)		–0.2 (–0.5 to 0.3)
Total physical activity (METs^n^)			350 (1679)		59 (1295)		291 (–508 to 1090)

^a^Intention-to-treat analysis. Data were presented as mean (SD) for the change in each outcome and as mean difference (95% CI) for the difference between groups in the change scores.

^b^The difference in secondary outcomes between groups was not adjusted and was presented as crude values, with 95% CI of the Welch *t* test.

^c^BMR: basal metabolic rate.

^d^SBP: systolic blood pressure.

^e^DBP: diastolic blood pressure.

^f^HbA_1c_: hemoglobin A_1c._

^g^TG: triglyceride.

^h^Total-C: total cholesterol.

^i^HDL-C: high-density-lipoprotein cholesterol.

^j^LDL-C: low-density-lipoprotein cholesterol.

^k^QOL: quality of life.

^l^SF-12v2 (Short-Form 12-Item Survey-version 2) Japanese version was used to obtain each 2-component summary score, which is standardized T-scores with a mean of 50 (SD 10), where higher scores indicate better health.

^m^Calculated from the responses to the first question of the SF-12v2 questionnaire, “In general, would you say your health is:” with options ranging from (1) Excellent, (2) Very good, (3) Good, (4) Unsatisfactory, and (5) Poor.

^n^METs: metabolic equivalents-minutes per week.

#### Safety Analysis

Table 4 displays the reported number of events and the incidence—the number of participants who experienced it—for each of the 20 mild symptoms. A larger proportion of participants reported hunger in the intervention group (22/25, 89%; *P*<.001) compared to the control group (3/27, 11%). Additionally, fatigue (16/25, 64% vs 6/27, 22%; *P*=.06) and cravings (15/25, 60% vs 3/27, 11%; *P*=.01) were also more reported in the intervention group (vs control group). On the other hand, the participants in the control group reported hunger (43 vs 27) and cravings (70 vs 42) more frequently compared to the intervention group. Adverse events reported in free text included influenza-like illness (1/25 vs 3/27), sinusitis (0/25 vs 1/27), viral gastroenteritis (1/25 vs 0/27), loss of appetite (1/25 vs 3/27), and drowsiness (2/25 vs 0/27). Regarding the within-group difference in QOL scores, no significant changes were observed before and after in either group: physical QOL (intervention: mean 0.51, SD 5.84 vs control: mean –0.13, SD 6.27), and the mental QOL (mean –0.54, SD 6.84 vs mean 0.15, SD 4.60).

**Table 4:** Self-reported mild symptoms in the intervention and control groups (N=52)a.

Mild symptoms	Intervention (n=25)			Control (n=27)			*P* value^b^
	Events	Incidence (%)	Events		Incidence (%)		
Hunger	27	22 (88)	43		3 (11)	<.001	
Fatigue	187	16 (64)	27		6 (22)	.01	
Headache	125	12 (48)	74		7 (25)	.17	
Cravings	42	15 (60)	70		3 (11)	.001	
Diarrhea	30	5 (20)	68		7 (25)	.86	
Dry mouth	118	8 (32)	68		4 (14)	.25	
Back pain	66	6 (24)	21		6 (22)	>.99	
Sensitivity to cold	36	5 (20)	25		6 (22)	>.99	
Muscle pain	24	4 (16)	4		5 (18)	>.99	
Abdominal bloating	9	4 (16)	14		5 (18)	>.99	
Vertigo	17	4 (16)	9		5 (18)	>.99	
Sleep difficulty	77	4 (16)	24		4 (14)	>.99	
Dyspepsia	13	2 (8)	57		5 (18)	.48	
Bad breath	7	4 (16)	12		2 (7)	.59	
Blurred vision	6	2 (8)	0		4 (14)	.74	
Nausea	0	3 (12)	13		2 (7)	.93	
Muscular cramp	9	3 (12)	8		2 (7)	.93	
Palpitation	1	1 (4)	7		3 (11)	.66	
Skin rash	22	0 (0)	21		3 (11)	.26	
Restless leg	4	2 (8)	2		0 (0)	.44	

^a^Analysis population was the completers set. Participants were asked to report the number of incidences of 20 mild symptoms listed in the web questionnaire by answering 0-90 times per symptom.

^b^*P* values were presented for the incidences.

## Discussion

### Principal Findings

This study aimed to evaluate the effectiveness, feasibility, and safety of a 12-week OIF among adults with overweight and obesity in Japan. Overall, the program demonstrated effectiveness in promoting modest weight loss over 12 weeks, with high feasibility and safety indicated by low dropout rates and absence of serious adverse events.

The participants in the intervention group tended to lose weight (mean –0.9, SD 1.9 kg), whereas the control group, on average, gained weight (mean 0.6, SD 1.4 kg). Generally, individuals with overweight tend to continue to gain weight [35], but the result suggests this increase can be mitigated by the OIF. The adjusted mean difference was –1.6 (95% CI –2.5 to –0.8) kg, and more participants achieved a MIC in the intervention group compared to the control group (8/28 vs 1/29 participants). Among the sensitivity analyses, the per-protocol analysis showed a subtle larger effect (mean difference –1.8, 95% CI –2.9 to –0.6 kg), implying the efficacy of the OIF. The effect size calculated by the weight change (SD) was large (Cohen *d*=0.95), but the effect size based on the after-weight (SD) was small (Cohen *d*=0.19).

The dropout rate was low in the intervention group (3/28, 11%). Additionally, adherence rate exceeded our expectations, with 79% (22/28) of “adherent” and 68% (17/28) of “fully adherent,” indicating a modest level of feasibility. However, there were also concerns regarding their self-reported mild symptoms. Based on the incidence, more participants reported hunger or cravings in the intervention group, although the participants in the control group reported them more frequently in terms of the number of events they experienced. There was a slight trend that participants in the intervention group had more headaches (both in events and incidence), but the result was not statistically significant. Except for hunger, fatigue, and cravings, there were no statistically significant differences in reported symptoms between the groups.

Concerning safety, the intervention showed encouraging indications. No serious adverse events were reported, and QOL remained stable (insignificant within-group differences in QOL score between before and after, and insignificant between-group differences in change in QOL) throughout the study. These findings, coupled with the modest adherence observed, suggest that the intervention is generally safe. Nonetheless, the intervention group reported more hunger, fatigue, and cravings, underscoring the importance of providing thorough explanations and follow-up.

### Comparison to Prior Work

Many studies have demonstrated the effectiveness of web-based weight loss interventions [7,8,25]. A previous RCT among adults with overweight in Japan showed a moderate weight loss effect from a 12-week app intervention (adjusted mean difference=–1.60, 95% CI –2.83 to –0.38; *P*=.01) [26]. A recent meta-analysis indicated that web-based interventions facilitate weight loss compared to minimal care control groups (mean difference=–1.40, 95% CI –1.98 to –0.82; *P*<.001) [8]. This study observed similar effects to these. However, we initially had expected a potential weight loss of 2.7 kg through the OIF, but it was 0.9 kg, and the observed between-group difference was –1.6 kg, with both changes falling short of the expectation. A possible reason for the slight weight loss in the intervention group and the weight gain in the control group could be attributed to the timing of the study. The study was conducted from September 2023 to January 2024, and most participants completed their follow-up measurements during the Christmas and New Year holidays, which possibly contributed to the weight gain [36]. Since total physical activity did not change, another possible reason should also lie in diet. Participants in the intervention group might feel hungrier, which is consistent with the previous studies [37,38], and might have compensated for the energy reduced by fasting through consuming more food on nonfasting days. This was implied by several participants’ feedback in the questionnaire. However, this is just speculative since prior works have demonstrated the lack of compensation for energy intake in IF [39-41], and this study did not collect information on a regular diet. The next study should consider comprehensive dietary assessments to elucidate these findings further.

This study showed no significant reduction in fat mass (mean difference=–0.1, 95% CI –1.3 to 1.4 kg) but a decrease in muscle mass (mean difference=–1.3, 95% CI –2.5 to –0.2 kg). However, it is natural that glycogen stored in muscles is used first during the initial phase of short-term weight loss, accompanied by a temporary decrease in water and muscle mass [42]. Thus, the observed reduction in muscle mass may not directly raise concerns. A systematic review and meta-analysis comparing conventional dietary restrictions with IF have not found evidence that IF promotes greater muscle loss [43]. Furthermore, other studies have shown that incorporating exercise during IF can increase muscle mass and exercise performance [44,45]. A recent systematic review has also concluded that combining IF with strength training can maintain lean body mass [46]. These findings suggest that the OIF does not necessarily worsen body composition, but on its own, it might not provide sufficient health benefits. Weight loss interventions should ideally include exercise therapy and comprehensive lifestyle modifications [47,48], a principle that we conclude also applies to online fasting interventions.

### Strengths and Limitations

One of the strengths of this study lies in its study design. A randomized controlled design using minimal care as a control enabled us to obtain more valid estimates than study designs with single-arm or no-intervention controls. The sample size that was large for a pilot study led to demonstrating statistically significant results for weight loss, justifying conducting future trials.

In addition, we comprehensively evaluated the effects of the intervention, measuring multiple outcomes, allowing us to obtain invaluable insights into our program. For example, no observed change in physical activity implied that the OIF might facilitate weight loss through reduced energy intake.

This study has several limitations. First, we lacked important data on physical activity and dietary habits. In this study, physical activity was measured by self-report and was considered to be of low accuracy, which might prevent us from detecting potential changes. Additionally, the lack of data on a regular diet limited the interpretation of the results. In particular, evaluating why weight loss was below expectations is quite challenging. Future studies should evaluate the physical activity and dietary habits more accurately in order to distinguish the efficacy of IF from other lifestyle factors.

Second, our analysis is limited by the way of handling the missing values. We used the BOCF approach to impute missing body weight data. However, since missing data on body weight after the intervention could occur systematically, this approach may have introduced bias and compromised our conclusions. Notably, the statistically significant between-group difference in weight change diminished when considering the worst-case scenario (imputing +3.3 kg of weight change for the 3 missing participants in the intervention group and –4.7 kg for the 2 missing participants in the control group). Nevertheless, we believe that the BOCF approach is unlikely to have significantly overestimated the intervention effect. Participants in the control group were unlikely to lose weight without intervention and might even have gained weight as previously reported [35], whereas those in the intervention group were more likely to achieve weight loss. Thus, imputing the outcome weight with the baseline value is more likely to lead to diluting the weight loss effect, not overestimating it. Furthermore, our analysis demonstrated that a greater proportion of participants in the intervention group achieved a clinically meaningful weight loss (MIC; a 3% reduction in initial weight) compared to the control group, supporting the effectiveness of the intervention.

Third, as for the participants’ recruitment, we used convenience sampling and cannot assume that it was a random sampling. Notably, participants included several direct acquaintances of the researchers (TN and TI). The proportion of participants considering themselves to be healthy was 88% (50/57), which is comparable to the results from the reference national survey [34], with 86.16% (91768/106507) for the total population and 89.66% (50816/56675) for those aged 20-65 years, implying that they were not far from the generally healthy population with overweight and obesity. However, our participants were exclusively Japanese, and it is unclear whether the findings would be applicable in other countries or regions with different lifestyles. Thus, the generalizability of the conclusions remains unclear. Nevertheless, we believe that our setting with multiple facilities possibly contributed to recruiting participants with a broad range of characteristics. In the future trial, we plan to broaden the generalizability of the findings by using systematic recruitment methods and conducting the study in other countries.

Finally, we did not systematically collect data on concomitant care that participants may have received during the trial, such as seeking external dietary advice or using other commercial weight-loss programs. Therefore, we cannot completely rule out the possibility that such unmeasured care could have influenced the results.

### Conclusions

The 12-week OIF can promote weight loss in apparently healthy adults with overweight and obesity; yet, it may not result in improvements in body composition. The observed low dropout rate and modest adherence, with no serious adverse events or significant changes in QOL, indicate the feasibility and safety of the program.

## Appendix

Multimedia Appendix 1 Supplementary information on participant recruitment.

Multimedia Appendix 2 Supplementary information on the interventions.

Multimedia Appendix 3 Supplementary information on research app.

Multimedia Appendix 4 Statistical analysis plan.

Multimedia Appendix 5 CONSORT 2025 checklist.

## Abbreviations

BMR: basal metabolic rate
BOCF: Baseline Observation Carried Forward
CONSORT: Consolidated Standards of Reporting Trials
CONSORT-EHEALTH: Consolidated Standards of Reporting Trials of Electronic and Mobile Applications and Online Telehealth
CS: completers set
DBP: diastolic blood pressure
HbA1c: hemoglobin A1c
HDL-C: high-density-lipoprotein cholesterol
HTL: Healthtech Laboratory, Inc
IF: intermittent fasting
ITT: intention-to-treat
LDL-C: low-density-lipoprotein cholesterol
METs: metabolic equivalent of task-minutes per week
MIC: minimal clinically important change
OIF: Brief Online Intermittent Fasting Program
PPS: per protocol set
QOL: quality of life
RCT: randomized controlled trial
SBP: systolic blood pressure
SF-12v2: Short-Form 12-Item Survey-version 2
UMIN-CTR: University Hospital Medical Information Network-Clinical Trials Registry

## Data Availability

The datasets analyzed during this study are available from the corresponding author on reasonable request.

## References

[1] Finucane MM, Stevens GA, Cowan MJ, Danaei G, Lin JK, Paciorek CJ, Singh GM, Gutierrez HR, Lu Y, Bahalim AN, Farzadfar F, Riley LM, Ezzati M, Global Burden of Metabolic Risk Factors of Chronic Diseases Collaborating Group (Body Mass Index) (2011). National, regional, and global trends in body-mass index since 1980: systematic analysis of health examination surveys and epidemiological studies with 960 country-years and 9·1 million participants. Lancet.

[2] Obesity. World Health Organization.

[3] Arroyo-Johnson C, Mincey KD (2016). Obesity epidemiology worldwide. Gastroenterol Clin North Am.

[4] Bakaloudi DR, Barazzoni R, Bischoff SC, Breda J, Wickramasinghe K, Chourdakis M (2022). Impact of the first COVID-19 lockdown on body weight: a combined systematic review and a meta-analysis. Clin Nutr.

[5] Fraticelli F, Nicola MD, Vitacolonna E (2023). A nutritional web-based approach in obesity and diabetes before and during the COVID-19 lockdown. J Telemed Telecare.

[6] Ammar A, Brach M, Trabelsi K (2020). Effects of COVID-19 home confinement on eating behaviour and physical activity: results of the ECLB-COVID19 international online survey. Nutrients.

[7] Hutchesson MJ, Rollo ME, Krukowski R, Ells L, Harvey J, Morgan PJ, Callister R, Plotnikoff R, Collins CE (2015). eHealth interventions for the prevention and treatment of overweight and obesity in adults: a systematic review with meta-analysis. Obes Rev.

[8] Sorgente A, Pietrabissa G, Manzoni GM, Re F, Simpson S, Perona S, Rossi A, Cattivelli R, Innamorati M, Jackson JB, Castelnuovo G (2017). Web-based interventions for weight loss or weight loss maintenance in overweight and obese people: a systematic review of systematic reviews. J Med Internet Res.

[9] Akers JD, Estabrooks PA, Davy BM (2010). Translational research: bridging the gap between long-term weight loss maintenance research and practice. J Am Diet Assoc.

[10] Ye Y, Zhang M, Lin Z, Tang L (2022). Is intermittent fasting better than continuous energy restriction for adults with overweight and obesity?. Diabetes Metab Syndr Obes.

[11] Anton SD, Moehl K, Donahoo WT, Marosi K, Lee SA, Mainous AG, Leeuwenburgh C, Mattson MP (2018). Flipping the metabolic switch: understanding and applying the health benefits of fasting. Obesity (Silver Spring).

[12] Allaf M, Elghazaly H, Mohamed O, Fareen MFK, Zaman S, Salmasi A-M, Tsilidis K, Dehghan A (2021). Intermittent fasting for the prevention of cardiovascular disease. Cochrane Database Syst Rev.

[13] Morales-Suarez-Varela M, Collado Sánchez E, Peraita-Costa I, Llopis-Morales A, Soriano JM (2021). Intermittent fasting and the possible benefits in obesity, diabetes, and multiple sclerosis: a systematic review of randomized clinical trials. Nutrients.

[14] de Cabo R, Mattson MP (2019). Effects of intermittent fasting on health, aging, and disease. N Engl J Med.

[15] Klempel MC, Kroeger CM, Bhutani S, Trepanowski JF, Varady KA (2012). Intermittent fasting combined with calorie restriction is effective for weight loss and cardio-protection in obese women. Nutr J.

[16] Kroeger CM, Klempel MC, Bhutani S, Trepanowski JF, Tangney CC, Varady KA (2012). Improvement in coronary heart disease risk factors during an intermittent fasting/calorie restriction regimen: relationship to adipokine modulations. Nutr Metab (Lond).

[17] Di Francesco A, Di Germanio C, Bernier M, de Cabo R (2018). A time to fast. Science.

[18] Patterson RE, Sears DD (2017). Metabolic effects of intermittent fasting. Annu Rev Nutr.

[19] Jamshed H, Beyl R, Della Manna D, Yang E, Ravussin E, Peterson C (2019). Early time-restricted feeding improves 24-Hour glucose levels and affects markers of the circadian clock, aging, and autophagy in humans. Nutrients.

[20] Manoogian EN, Panda S (2017). Circadian rhythms, time-restricted feeding, and healthy aging. Ageing Res Rev.

[21] Wilhelmi de Toledo F, Grundler F, Sirtori CR, Ruscica M (2020). Unravelling the health effects of fasting: a long road from obesity treatment to healthy life span increase and improved cognition. Ann Med.

[22] (2016). Obesity in adults: prevention and lifestyle weight management programmes. National Institute for Health and Care Excellence.

[23] Open Research Cafe.

[24] Whitehead AL, Julious SA, Cooper CL, Campbell MJ (2016). Estimating the sample size for a pilot randomised trial to minimise the overall trial sample size for the external pilot and main trial for a continuous outcome variable. Stat Methods Med Res.

[25] Muramoto A, Matsushita M, Kato A, Yamamoto N, Koike G, Nakamura M, Numata T, Tamakoshi A, Tsushita K (2014). Three percent weight reduction is the minimum requirement to improve health hazards in obese and overweight people in Japan. Obes Res Clin Pract.

[26] Nakata Y, Sasai H, Gosho M, Kobayashi H, Shi Y, Ohigashi T, Mizuno S, Murayama C, Kobayashi S, Sasaki Y (2022). A smartphone healthcare application, CALO mama Plus, to promote weight loss: a randomized controlled trial. Nutrients.

[27] Hróbjartsson A, Thomsen ASS, Emanuelsson F, Tendal B, Hilden J, Boutron I, Ravaud P, Brorson S (2012). Observer bias in randomised clinical trials with binary outcomes: systematic review of trials with both blinded and non-blinded outcome assessors. BMJ.

[28] Wei M, Brandhorst S, Shelehchi M, Mirzaei H, Cheng CW, Budniak J, Groshen S, Mack WJ, Guen E, Di Biase S, Cohen P, Morgan TE, Dorff T, Hong K, Michalsen A, Laviano A, Longo VD (2017). Fasting-mimicking diet and markers/risk factors for aging, diabetes, cancer, and cardiovascular disease. Sci Transl Med.

[29] Wilhelmi de Toledo F, Buchinger A, Burggrabe H, Hölz G, Kuhn C, Lischka E, Lischka N, Lützner H, May W, Ritzmann-Widderich M, Stange R, Wessel A, Boschmann M, Peper E, Michalsen A, Medical Association for FastingNutrition (Ärztegesellschaft für Heilfasten und Ernährung‚ ÄGHE (2013). Fasting therapy - an expert panel update of the 2002 consensus guidelines. Forsch Komplementmed.

[30] "Health Diary" is a free app that records your daily health status. Healthtech.Lab Inc.

[31] Ware J, Kosinski M, Keller SD (1996). A 12-item short-form health survey: construction of scales and preliminary tests of reliability and validity. Med Care.

[32] Craig CL, Marshall AL, Sjöström M, Bauman AE, Booth ML, Ainsworth BE, Pratt M, Ekelund U, Yngve A, Sallis JF, Oja P (2003). International physical activity questionnaire: 12-country reliability and validity. Med Sci Sports Exerc.

[33] Wilhelmi de Toledo F, Grundler F, Bergouignan A, Drinda S, Michalsen A (2019). Safety, health improvement and well-being during a 4 to 21-day fasting period in an observational study including 1422 subjects. PLoS One.

[34] (2022). Overview of the 2022 (Reiwa 4) comprehensive survey on living conditions of the people. Ministry of Health, Labour and Welfare.

[35] Katsoulis M, Lai AG, Diaz-Ordaz K, Gomes M, Pasea L, Banerjee A, Denaxas S, Tsilidis K, Lagiou P, Misirli G, Bhaskaran K, Wannamethee G, Dobson R, Batterham RL, Kipourou D, Lumbers RT, Wen L, Wareham N, Langenberg C, Hemingway H (2021). Identifying adults at high-risk for change in weight and BMI in England: a longitudinal, large-scale, population-based cohort study using electronic health records. Lancet Diabetes Endocrinol.

[36] Helander EE, Wansink B, Chieh A (2016). Weight gain over the holidays in three countries. N Engl J Med.

[37] Sundfør T M, Svendsen M, Tonstad S (2018). Effect of intermittent versus continuous energy restriction on weight loss, maintenance and cardiometabolic risk: a randomized 1-year trial. Nutr Metab Cardiovasc Dis.

[38] Heilbronn LK, Smith SR, Martin CK, Anton SD, Ravussin E (2005). Alternate-day fasting in nonobese subjects: effects on body weight, body composition, and energy metabolism. Am J Clin Nutr.

[39] Huang A, Henderson G, Profeta A, Pfeiffer M, Feinstein LH, deLahunta M, LaHood C, Michael JJ, Mizia AC, Levitsky DA (2023). Lack of compensation of energy intake explains the success of alternate day feeding to produce weight loss. Physiol Behav.

[40] Varady KA, Bhutani S, Klempel MC, Kroeger CM, Trepanowski JF, Haus JM, Hoddy KK, Calvo Y (2013). Alternate day fasting for weight loss in normal weight and overweight subjects: a randomized controlled trial. Nutr J.

[41] Ravussin E, Beyl RA, Poggiogalle E, Hsia DS, Peterson CM (2019). Early time-restricted feeding reduces appetite and increases fat oxidation but does not affect energy expenditure in humans. Obesity (Silver Spring).

[42] Denke MA (2001). Metabolic effects of high-protein, low-carbohydrate diets. Am J Cardiol.

[43] Enríquez Guerrero A, San Mauro Martín I, Garicano Vilar E, Camina Martín MA (2021). Effectiveness of an intermittent fasting diet versus continuous energy restriction on anthropometric measurements, body composition and lipid profile in overweight and obese adults: a meta-analysis. Eur J Clin Nutr.

[44] Keenan SJ, Cooke MB, Hassan EB, Chen WS, Sullivan J, Wu SX, El-Ansary D, Imani M, Belski R (2022). Intermittent fasting and continuous energy restriction result in similar changes in body composition and muscle strength when combined with a 12 week resistance training program. Eur J Nutr.

[45] Martínez-Rodríguez A, Rubio-Arias JA, García-De Frutos JM, Vicente-Martínez M, Gunnarsson TP (2021). Effect of high-intensity interval training and intermittent fasting on body composition and physical performance in active women. Int J Environ Res Public Health.

[46] Keenan S, Cooke MB, Belski R (2020). The effects of intermittent fasting combined with resistance training on lean body mass: a systematic review of human studies. Nutrients.

[47] Hall ME, Cohen JB, Ard JD, Egan BM, Hall JE, Lavie CJ, Ma J, Ndumele CE, Schauer PR, Shimbo D, American Heart Association Council on Hypertension; Council on Arteriosclerosis‚ ThrombosisVascular Biology; Council on LifestyleCardiometabolic Health;Stroke Council (2021). Weight-loss strategies for prevention and treatment of hypertension: a scientific statement from the American heart association. Hypertension.

[48] Wharton S, Lau DC, Vallis M, Sharma AM, Biertho L, Campbell-Scherer D, Adamo K, Alberga A, Bell R, Boulé N, Boyling E, Brown J, Calam B, Clarke C, Crowshoe L, et al (2020). Obesity in adults: a clinical practice guideline. CMAJ.

