# Supplementary material for: A Brief Web-Based and Mobile Intervention of Intermittent Fasting With Meal Support for Weight Loss Among Adults With Overweight and Obesity in Japan: Pilot Randomized Controlled Trial
Source: JMIR Mhealth Uhealth. 2026 Jan 26;14:e58930. doi: 10.2196/58930 (PMC12887555; doi:10.2196/58930)
Supplement: Multimedia Appendix 1 [file mhealth_v14i1e58930_app1.docx]

A Brief Web-Based Intervention of Intermittent Fasting with Meal Support for Weight Loss Among Overweight Japanese Adults: A Pilot Study of a Randomized Controlled Trial

**Multimedia Appendix 1:**

**Supplementary Information on Participants Recruitment**

This is a Multimedia Appendix to a full manuscript published in the J Med Internet Res. For full copyright and citation information see <http://dx.doi.org/10.2196/jmir.58930>

[Ⅰ. Recruitment Procedure 2](#_Toc160268830)

[Ⅱ. Recruitment Flyer and Poster 2](#_Toc160268831)

[Figure S1-1. English translated version of the recruitment poster. 3](file:////Users/nodapai2020/Documents/研究/論文投稿初回/MA1_Supplementary_Information_on_the_Participants_Recruitment.docx#_Toc160268832)

[Figure S1-2. Original Japanese version of the recruitment poster. 4](file:////Users/nodapai2020/Documents/研究/論文投稿初回/MA1_Supplementary_Information_on_the_Participants_Recruitment.docx#_Toc160268833)

[Ⅲ. Ethical Considerations 5](#_Toc160268834)

[Ⅳ. Informed Consent Document 5](#_Toc160268835)

[1. English version 5](#_Toc160268836)

[2. Original Japanese version 11](#_Toc160268837)

## Ⅰ. Recruitment Procedure

Participant recruitment, measurement, and registration were conducted at Kyoto University and three collaborating facilities in Japan: Kusaka Hospital (Mie), Cold storage Japan Inc. (Tokyo), and BUDDY TRAINING Co., Ltd. (Tokyo). We recruited participants through our [website](https://yobou.med.kyoto-u.ac.jp/) (Department of Preventive Services at School of Public Health, Kyoto University), our [X](https://twitter.com/KU_SPH_DPS) (formerly Twitter), posters and flyers, and the [OReC](https://orec.space/)—web platform dedicated to research recruitment.

Candidate participants were required to pre-register through a web form, based on whose responses we conducted an initial screening. The researcher (TN) then invited candidates who met the eligibility criteria to the baseline measurement via email, providing them with preliminary guidance through a document and a short video. Appointments for the measurement were scheduled through [TimeRex](https://timerex.net/), a web application designed for appointment adjustments. Candidates attended the baseline measurement at the scheduled time at the responsible research facility, where research staff verified their identity, measured their height and weight, calculated their BMI, and checked if it met the inclusion criteria (BMI between 23 and under 35). If the BMI criterion was met, the study explanation was provided once again, and informed consent was obtained through the signing of a consent document. The researcher TN was involved in baseline measurement, but not in the follow-up measurement at 12 weeks. Instead, the final assessment was measured by other volunteer research staffs who were blinded.

Participant recruitment took place from July 20, 2023 to October 10, 2023, upon achieving the target sample size. The follow-up measurement of all the participants ended on January 12, 2024.

## Ⅱ. Recruitment Flyer and Poster

The English version of the flyer (and poster) design was presented below (**Figure S1-1**), followed by an original Japanese version (**Figure S2-2**).

### Figure S1-1. English translated version of the recruitment poster.

### Figure S1-2. Original Japanese version of the recruitment poster.

## Ⅲ. Ethical Considerations

Written informed consent was obtained from all participants prior to their participation.

This study only recruited participants who were willing to participate, with adequate information provided regarding the rights and obligations associated with the study. Participants were free to withdraw from the study at any time. During blood collection at the measurements, to prepare for any sudden incidents such as vasovagal reactions due to self-puncture, a physician or nurse was available on-site. All research-related and participant data were securely stored in a locked safe within a locked room, accessible only with an ID card, in our laboratory of Department of Preventive Services, School of Public Health, Kyoto University. Participants received compensation upon completion of the study. Those who completed both the baseline and 12-week measurements were given an Amazon gift card valued at 5,000 yen. Additionally, at the end of the study, all control group participants who expressed interest were provided with the opportunity to engage in the compatible intervention program as those in the intervention group, which was also informed during the consent process.

## Ⅳ. Informed Consent Document

English-translated version of the informed consent document is presented below, followed by original Japanese version.

## 1. English version

A Brief Online Intermittent Fasting for weight loss:

A Randomized Pilot Study.

Informed Consent Document

I D：　　　　　　　　　　Date:　　　　　　　　　　　　:

1. **About**

The study “A Brief Online Intermittent Fasting for weight loss: A Randomized Pilot Study.” has been approved by the Kyoto University Graduate School and Faculty of Medicine, Ethics Committee, and is conducted under the permission of the dean.

1. **Research members and Institutions**

**・ Pincipal Investigator**

Taku Iwami (Overall Research Director):

Professor, Department of Social and Preventive Services, School of Public Health, Graduate School of Medicine, Kyoto University.

・ **Co-Investigators** at abovementioned institute

1. Tomonari Shimamoto (Research Operation, Planning, Writing): Specific Assistant Professor
2. Takashi Noda (Research Operation, Planning, Writing): Professional Degree Course
3. Norihiko Nishioka (Research Operation, Statistical Analysis): Specific Assistant Professor
4. Yukiko Tateyama (Research Operation): Specific Assistant Professor
5. Kosuke Kiyohara (Allocation): Visiting Researcher,
   and Associate Professor at Department of Food Science, Otsuma Women’s University

・ **Collaborators**

Mie Imanaka (Intervention Program and Content Creation): Professor, Department of Health and Nutrition, Faculty of Nursing and Nutrition, Shimane Prefectural University.

**・ Representatives of Collaborative Facility**

1. Chang-Zhen Li (Participant Recruitment): YAMATOKAI Kusaka Hospital
2. Daigo Goto (Participant Recruitment): Cold storage Japan Inc.
3. Yuzo Hoshino (Participant Recruitment): BUDDYTRAINING Co., Ltd.
4. **Purpose and Significance of the Study**

As you are aware, obesity and overweight are factors in numerous lifestyle diseases and constitute a global health problem. Recently, online weight loss programs that utilize smartphone apps and video conferencing have gained attention. We are developing cost-effective online weight loss programs that anyone can easily implement. This study will verify the effect of a "Brief Online Intermittent Fasting Program (hereinafter, Fasting Program)" using intermittent fasting, which has gained attention for weight loss and metabolic improvement. The program is designed to be straightforward compared to traditional weight loss programs, making continuous dietary restrictions easier to practice. If our research demonstrates the weight loss effect of the Fasting Program, it is expected to become a new method for lifestyle disease prevention. This study corresponds to a preliminary trial (pilot study) conducted prior to a larger main trial in the future. Based on the results obtained from this study, we will refine the program and research design to scientifically demonstrate the outcomes of the program in the next main trial with more participants.

1. **Methods and Schedules**

Participants will be randomly divided into two groups: those who will implement the Fasting Program (intervention group) and those who will not (control group), and the results will be compared after 12 weeks to verify the effect. Participants who consent to join the study will undergo physical measurements such as height and weight, blood pressure measurement, blood tests through finger pricking, and a lifestyle survey via a WEB questionnaire at Kyoto University or research cooperation institutions. Subsequently, researchers will randomly divide participants into the intervention and control groups, and you will participate in the study as assigned to your group. The intervention group will spend 12 weeks fasting once a week as usual. On fasting days, you will consume fasting meals provided by the research team. Additionally, on the first day of the trial period, a brief guidance on fasting practices of about 5 to 15 minutes will be conducted via a Zoom meeting. Furthermore, once a week, you will receive reminder messages through the smartphone app ‘Kenko-Nikki’ (hereinafter, research app) that include content related to fasting and a healthy lifestyle. Participants in the control group will not fast but will receive minimal care, receiving the same messages as the intervention group once a week (however, the content will be different from that of the intervention group and will not include information about fasting). During the trial period, participants in both groups will use the research app to record their self-measured weight and step count. Approximately 12 weeks after the end of the trial period (within a maximum of 14 weeks), physical measurements and blood tests, as well as a questionnaire survey, will be conducted again at Kyoto University or research cooperation institutions, and the results will be compared. After data collection from a total of 40 participants is completed, data analysis will begin. The entire study is scheduled to be completed by July 19, 2025.

**Participant's Schedule and Confirmation of Dates**

**※*Please note that the following items vary for each participant. Please reserve the dates for ①②③ in advance and confirm them with the staff atKyoto University during the initial measurement and final consent acquisition.**

1. **Initial Measurement Date and Time:______________**

Please use the schedule adjustment tool (TimeRex) provided to select a date to visit Kyoto University from the available dates on the calendar.

1. **Trial Start Date:__________ and Guidance Time: On that day at __________**

Regardless of whether you are assigned to the intervention group or the control group, the date you have reserved will be the start date of the trial. Please start recording your steps and weight in the research app by this date. If randomized to the intervention group, you will receive fasting guidance via a Zoom meeting at the reserved time ("Intervention Guidance Date and Time"). If you are in the control group, there will be no guidance via a Zoom meeting (the reserved date and time will be cancelled and released), but the trial will begin on this date. Please start using the research app to record your weight and steps, and receive weekly messages.

1. **Trial End Date:_________ and Final Measurement Time: _________**

The trial will end on the day of the final measurement, which is 12 weeks after the trial start date, and within the following 8 days (including the day 12 weeks after the start date). For example, if the trial start date is 2023/10/14 (Saturday), then 12 weeks later is 1/5 (Friday), so the final measurement dates are from 1/5 (Friday) to 1/12 (Friday). Please complete the final measurement within 14 days if delayed due to schedule changes or issues. Failure to do so will result in incompletion of the trial, and no compensation will be provided. Please be advised.

1. **Inclusion Criteria and Exclusion Criteria for Participation**

To investigate the effect of the online fasting program, we need the cooperation of 40 individuals for whom weight loss is medically recommended.
Specifically, the following conditions must be met for participation (**Inclusion criteria**):

- Aged 20 to 65 years
- BMI (Body Mass Index) between 23 and less than 35※Note: BMI = weight (kg) ÷ height (m) ÷ height (m). For example, if you weigh 70 kg and are 160 cm tall, your BMI = 70 ÷ 1.6 ÷ 1.6 = 27.34, which meets the criteria. In this study, the obesity criterion for Asians is set at a BMI of 23 or above, not 25.
- who can take at least one regular day off per week
- who can communicate smoothly in Japanese
- who have access to the internet and can use the research app without difficulty during the trial period
- who can come to Kyoto University or a research cooperation institution for measurements twice, before and after the study period (including fasting prior to measurement, finger pricking, and blood collection).

Individuals meeting any of the following criteria will not be eligible for participation (**exclusion criteria**):

- with a history of heart disease, kidney disease, mental illness, or other significant medical conditions
- taking medication for diabetes
- who habitually smoke or consume excessive amounts of alcohol (average of 4 or more drinks/day or 14 or more drinks/week)
- engaged in shift work, including night shifts
- who are expected to experience significant environmental changes, such as relocation or overseas travel, during the study period
- with eating disorders, food allergies, alcohol dependency, or those who require specific dietary therapy
- Pregnant or nursing , or women planning to become pregnant during the study period
- with a body fat percentage below 10% for men and 20% for women
- Athletes or sports competitors ( who practice or train for competitions or sports for more than 12 hours a week, belong to university sports clubs or corporate teams, etc.)
- Additionally, individuals deemed inappropriate for participation by the researchers may be excluded.

1. **Burden on Study Participants, Anticipated Risks and Benefits.**

Participants will cooperate with two measurements, before and after the fasting program period. Physical measurements, blood tests, and questionnaires will require up to one hour of your time. Transportation costs to Kyoto University for the measurements will be borne by the participants, and fasting before the physical measurements (breakfast) is required. Blood tests involve pricking your fingertip with a small needle. Although the amount of blood collected is minimal, a few tens of μL, it may cause slight pain. A doctor or nurse will be on standby in the same building in case of any unforeseen circumstances, such as a vasovagal reflex. Communication costs incurred during the installation and use of the research app "Health Diary" will be borne by the participants. For participants in the intervention group, there will be a time commitment and communication costs associated with the initial Zoom meeting for fasting guidance. The "intermittent fasting" practiced by participants in the intervention group has been implemented in numerous studies and its safety has been confirmed. Additionally, since a slight amount of energy and nutrients will be consumed on fasting days through fasting meals, there are no significant health risks to note. However, participants may experience hunger, fatigue, lethargy, and drowsiness on fasting days. If the nutritional status is poor to begin with, practicing the fasting program may exacerbate malnutrition or lead to a reduction in muscle mass and fat-free mass. Participants in the control group will only receive messages and record data using the research app during the trial period, but those who wish may receive the same program as the intervention group (including the provision of fasting meals) after the end of the study. Participants will be able to learn about their body composition, such as accurate weight, body fat percentage, and muscle balance, through physical measurements. Blood tests will reveal indicators such as hemoglobin A1c, blood triglycerides, and cholesterol, which can be useful for future lifestyle choices. The test results will be provided in print. Participants in the intervention group will receive fasting guidance, fasting meals, and supplements, and may experience weight loss and health improvement. Improvements in blood pressure, blood triglycerides, LDL cholesterol, and other indicators, as well as quality of life, are possible. Participants in the control group who wish to do so may receive the same program as the intervention group (including fasting meals) after the study ends. Therefore, participating in the study with the desire to implement the program will not be in vain if you are unable to do so.

1. **You can withdraw your consent at any time**

Even after deciding to participate, you can stop participating at any time. If you decide to stop participating, any data collected until that point will be destroyed. However, if the research results have already been published by the time you request to withdraw, it may not be possible to exclude your data. In the event of withdrawal, a consent withdrawal form will need to be completed, so please contact the research group [onlineifstudy@yobou.med.kyoto-u.ac.jp].

1. **There is no disadvantage to not consenting or withdrawing consent**

Please consider carefully before deciding whether or not to participate in the study. There will be no disadvantages if you choose not to consent or if you withdraw your consent.

1. **Information Disclosure**

This study is registered as a clinical intervention study with UMIN (University Hospital Medical Information Network) [https://center6.umin.ac.jp/cgi-open-bin/ctr/ctr_view.cgi?recptno=R000057445]. Results obtained from this study are planned to be presented at academic journals and conferences. Summaries of presentations and progress of the study will be posted on the website of the Division of Preventive Medicine, Department of Social and Preventive Services, Graduate School of Medicine, Kyoto University [https://yobou.med.kyoto-u.ac.jp].

1. **Viewing the Research Protocol**

If you wish to know more about the study, you can view materials related to the methods of the study within the limits that do not hinder the protection of other participants' personal information and the originality of the research. Please visit the special page of our laboratory HP [https://yobou.med.kyoto-u.ac.jp/onlineifstudy_pilot] or contact the research group.

1. **Handling of Personal Information**

Personal information such as "name, email address, address" will be collected from participants, along with telephone numbers, but we will take great care to protect this personal information. Data including personal information will be strictly managed in a lockable shelf in a lockable research laboratory at the Department of Preventive Services, Kyoto University. Addresses and telephone numbers will be deleted in an unrecoverable manner once they become unnecessary after randomization. After data collection from all 40 participants is completed, names and email addresses will be deleted, and analysis will be conducted using the research ID and each measurement data. Data will be sent to Dr. Kiyohara (affiliated with Otsuma Women's University) for the purpose of allocating participants to the intervention or control group, but only pseudonymized information (research ID, gender, age) will be sent, so personal information will not be disclosed. The results of the study are planned to be published in academic journals and presented at conferences, but personal information will not be used at that time.

After obtaining consent, it is possible to withdraw consent until the final day of the trial period, and upon withdrawal, all data except the reason for withdrawal will be deleted. However, data deletion requests will not be accepted after the consent withdrawal deadline has passed.

1. **Storage and Disposal of Information**

Data obtained from the study will be securely stored in a lockable shelf and all data including personal information will be deleted after the study ends. Data not containing personal information will be stored at the Department of Preventive Services, Graduate School of Medicine, Kyoto University for 10 years after the publication of research results, and will then be destroyed in an irreproducible manner. However, it is possible that the information collected in this study may be stored for a long period for future research that cannot be anticipated at the time of obtaining consent.

1. **Research Funding and Conflict of Interest**

This study is conducted with a grant for medical research in the field of preventive medicine from Ritsuan STC, Inc., but the donor's intentions do not influence the research. Ritsuan STC is an engineering human resource company and does not participate in the planning, analysis, or writing of the research. The app used in this research is provided at a low cost by Healthtech Laboratory, Inc. (HTK). Taku Iwami, the principal investigator, serves as an external director of HTK, and accepts other joint research. The researchers Tomonari Shimamoto and Yukiko Tateyama are employed with joint research funds from HTK and Kyoto University. Intellectual property transferred to the company by Taku Iwami and Tomonari Shimamoto is used in this research. The researcher Takashi Noda is a representative of Nihonsouken, LLC and operates a dietary guidance business including fasting. In conducting this research, conflicts of interest are managed in accordance with the "Kyoto University Conflict of Interest Policy" and the "Kyoto University Conflict of Interest Management Regulations," and are appropriately reviewed by the "Kyoto University Graduate School and Faculty of Medicine, Ethics Committee." Additionally, an auditor from the Audit Unit of the Department of Clinical Research Facilitation, Institute for Advancement of Clinical and Translational Science at Kyoto University Hospital, appointed by the principal investigator, conducts audits.

1. **Handling of Results Obtained from the Study**

Results from physical measurements and blood tests can be checked on the spot on the day of measurement, either on the screen or in print. If you do not wish to know the results, please let us know. These results will not be disclosed to anyone other than the measurer and researchers. Please note that these are research results and not intended for clinical use. The level of quality control required for clinical use is not institutionally conducted.

1. **Contacts**

If you have any questions or do not understand any terms explained, please feel free to contact us.

[Research Consultation Contact]

Department of Preventive Services, School of Public Health, Graduate School of Medicine, Kyoto University.
Address: Yoshida Konoe-cho, Sakyo-ku, Kyoto City, 606-8501
Contact: Tomonari Shimamoto, Takashi Noda
Phone Number: 075-753-2426
Email Address: [onlineifstudy@yobou.med.kyoto-u.ac.jp](mailto:onlineifstudy@yobou.med.kyoto-u.ac.jp)

[Kyoto University General Contact]
Research Promotion Section, General Affairs and Planning Division, Graduate School of Medicine, Kyoto University.
Phone Number: 075-753-9301
Email Address: 060kensui@mail2.adm.kyoto-u.ac.jp

1. **Financial Burden and Compensation**

The costs of downloading and using the app, communication for Zoom meetings and emails, as well as transportation costs for measurements at Kyoto University, will be borne by the participants. Upon completion of the final measurement, each participant will receive an Amazon gift voucher worth 5,000 yen as a token of appreciation.

1. **Use of Information in Future Research and Provision to Other Institutions.**

The information collected in this study may be used for future research that cannot be specified at the time of obtaining consent. Secondary use of the information and provision to other research institutions will be conducted only after approval by an ethics review committee based on a new research plan.

**If you fully understand the content of this study and agree to participate, please sign the document.**

## 2. Original Japanese version

簡易型オンライン間欠的断食プログラムの

減量効果を検証するランダム化パイロット研究

説明同意文書

参加者I D：　　　　　　　　　　説明日：　　　　年　　　　月　　　　日

1. **研究実施について**

「簡易型オンライン間欠的断食プログラムの減量効果を検証するランダム化パイロット研究」は京都大学大学院医学研究科・医学部及び医学部附属病院 医の倫理委員会の審査を受け、研究科長の許可を受けて実施しています。

1. **研究機関**

・ 研究責任者

石見 拓（研究の総括）:

京都大学大学院医学研究科 社会健康医学専攻 予防医療学分野 教授

・ 分担研究者

1. 島本 大也（研究の運営、企画立案、論文執筆）：同分野 特定助教
2. 野田 貴志（研究の運営、企画立案、論文執筆）：同分野 専門職学位課程
3. 西岡 典弘（研究の運営、統計解析）：同分野 特定助教
4. 立山 由紀子（研究の運営）：同分野 特定助教
5. 清原 康介（割り付け）：同分野 客員研究員及び大妻女子大学 家政学部食物学科管理栄養士専攻 准教授

・ 共同研究者

今中 美栄（介入プログラムとコンテンツ作成）：島根県立大学 看護栄養学部 健康栄養学科 教授

・ 研究協力機関

1. 李 昌珍（参加者の募集）：医療法人大和会 日下病院 代表
2. 後藤 大悟（参加者の募集）：コールドストレージ・ジャパン株式会社 代表
3. 星野 雄三（参加者の募集）：株式会社バディトレ 代表
4. **研究の目的および意義**

皆さんがご存知の通り、肥満や過体重は数々の生活習慣病の要因であり、世界的な健康問題の一つです。最近ではスマートフォンアプリやビデオ会議などを活用したオンラインでの減量プログラムに注目が集まっていて、私たちは誰もが簡単に実施できて費用対効果の高いオンライン減量プログラムの開発を行っています。

本研究は、体重減少や代謝の改善などで注目される間欠的ファスティング（断食）を活用した「簡易型オンライン間欠的断食プログラム（以下、ファスティングプログラム）」の効果を検証します。このプログラムは従来の減量プログラムと比較して指導内容が簡潔で、継続的な食事制限が容易に実践できるように設計されています。私たちの研究によってファスティングプログラムの減量効果が示されれば、生活習慣病対策の新たな方法として期待されます。

本研究は、将来のより規模の大きいメイン試験に先行して行う、予備的試験（パイロット試験）に相当します。本研究で得られた成果をもとにプログラムや研究デザインのブラッシュアップを行い、より多くの参加者を用いた次回のメイン試験により、プログラムの成果を科学的に実証していきます。

1. **研究方法およびスケジュール**

本研究では参加者の皆さんを、ファスティングプログラムを実施する方（介入グループ）、実施しない方（対照グループ）の2つのグループにランダムに分け、12週間後の結果を比較することで効果を検証します。

研究参加に同意していただいた方に対し、京都大学または研究協力機関にて身長・体重などの身体測定、血圧測定、指先の穿刺による血液検査、WEBアンケートによる生活調査を行います。その後、研究者が参加者を介入グループと対象グループにランダムに分け、割り当てられたグループとして研究にご参加いただきます。

介入グループには12週間の間、週に一度ファスティングをしながら通常通り過ごしていただきます。ファスティング日には、研究チームから提供されるファスティング食を摂取していただきます。また、試験期間の開始日に、Zoom会議にてファスティングの実践方法について5〜15分程度の簡単な指導を行います。さらに、週に一度、ファスティングや健康的なライフスタイルに関する内容を含むリマインドメッセージを、スマートフォンアプリ「健康日記」（以下、研究用アプリ）を介して受け取ります。

対照グループの方はファスティングは行いませんが、ミニマルケアとして週に一度、介入グループと同様のメッセージを受け取ります（ただし一部の内容が介入グループのそれとは異なり、ファスティングに関する内容は含まず別の内容になっています）。

試験期間はどちらのグループの方にも、研究用アプリを使用し自己測定の体重と歩数を記録していただきます。

試験期間終了となる約12週間後以降（最大14週間後以内）に、再度京都大学または研究協力機関で身体測定と血液検査、アンケート調査を行い、その結果の比較を行います。

総計40名からのデータ取得が完了した後、データの解析に移ります。

研究全体は、2025年7月19日までに終了する予定です。

**（要記入）参加者さまのスケジュールと日程の確認**

**※下記項目は、各参加者様によって異なります。必ず事前に①②③の日程をご予約の上、京都大学での初回測定および最終同意取得時にスタッフと共に確認してください。**

1. **初回測定日時：______月______日（　　）______時______分**

ご案内する日程調整ツール（TimeRex）を用いて、京都大学にお越しになれる日をカレンダーの空き日程からお選びください。

1. **試験開始日：______月______日（　　）および介入指導日時： 当日の_______時______分**

介入グループ、対照グループのいずれになった場合でも、ご予約いただいたこの日が試験の開始日となります。この日（まで）に研究用アプリでの歩数と体重の記録を始めてください。
ランダム化の結果、介入グループになった場合、上記ご予約時間（**「介入指導日時」**）に Zoom会議でのファスティング指導を受けていただきます。
一方、対象グループになった場合は、Zoom会議での指導は実施しません（ご予約いただいた日時はキャンセルになり、解放されます）が、試験はこの日に開始します。アプリでの体重・歩数の自己記録と、週次メッセージの配信が始まるので、忘れずに研究用アプリの使用を開始してください。

1. **試験終了日：______月______日（　　）および最終測定日時： 当日の______時______分**

試験開始日から12週間後以降の、最終測定の当日をもって試験が終了となります。**「最終測定」**は、試験開始日から12週間後以降の8日間の間にお願いします（12週間後の当日を含む）。

例として、2023/10/14（土）が試験開始日の場合、その12週間後が1/5（金）なので、その当日を含む1/5（金）から1/12（金）までの8日間が最終測定日になります。
予定変更やトラブルなどで遅れることがあっても、14日以内にお願いします。それができない場合は試験未完了となり、謝礼をお支払いすることができません。ご承知おきください。

1. **研究対象者の選定基準と除外基準**

オンラインで行うファスティングプログラムの効果を調べるために、健康上減量が勧められる方40名に協力していただく必要があります。具体的には、以下の項目を全て満たすことが参加の条件になります（**選定基準**）。

- 20歳以上65歳未満の方
- BMI（肥満指数）が23以上35未満の方
  ※注　BMI=体重÷身長÷身長。例えば体重70kgで身長160cmの方の場合、BMI=70÷1.6÷1.6=27.34であり基準を満たします。なお、この研究ではアジア人における肥満の基準として、BMI２５以上ではなく２３以上を基準としています。
- 週1日以上の規則的な休みが取れる方
- 日本語で円滑なコミュニケーションが取れる方
- インターネットを使用できる環境があり、試験期間中研究用アプリを不自由なく使用できる者
- 研究期間の前後2回、京都大学または研究協力機関での測定にお越しいただける方（測定直前までの絶食、自己穿刺による指先からの出血及び採血を含みます。）

以下に該当する方は参加の対象にはなりません（**除外基準**）。

- 心疾患や腎臓病、精神疾患、またはその他重大な医学的疾患の既往歴がある方
- 糖尿病治療薬を服用している方
- 喫煙や重度のアルコール飲用を習慣的に行っている方（平均4杯／日以上または14杯／週以上）
- 夜間労働を含む交代勤務に従事している方
- 研究期間中に転勤や海外旅行などの著しい環境の変化が予想される方
- 摂食障害や食物アレルギー、アルコール依存症など、特定の食事療法が必要と判断される方
- 授乳中や妊娠中の方。研究期間中に妊娠を予定している女性
- 体脂肪率男性10%以下、女性20%以下の痩せ型の方
- アスリートやスポーツ競技者に該当する方（1週間に12時間以上の競技やトレーニングの練習を行っている方、大学の運動部や実業団に所属し競技会などを目指している方など）

上記以外にも、研究者が不適当と判断した場合は参加の不対象とさせていただく場合があります。あらかじめご了承ください。

1. **研究参加者に生じる負担と、予想されるリスクおよび利益**

参加者の皆さんには、ファスティングプログラムを受ける期間の前後で２回の測定に協力していただきます。身体測定、血液検査、アンケートなどで最大1時間程度のお時間が必要です。測定のための京都大学への交通費は自費負担となり、身体測定前の（朝食の）断食も必要です。血液検査では、微小な針で指先を刺していただきます。採血量は数十μLと微量ですが、多少の痛みを伴います。瞑想神経反射などの万が一の事態に備えて、同じ建物内に医師または看護師が待機します。

研究用アプリ「健康日記」のインストール時や利用時に発生する通信費については、参加者の皆さんのご負担となります。

介入グループの方には、初回のZoom会議でのファスティング指導に際して時間的拘束と通信費が発生します。介入グループの方がファスティングプログラムで実践する「間欠的ファスティング」は、これまでの他の研究で幾度となく実施されており、安全性が確認されています。また、週に1日のファスティング日にはファスティング食によって若干のエネルギーと栄養素を摂取していただくので、特筆すべき健康リスクはありません。ただし、ファスティング日には空腹感や、疲労感、倦怠感、眠気などを感じる場合があります。また、普段の栄養状態が悪い場合には、ファスティングプログラムの実践により栄養不良を助長したり、筋量や除脂肪量などが減る可能性があります。

対照グループの参加者は、試験期間中は研究用アプリでのメッセージの受信と自己記録のみを行っていただきますが、研究終了後、ご希望の方は介入グループと同様のプログラム（ファスティング食の提供も含む）を受けることができます。

参加者の皆さんは身体測定において、正確な体重や体脂肪率、骨格筋のバランスなど体組成を知ることができます。また、血液検査でヘモグロビンA1cや血中中性脂肪・コレステロールなどの指標を知り、今後の生活に役立てることができます。検査結果は印刷物としてお渡しします。

介入グループになった方は、ファスティング指導を受け、ファスティング食とサプリメントを受け取ることができます。また、体重が減少し健康が増進するかもしれません。血圧や中性脂肪、LDLコレステロールなどの指標が改善し、生活の質も向上する可能性があります。

対象グループになった方も、ご希望の方は研究終了後に介入グループと同様のプログラム（ファスティング食の提供も含む）を受けることができます。なので、プログラムを実施したくて研究に参加したのに、それができずに無駄になる、ということはありません。

1. **いつでも同意の撤回ができます**

参加を決めた後も、いつでも参加をやめることができます。

参加をやめる場合は、それまでの調査記録は破棄します。ただし、お申し出があった時にすでに研究結果が公表されていたときなど、データから除けない場合もあります。参加をやめる場合には、同意撤回書を書いていただきますので、研究グループ［onlineifstudy@yobou.med.kyoto-u.ac.jp］までご連絡ください。

1. **研究に同意しない、または同意撤回において不利益はありません**

研究へ参加するかどうか、よくお考えのうえ、自由に決めてください。同意しない、もしくは同意を撤回された場合も、不利益な扱いを受けることは一切ありません。

1. **研究に関する情報公開について**

この研究は、臨床介入研究としてUMIN（大学病院医療情報ネットワーク）に登録しています[https://center6.umin.ac.jp/cgi-open-bin/ctr/ctr_view.cgi?recptno=R000057445]。また、この研究で得られた結果は、学術雑誌や学会での発表を予定しています。発表の概要や研究の進行状況は京都大学大学院医学研究科社会健康医学系専攻予防医療学分野のホームページ[https://yobou.med.kyoto-u.ac.jp]にて掲載いたします。

1. **研究計画書等の閲覧について**

研究について詳しく知りたい場合は、他の参加者の個人情報保護や研究の独創性に支障のない範囲で研究の方法に関する資料を見ることができます。当研究室の特設ページHP[https://yobou.med.kyoto-u.ac.jp/ onlineifstudy_pilot]をご覧いただくか、研究グループまでお問い合わせください。

1. **個人情報の取扱いについて**

参加者の皆さんから、個人情報として「氏名、メールアドレス、住所」を取得し、また、電話番号も取得しますが、これら個人情報の保護には十分配慮いたします。

個人情報を含むデータは京都大学医学部の施錠可能な研究室内の施錠された棚にて厳重に管理されます。住所と電話番号については、ランダム化を終えて不要になった時点で再現不能な形で削除します。計40名のデータの取得が全て終了した後、名前とメールアドレスを削除し、研究IDと各測定データを用いて、結果の解析を実施します。

参加者の皆さんが、介入グループになるか対照グループになるかの割り付けのために、分担研究者である清原氏（大妻女子大学にも所属）にデータを送りますが、その際は仮名化された一部の情報（研究ID、性別、年齢）を送りますので、個人情報が知られることはありません。

研究の結果は学術雑誌や学会発表で公表する予定ですが、この時にも個人の情報が使用されることはありません。

　参加同意の後も、試験期間最終日までは同意撤回が可能であり、同意撤回の際には同意撤回理由を除いた全てのデータを削除します。ただし、同意撤回期限を過ぎた後は、データ削除は受け付けられません。

1. **試料・情報の保管および廃棄の方法**

研究で得られたデータは施錠された棚にて厳重に保管され、研究終了後、個人情報を含むデータは全て削除します。個人情報を含まないデータは、京都大学大学院医学研究科予防医療学分野において、研究成果発表後から10年間保管され、その後再現不可能な形で破棄されます。

　ただし、のちに説明する「同意をうける時点では想定されない将来の研究」に使用するため、長期間にわたり保管する可能性があります。

1. **研究資金および利益相反について**

本研究は、株式会社リツアンSTCにうけた寄付金「予防医療学分野における医学研究助成」を用いて実施しますが、寄附者の意向が研究に影響することはありません。株式会社リツアンSTCはエンジニア派遣の会社であり、研究の計画、解析、論文執筆に一切関与しません。

本研究で用いるアプリは、株式会社ヘルステック研究所から安価にて提供されています。研究責任者である石見拓は、株式会社ヘルステック研究所の外部取締役に就任しているほか、他の共同研究を受け入れております。研究実施者の島本大也と立山由紀子は株式会社ヘルステック研究所と京都大学の共同研究費で雇用されています。また、石見拓と島本大也が企業に譲渡した知的財産を本研究で使用しています。研究実施者である野田貴志は、合同会社日本総合健康研究所の代表社員であり、ファスティングを含む食事指導事業を運営しています。

本研究の実施にあたり、利益相反については、「京都大学利益相反ポリシー」「京都大学利益相反マネジメント規程」に従い、「京都大学臨床研究利益相反審査委員会」において適切に審査しています。また、研究責任者が指名した京都大学医学部附属病院 先端医療研究開発機構 臨床研究支援部 監査ユニットの担当者が監査を実施します。

1. **研究より得られた結果の取り扱い**

身体測定や血液検査の結果は、測定当日のその場で画面や紙面上で確認することができます。測定結果を知りたくない場合にはお申し付けください。この結果は測定者と研究者以外の人に知られることはありません。

尚、これは研究の結果であり、診療の用に供する検査ではありません。診療の用に供する場合に求められる精度管理が制度的に行われているものではないことにご留意ください。

1. **相談の窓口**

説明の中でわからない言葉や質問がありましたら、何でも遠慮せずにお問い合わせください。

[本研究の相談窓口]

京都大学大学院医学研究科　社会健康医学専攻　予防医療学分野

住所： 〒606-8501京都市左京区吉田近衛町

担当者： 島本大也、野田貴志

電話番号： 075-753-2426

メールアドレス： onlineifstudy@yobou.med.kyoto-u.ac.jp

[京都大学の総合窓口]

京都大学医学研究科 総務企画課　研究推進掛

電話番号： 075-753-9301

メールアドレス： 060kensui@mail2.adm.kyoto-u.ac.jp

1. **経済的負担／謝礼について**

本研究で使用するアプリのダウンロードや使用、Zoom会議やメールのやり取りで発生する通信量、ならびに京都大学での測定にかかる交通費は参加者の皆さんのご負担となります。最後の測定を終えた皆さんにはおひとりにつき5000円分のAmazonギフト券を謝礼としてお支払いします。

1. **試料・情報の将来の研究における使用および他機関への提供**

本研究で収集した試料・情報は、同意を受ける時点では特定されない将来の研究のために用いる可能性があります。他の研究への二次利用および他研究機関へ提供する際は、新たな研究計画について倫理審査委員会で承認された後に行います。

**この研究の内容をよく理解し、参加に同意していただける方は、別途「研究参加の同意書」に署名していただきます。この説明文書は差し上げますので、よく読んでご検討ください。**
