# Supplementary material for: A Brief Web-Based and Mobile Intervention of Intermittent Fasting With Meal Support for Weight Loss Among Adults With Overweight and Obesity in Japan: Pilot Randomized Controlled Trial
Source: JMIR Mhealth Uhealth. 2026 Jan 26;14:e58930. doi: 10.2196/58930 (PMC12887555; doi:10.2196/58930)
Supplement: Multimedia Appendix 2 [file mhealth_v14i1e58930_app2.docx]

A Brief Web-Based Intervention of Intermittent Fasting with Meal Support for Weight Loss Among Overweight Japanese Adults:
A Pilot Study of a Randomized Controlled Trial

**Multimedia Appendix 2:**

**Supplementary Information on the Interventions**

This is a Multimedia Appendix to a full manuscript published in the J Med Internet Res. For full copyright and citation information see <http://dx.doi.org/10.2196/jmir.58930>

[Ⅰ. Intervention group—OIF 2](#_Toc160271459)

[1. Fasting meals delivery 2](#_Toc160271460)

[Table S2-1. Details of the fasting meals provided for the intervention group. 2](#_Toc160271461)

[2. Online fasting guidance 2](#_Toc160271462)

[Figure S2-1. The dates, number of participants, durations, and a part of asked questions of the online guidance sessions. 4](#_Toc160271463)

[Table S2-2. Details of asked questions of the online guidance sessions (zoom-in). 4](#_Toc160271464)

[3. App Messages Delivery. 6](#_Toc160271465)

[Table S2-3. The app messages sent to the intervention group. 6](#_Toc160271466)

[Ⅱ. Control group—minimal care 10](#_Toc160271467)

[Table S2-4. The app messages sent to the intervention group. 10](#_Toc160271468)

# Ⅰ. Intervention group—OIF

The ‘Brief Online Intermittent Fasting Program (OIF)’ consists of three distinct delivery components as outlined in the CONSORT-EHEALTH guideline: 1) a web-based component, 2) a non-web-based component and 3) a mobile component. These components were specifically:

1) fasting meals delivery,

2) online fasting guidance,

3) weekly message delivery via app.

Note that adhering to the guideline necessitates a longer but more descriptive title.

## Fasting meals delivery

Upon enrollment, all participants provided their names, addresses, and telephone numbers for the delivery process. After the allocation, the intervention group received fasting meals intended to replace one day’s meal. As mentioned in the manuscript, these fasting meals comprised six commercially available food items and a multi-vitamin-mineral supplement. **Table S2-1** provides information on food meals with summary of the total nutritional content. Further detailed information such as ingredients and nutritional facts are found in the linked website.

Participants were guided to undertake weekly fasting in accordance with the materials provided via email and an online guidance held via Zoom for a duration of 12 weeks, aiming for a total of 12 fasting practice. The prescribed definition of fasting communicated to participants was “consuming all provided fasting foods by 8 p.m. without any additional food intake.”

### Table S2-1. Details of the fasting meals provided for the intervention group.

| Food item | Weight (g) | Energy (kcal) | Protein (g) | Fat (g) | Carbohydrate (g) |
| --- | --- | --- | --- | --- | --- |
| [Tomato juice (KAGOME CO.,LTD., Nagoya)](https://www.kagome.co.jp/products/drink/A2404/) | 200 | 39 | 1.8 | 0 | 8.7 |
| [Fruit & Vegetable juice, original (KAGOME CO., LTD.)](https://www.kagome.co.jp/products/drink/A7257/) | 200 | 68 | 0.8 | 0 | 16.9 |
| [Fruit & Vegetable juice, berry salad(KAGOME CO., LTD.)](https://www.kagome.co.jp/products/drink/A7258/) | 200 | 80 | 0.8 | 0 | 19.6 |
| [Miso soup, low salt (Marukome Co.,Ltd., Nagano)](https://www.marukome.co.jp/product/detail/instant_070/) | 177 | 35 | 1.6 | 0.8 | 3.9 |
| [Miso soup, red dashi (Marukome Co.,Ltd., Nagano)](https://www.marukome.co.jp/product/detail/instant_071/) | 177 | 33 | 2 | 1 | 4.5 |
| [SOYJOY peanuts (Otsuka Pharmaceutical Co., Ltd., Tokyo)](https://www.otsuka-plus1.com/shop/g/g60953/) | 30 | 149 | 6.5 | 10.5 | 9.8 |
| [Multivitamin & mineral (Ajinomoto Co., Inc., Tokyo)](https://direct.ajinomoto.co.jp/supplement/multivitamin/) | 1 | 3 | 0 | 0 | 0 |
| Total | 985 | 407 | 13.5 | 12.3 | 63.4 |

## Online fasting guidance

For the online fasting guidance, participants were instructed to watch [a pre-recorded video](https://youtu.be/8H3Vgbt5Tic) (approximately 9 minutes) before attending a brief guidance session via Zoom. The procedures and session contents are detailed below.
During the informed consent process, participants scheduled the guidance session using [TimeRex](https://timerex.net/), recognizing this as the start date of the trial period, with both intervention and observation beginning at this point. Since neither the participant nor the research staff knew the participant’s allocation at the time of inclusion (consent and baseline measurement), all participants were asked to schedule their guidance session. Upon the allocation, those allocated to the intervention group attended the online guidance as they booked. For those allocated to the control group, the booking was canceled, but their trial period commenced on the same as they scheduled.

Participants were notified of their group via email within a week after the inclusion. The email to the intervention group included the URL for the pre-recorded guide video and a two-page leaflet as an attachment. Participants were advised to watch the video and read the leaflet before the online guidance session. These sessions were conducted either one-on-one or in small groups, with participants asked to enter the Zoom meeting muted and with their names anonymized, either by using a nickname or similar means. Initially, the intervention provider (TN) summarized the IF program in 2 to 3 minutes while displaying the program leaflet. Afterwards, participants were encouraged to ask questions. The session concluded when there were no further questions. **Figure S2-1** shows the overview information (dates, number of participants, durations, and asked questions [in Japanese]) on the held online guidance sessions, while **Table S2-2** shows the details of asked questions (zoom-up of Figure S2-1).
Despite the initial announcement that sessions could last between 5 to 10 minutes, up to a maximum of 30 minutes, no session exceeded 15 minutes, with an average duration of 3.5 minutes per participant, indicating the pre-sent materials were sufficiently informative.

### Figure S2-1. The dates, number of participants, durations, and a part of asked questions of the online guidance sessions.

### Table S2-2. Details of asked questions of the online guidance sessions (zoom-in).

| ID | Q1 | Answer to Q1 | Q2 | Answer to Q2 | Q3 | Answer to Q3 | Q4 | Answer to Q4 | Q5 | Answer to Q5 |
| --- | --- | --- | --- | --- | --- | --- | --- | --- | --- | --- |
| 678571 | ジュースは朝と昼と分けたほうがいいんですよね？ | そうですね、できるだけタイミングを分けたほうが空腹のリスクが少なくなります | ファスティング食は全部飲んだほうがいいですか？ | はい、できるだけお願いします。 | 野菜生活のオリジナルが苦手で | 苦手すぎて気持ち悪くなるくらいであれば結構ですが、飲める範囲で飲んでください。 |  |  |  |  |
| 800893 | ジムとか行っても大丈夫ですか？ | 軽運動であれば大丈夫です |  |  |  |  |  |  |  |  |
| 715333 | 特になし |  |  |  |  |  |  |  |  |  |
| 174669 | 一回のファスティングでそれぞれ１食ずつ食べればいいですか？ | はい、リーフレットが1回分ずつありますので、チェックを入れながら摂取してください。 | ファスティングは明日以降勝手に週一でやればいいですか？ | はい |  |  |  |  |  |  |
| 087863 | 緑茶2Lくらい毎日飲むんですけど水とかにしたほうがいいですか？ | プログラムのために無理して変更する必要はありませんが、一般的にはとりすぎかもしれません。ご自由にお願いいたします。 | 健康日記アプリは歩数と体重を記録する必要がありますか？ | 連携がうまくいっていれば、歩数は自動で取得されていると思いますので、毎朝体重を記録していただければ大丈夫です。 |  |  |  |  |  |  |
| 589860 | 普段のアプリの使用は体重だけですよね？ | はい | 味噌汁は2個ありますが、1日減塩と赤だしを一個ずつでいいんですよね？ | はい |  |  |  |  |  |  |
| 675792 | 体重測定のタイミンングはいつですか？ |  | ファスティングは1週間に一回ですが、土・日で交代で回しても良いのですか | 平均して週に一回であれば大丈夫です。先週忘れた分を次週に回すとかも大丈夫です |  |  |  |  |  |  |
| 614324 | 記録の仕方は、食べたものをメモっていけばいいのか？ | いえ、備考欄に入力する1.に関しては、「はい」で回答して構いません。 |  |  |  |  |  |  |  |  |
| 798378 | 健康日記のニックネームは参加者IDに変更した方がいいですか？そのままでもいいですか？ | そのままで大丈夫です | 記録するのは体重と歩数だけでいいのですか？ | はい、歩数は自動ですので体重だけ | 体重計に乗るタイミングは、毎朝食前・食後とか、決まった時間であればいつでもいいですか？ | はい、食事の量によっても変わるので、食前が良いかと | 二日連続ファスティングすることになってもいいですか？ | ルール上は大丈夫ですが、2日間は精神的負荷も大きくなるとは思います | ファスティング日は紅茶も飲んでいいのですか？ | はい、紅茶もカフェインを含むのでご留意ください |
| 896015 | 週に一回ということですが、水曜のあと月曜日、とかでもできますか？ | はい、大丈夫です。極論12週間の間12回実施すれば問題ありません | 6食ありますが、それぞれ1日の中で食べるおすすめのタイミングとかありますか？ | 1日3回のペースで好きなものを二食ずつ食べるか、1日6食と考えてお腹が空いた時にその場しのぎをする感じで食べるのが良いと思います。 | ・頭痛のときはロキソニンとかとっていいのか？ | 不快の軽減が優先ですのでそうしてください。ただ、塩を舐めるなど予防策は心がけてください | ・筋肉が減少するリスクというのは目安としてどのくらいありますか？ | 一般にこの程度であればそんな大きなリスクというのはありません。ただファスティング日に激しい筋トレをしても筋合成はしにくいので避けてください。筋肉の減少が気になるようでしたら、ファスティング日以外の日にしっかり運動して刺激を与えてください。 |  |  |
| 066459 | ファスティングは今週からですか？ | はい、これから1週間の間で一回やっていただければと思います。 |  |  |  |  |  |  |  |  |
| 156935 | 参加7人中6人。 14分。 |  |  |  |  |  |  |  |  |  |
| 394286 | 歩数が表示されなくなりました | アプリホーム画面の、体重とか血圧とか書いてあるボタンを押すと、表示項目が切り替わります。 |  |  |  |  |  |  |  |  |
| 538556 | トマトジュースが苦手で、他の飲んでもいいですか | どうしても苦手で気持ち悪いようであれば、それはスキップするか、他の何かを飲んでください。その旨記録していただければ、なんでも大丈夫です。 |  |  |  |  |  |  |  |  |
| 199224 | アプリの歩数の連携を間違っていいえにしちゃいました | 設定から、取得するを「はい」にしていただければ結構です。 |  |  |  |  |  |  |  |  |
| 305712 |  |  |  |  |  |  |  |  |  |  |
| 272586 |  |  |  |  |  |  |  |  |  |  |
| 638383 | なし |  |  |  |  |  |  |  |  |  |
| 559247 |  |  |  |  |  |  |  |  |  |  |
| 072917 |  |  |  |  |  |  |  |  |  |  |
| 713139 | 服薬状況は、メモした方がいいですか？ | そうですね、ご負担にならなければ、お願いします。 |  |  |  |  |  |  |  |  |
| 234834 |  |  |  |  |  |  |  |  |  |  |
| 420821 |  |  |  |  |  |  |  |  |  |  |
| 591259 | 前日の食事の時間について、決まりはありますか？ | 特にありません |  |  |  |  |  |  |  |  |
| 328369 | つぎの日は普通に食べていい？ | はい、ファスティング日以外は、特に制限はありません |  |  |  |  |  |  |  |  |
| 597943 |  |  |  |  |  |  |  |  |  |  |

## App Messages Delivery.

App messages were pre-programmed through a web administrative console. The first message was automatically dispatched at 8 a.m. on the first day of the trial period, with subsequent messages sent at 8 a.m. on the first day of the following weeks. Messages for the control group comprised general advice on a healthy lifestyle, focusing on diet, exercise, and sleep. For the intervention group, the messages were specifically tailored to fasting. For more detailed information, please refer to the message lists provided below.

All sent messages for the intervention group are displayed in **Table S2-3**. The number of the characters were calculated for the content in Japanese.

フォームの始まり

フォームの終わり

### Table S2-3. The app messages sent to the intervention group.

| Day | Number of Characters | Title | Content | English version |
| --- | --- | --- | --- | --- |
| 1 | 276 | ① はじめに | 今日から12週間の試験期間およびファスティングプログラムのスタートです！ まずは最初のファスティング日を決めて、手帳やカレンダーに記入しましょう。 当日はファスティング食のみの摂取になりますので、体力や気力ともに余裕のある日がいいでしょう。 忘れずに行えるように、曜日を固定してしまうのもお勧めです。 特に初めての場合は、脱力感や眠気などが生じることがありますから、運転や激しい身体活動のない日がいいですね。 様々な不安があると思いますが、プログラム通りに進めていただければ大丈夫です。 12週後の新しい自分に向けて、週に一度だけ頑張っていきましょう！ | ① Introduction Today marks the beginning of the 12-week trial period and the start of the fasting program! First, decide on your initial fasting day and mark it in your planner or calendar. On this day, you will only consume fasting meals, so it's best to choose a day when you feel both physically and mentally prepared. Setting a fixed day of the week can also help ensure you don't forget. Especially if it's your first time, you might experience feelings of lethargy or sleepiness, so it's advisable to choose a day without driving or intense physical activity. While you may have various concerns, rest assured that following the program as planned will be fine. Let's work hard just once a week, aiming for a new you in 12 weeks! |
| 8 | 325 | ② 眠気や空腹感を乗り切るには？ | プログラム期間２週目になりました！ 初めてのファスティングはいかがでしたか？ ちゃんとファスティング食を摂取した方は、心配していた空腹感もそれほど辛くはなかったのではないでしょうか？ お腹が空いて辛かった、という方も、次第に慣れてくると思われます。 空腹感には波があることが多いので、ちょうど強まってきた時に散歩するなどして気分転換を図るのがよいでしょう。 血糖値が低くなると、眠気や脱力感が生じやすくなりますから、こまめにファスティング食を補給することが、快適にファスティグ日を過ごすコツです。 また、カフェイン含有飲料には眠気や疲労感を和らげる作用もあるので、強い眠気やだるさが生じた場合には、緑茶やブラックコーヒー等を飲んで乗り過ごしましょう。 | ② How to Overcome Sleepiness and Hunger? We are now in the second week of the program! How was your first fasting experience? Those who properly consumed their fasting meals might have found the anticipated hunger not as challenging as expected. If you felt it was difficult due to hunger, it's likely that you will gradually get used to it. Hunger often comes in waves, so it's a good idea to take a walk or find other ways to distract yourself when it intensifies. As blood sugar levels drop, feelings of sleepiness and lethargy can arise, so replenishing with fasting meals frequently is key to comfortably getting through fasting days. Additionally, beverages containing caffeine can alleviate feelings of sleepiness and fatigue. If you experience intense drowsiness or sluggishness, drinking green tea or black coffee can help you get through it. |
| 15 | 383 | ③ ファスティング中の運動は？ | ３週目に突入しました！いつもアプリでの記録ありがとうございます。 アプリでは歩数の記録（自動）もお願いしていますが、これは運動量の参考にするためです。 ファスティング中は、無理のない程度の運動であれば行ってもらって構いません。 ただし、重いものを全力で持ち上げるような激しい筋トレや、長時間のランニングなど高強度な運動は安全のため控えた方がいいでしょう。 おすすめは、自重を使ったトレーニングや、室内でのジョギング、ヨガやストレッチなどの軽運動です。 無理のない運動を行うことで、ちょっとした気分転換にもなります。 また、ファスティング日以外の日にしっかりとトレーニングを行うことで、定期的なファスティングをしながらも筋肉量を維持・増量させることができます。 ですから、休む時（ファスティング中）は休む、動く時は動く、というメリハリをつけるのが、継続のコツですよ！ | ③ What About Exercise During Fasting? We've reached week three! Thank you for consistently logging in the app. While the app automatically records your steps, this is to serve as a reference for your level of physical activity. Moderate exercise during fasting is perfectly acceptable. However, intense workouts, such as heavy weightlifting or long-duration running, should be avoided for safety reasons. Recommended activities include bodyweight exercises, indoor jogging, yoga, or stretching, which are lighter forms of exercise. Engaging in manageable exercise can also serve as a nice change of pace. Moreover, by engaging in solid training on non-fasting days, you can maintain or even increase muscle mass while regularly fasting. Thus, resting on fasting days and being active on other days is key to sustainability. |
| 22 | 316 | ④ 普段の食事は変えなくていいの？ | いつも研究へのご協力、誠にありがとうございます。 ファスティング日以外の普段のお食事ですが、基本的に研究前と変えていただく必要はありません。 週に1日だけ食事が少なくなる分、栄養が不足するのではと心配されるかもしれませんが、最低限必要なカロリーと栄養素は、ファスティング食とサプリメントで十分補っていただけます。 心配な方は、普段から色とりどりの野菜や果物を多めに食べるようにしましょう。 カラフルな植物性食品には私たちの体調管理に必須のビタミンやミネラルが多く含まれている他、食物繊維も豊富ですので便秘予防にも役立ちます。 ファスティングを行っている分食事の量自体は減らさず、よく噛むことを意識して普段通り食べるようにしましょう。 | ④ Is It Okay Not to Change My Regular Diet? Thank you always for your cooperation with our research. Regarding your regular meals on non-fasting days, there is essentially no need to change your diet from what it was before the study. You might worry that reducing your meal intake one day a week could lead to nutritional deficiencies, but the fasting meals and supplements provided should sufficiently cover the minimum required calories and nutrients. For those concerned, it's a good idea to incorporate a variety of colorful vegetables and fruits into your regular diet. Colorful plant-based foods are rich in essential vitamins and minerals for managing our health, and they also provide plenty of dietary fiber, which helps prevent constipation. As you are fasting, there's no need to reduce the overall quantity of your meals. Focus on chewing your food well and continue eating as usual. |
| 29 | 289 | ⑤ いつもありがとうございます。 | 間欠的ファスティングプログラムも3分の１が経過し、５週目に入りました。 ファスティングを取り入れた日常に、そろそろ慣れてきた頃ではないでしょうか？ ファスティングを行った際にはアプリに忘れず内容を記録し、次の予定日も決めるようにしてくださいね。 また、毎日の体重記録と歩数の記録ができているかも、今一度チェックするようにしましょう。 皆さんの協力によって得られた結果は、今後の減量プログラムの改善や生活習慣病予防の研究に活用していきます。 残り２ヶ月ですが、引き続きご協力をお願い申し上げます。 プログラム終了後の最終測定で、元気な皆さんにお会いできることを楽しみにしています！ | ⑤ Thank you always. We have now passed one-third of the intermittent fasting program, entering the fifth week. You might be getting used to incorporating fasting into your daily routine by now. Please remember to record your fasting activities in the app each time you fast and to schedule your next fasting day as well. Also, take a moment to double-check whether you've been keeping up with daily records of your weight and steps. The results obtained with your cooperation will be used to improve future weight loss programs and research on the prevention of lifestyle-related diseases. With two months left, we kindly ask for your continued support. We look forward to seeing all of you, healthy and spirited, at the final measurement after the program concludes! |
| 36 | 372 | ⑥ 間欠的ファスティングって？ | 皆さんに行っていただいているファスティングは、一般に「間欠的ファスティング（または断続的断食）」というものです。 一定期間、定期的に短めのファスティングをすることを指します。 皆さんには週に1日行っていただいていますが、週に二日連続でファスティングしたり、1日おきにファスティングするといった、様々な方式があります。 ルールがシンプルで実現性が高いことから、減量のためのダイエット方法としても人気ですし、近年ではヒトを対象にした研究も行われています。 今回の研究では、ファスティングの簡易性と実行可能性に着目し、オンラインでの減量プログラムとして有効活用できないか、検証しています。 減量効果が出るか出ないかに関わらず、研究の質を高めるには、皆さんにしっかり最後の測定を終えていただくことが、何より大切です。 ぜひ最後までお付き合いくださいね。 | ⑥ What is Intermittent Fasting? The fasting you are participating in is commonly referred to as "intermittent fasting" (or intermittent dieting). It involves short periods of fasting on a regular, scheduled basis. While you are fasting one day a week in this program, there are various methods, such as fasting for two consecutive days a week or every other day. Due to its simple rules and high feasibility, it's a popular method for dieting and weight loss. In recent years, studies involving humans have also been conducted. In this research, we are exploring the simplicity and feasibility of fasting to see if it can be effectively utilized in an online weight loss program. Regardless of whether weight loss is achieved, completing the final measurement is crucial for enhancing the quality of the research. We sincerely hope you will stay with us until the end. |
| 43 | 450 | ⑦ ファスティングって何がいいの？ | ファスティングは減量目的のダイエットから、長寿に関わるヒトでの研究まで様々な場面で注目されていますが、何がそんなに良いのでしょうか？ 世間では、他のダイエットより痩せる、がんが治る、寿命が伸びる、など様々な噂が流れていますが、そのほとんどは微生物やマウスなどのモデル生物を使った実験から得られた示唆に過ぎません。 そのような結果はヒトに直接当てはめられるものではないので過度な期待は禁物です。 今のところ、ファスティングのメリットの多くは「全体的な摂取カロリーが少なくなること」でほとんど説明できると言われていて、「まとまった一定期間空腹でいること」それ自体の健康上のメリットは明らかになっていません。 しかし、少なくとも過体重の人にとっては、少食やカロリー制限自体が有益であることは確かなので、ファスティングはそれを容易にするためのシンプルなダイエット方法、と肯定的に捉えることもできます。今回の研究でも、そのようなファスティングの簡便性に注目しています。 引き続きご協力よろしくお願いいたします！ | ⑦ What’s So Good About Fasting? Fasting has garnered attention in various contexts, from weight loss diets to studies on longevity in humans, but what makes it so beneficial? There are many rumors circulating, such as it being more effective for weight loss than other diets, curing cancer, or extending lifespan. However, most of these claims are based on suggestions from experiments using model organisms like microbes and mice, and shouldn’t be directly applied to humans without caution. So far, many of the purported benefits of fasting can largely be explained by the overall reduction in calorie intake, and the health benefits of being hungry for a set period of time are not yet clear. However, for overweight individuals, the benefits of eating less or restricting calories are well-established, so fasting can be viewed positively as a simple diet method to facilitate this. Our research is focused on the simplicity of fasting. We appreciate your continued cooperation! |
| 50 | 464 | ⑧ 睡眠って大切！ | いつもご協力ありがとうございます。 もう少しで8週間が経過となり、プログラムも3分の2に達します。体調にお変わりはありませんか？ ファスティング日の実践指導では、「20時までに食事を終えましょう」とお伝えしましたが、これは皆さんの体調を整えるためです。 健康的には夜遅くに食べ過ぎないことが望ましいとされていますが、その理由として、睡眠の質が高まることが期待されています。 例えば深夜まで会食を楽しんだ次の日には、目覚めが良くなかったり睡眠不足を感じたりしますよね。 消化中の食べ物が少ない状態で就寝することで、体は余計なエネルギーを使わず回復に集中することができます。 また、夜中に食べてしまうことと比べると、体脂肪が燃焼しやすい状態も長く続きます。 皆さんの中には既に、ファスティング日の次の日は目覚めが良い、などの変化を感じている人もいるかもしれませんね。 試験終了後のアンケートでは皆さんから、プログラム全体を通した感想や意見などを、お聞きかせいただきますので、些細な気づきでもメモしておいていただけると大変嬉しいです！ | ⑧ Sleep is Important! Thank you for your ongoing cooperation. We are nearly at the 8-week mark, with the program reaching two-thirds completion. Have you noticed any changes in your health? In the fasting day guidance, we advised finishing meals by 8 PM to help regulate your body. It is generally recommended not to eat late at night for health reasons, as this is expected to improve sleep quality. For instance, you might have noticed feeling less refreshed or experiencing sleep deprivation the day after enjoying a late-night meal. Sleeping with less undigested food in your system allows your body to focus on recovery without spending extra energy, and it also prolongs the state in which body fat is more easily burned compared to eating late at night. Some of you might already have noticed feeling more refreshed the morning after fasting days. We look forward to hearing all your thoughts and observations about the entire program in the survey after the trial ends. Even minor insights are greatly appreciated, so please jot them down! |
| 57 | 448 | ⑨ お通じは良好ですか？ | ファスティング日はお通じが出ない、という方もいらっしゃるかもしれません。 食べたものがその人のうちに便となって出ていくわけではありませんが、物理的に消化管に食べ物が入ってくることが便意を催すきっかけになってはいるので、その機会が少ないファスティング中はどうしても排便が少なくなります。とはいえ1日出ないだけでは便秘ではありませんし、翌日以降にちゃんと出れば大丈夫なので安心してください。 普段から便秘がち、という方は、ぜひ食物繊維の摂取を意識してみましょう。特に、便を柔らかくすると言われる海藻類やキノコ類がおすすめです。 食物繊維は植物性食品に幅広く含まれていますが、意外と知られていないスーパースターが豆類やナッツ類です。 例えば茹で大豆は玄米の約6倍、白米の約20倍もの食物繊維が含まれています。これは今流行りの押し麦やもち麦と比べても多い水準です。 ナッツも糖質が少なく食物繊維が豊富なので、健康的なおやつとしておすすめです。ただし、脂肪やカロリーは高めなので食べ過ぎには注意しましょう！ | ⑨ How's Your Bowel Movement? Some of you might notice a lack of bowel movement on fasting days. While it's not the case that the food we eat directly turns into stool and exits the body, the physical entry of food into the digestive tract does trigger the urge to defecate, so it's natural for bowel movements to decrease during fasting due to less food intake. However, not having a bowel movement for a day does not necessarily mean constipation, and it's fine as long as you're able to go normally on subsequent days, so please don't worry.  For those who regularly experience constipation, consider increasing your intake of dietary fiber. Seaweeds and mushrooms, known to soften the stool, are particularly recommended. Dietary fiber is widely found in plant-based foods, but beans and nuts are somewhat unsung heroes in this regard. For instance, boiled soybeans contain about six times more dietary fiber than brown rice and about twenty times more than white rice, which is even higher than the currently popular barley varieties.  Nuts are also low in carbohydrates and rich in dietary fiber, making them a healthy snack option. However, be mindful of their high fat and calorie content and avoid overeating! |
| 64 | 616 | ⑩ ファスティングで糖尿病病予防？（今日は少し難しいハナシ） | 間欠的ファスティングは、糖尿病などの生活習慣病のリスクを下げるかもしれないといわれています。 その有望なメカニズムの一つに、代謝に関わるホルモンの調節があります。 間欠的ファスティングは肥満ホルモンである「レプチン」を減少させます。レプチンは脂肪細胞から分泌されるホルモンで、食欲を抑制する働きをもち、本来は安定的な体重の維持に関わっているとされます。しかし、肥満や糖尿病の方ではこのレプチンの効きが悪くなっており（レプチン抵抗性）、過剰に分泌されていることが生活習慣病の背景にあるとされています。 一方、ファスティングは別のホルモンである「グレリン」を増加させます。グレリンはレプチンに拮抗するホルモンで、空腹時に胃から分泌されます。 本来は食欲を増加させるホルモンですが、成長ホルモンを分泌させる働きがあり、これが少ないと元気と若さを保つことができません。やっぱり若さにはある程度の空腹の時間が必要なのかもしれません。 慢性的に肥満の方は血中レプチンレベルが高くグレリンのレベルが低いことが多く、ファスティングはこれらを正常なレベルに戻してくれるかもしれません。 また、ファスティングは「アディポネクチン」も増やすといわれています。このホルモンはイ​​ンスリンやβ細胞のはたらきを助け、動脈硬化を防ぐ働きがあります。 皆さんもプログラム終了後の測定で、以前よりもいい検査結果が出るかもしれません。ぜひ最後までお付き合いください！ | ⑩ Fasting for Diabetes Prevention? (A Bit of a Complex Topic Today) Intermittent fasting is said to potentially reduce the risk of lifestyle diseases such as diabetes. One promising mechanism involves the regulation of metabolism-related hormones. Intermittent fasting decreases the "leptin" hormone, which is associated with obesity. Leptin is secreted by fat cells and has an appetite-suppressing effect, traditionally playing a role in maintaining stable body weight. However, in individuals with obesity or diabetes, leptin's effectiveness is diminished (leptin resistance), and its overproduction is believed to contribute to the development of lifestyle diseases. Conversely, fasting increases another hormone, "ghrelin," which opposes leptin and is secreted by the stomach when hungry. While ghrelin typically increases appetite, it also stimulates the secretion of growth hormone, essential for maintaining vitality and youth. Perhaps a certain degree of hunger is necessary for youthfulness. Chronically obese individuals often have high blood leptin levels and low ghrelin levels, and fasting might help restore these to normal levels. Fasting is also said to increase "adiponectin," a hormone that aids the function of insulin and β-cells and prevents arteriosclerosis. Following the program, you might find improved test results compared to before. Please stay with us until the end! |
| 71 | 330 | ⑪ あともう少し！ | ついに11週目に突入です！ここまでよく頑張りました！ 順調であれば、ファスティングも残すところあと2回ですね。 人によってはもう少し回数が残っていたり、既に12日分やってしまった方？もいるかもしれません。 今回のプログラムでは週に1日のファスティングが原則でしたが、12週間の間に12日分が自主的にできれば条件的にはクリアです！ なので順調に進まなかった方も諦めず、最後に調整していただいても構いません。 また、（例えば）8回分しかできていないよ…という方でも大丈夫！ どんな達成度であれ、最後の測定にお越しいただけることが何より大切で、研究の遂行にとってありがたいことです。 ぜひ最後までお付き合いください！（最終測定の予約日は今一度、ご確認くださいませ。） | ⑪ Just a Little More! We've finally entered week 11! You've done an excellent job so far! If all goes well, you only have two more fasting days left. Some of you might have a few more sessions to go, or perhaps some have already completed the 12 days. The principle of the program was to fast one day a week, but as long as you've managed to complete 12 days of fasting over the 12 weeks, you've met the criteria! So, for those who haven't progressed as smoothly, don't give up; it's okay to make adjustments towards the end. And for those who have only managed to fast for, say, 8 days, that's fine too! No matter the level of completion, what matters most is that you attend the final measurement. Your participation is greatly appreciated and vital for the research. Please stay with us until the end! (Please re-check the reservation date for your final measurement.) |
| 78 | 308 | ⑫ おめでとうございます！ | ついにプログラム最後の週です、お疲れ様でした！ お忙しい中、研究に協力していただき、誠にありがとうございました。 協力者の皆さんから様々なデータをお借りすることで、医学の発展に貢献することできます。 ぜひ最後までお付き合いください。 最終測定がまだ決まっていない、予定に変更がある方は、早めに私たち研究チームまでご連絡ください。 最終測定では、WEBフォームでのアンケート調査があります。皆様の体調に関してお聞きするほか、プログラム全体を通した感想・意見・気づき等を自由記載でお聞きいたしますので、これまでの経過を振り返っておいてください。 ご協力本当にありがとうございました。あと1週間、よろしくお願いいたします！ | ⑫ Congratulations! We have finally reached the last week of the program. Well done, and thank you for your hard work! We sincerely appreciate your cooperation with our study amidst your busy schedule. By borrowing various data from all our participants, we can contribute to the advancement of medicine. We hope you will stay with us until the very end. If you haven't yet decided on a date for your final measurement or need to make changes to your schedule, please contact our research team as soon as possible. The final measurement will include a survey conducted via a web form. We will inquire about your health status and ask for your overall impressions, opinions, and insights about the entire program, so please take a moment to reflect on your experience thus far. Thank you so much for your cooperation. We look forward to your continued participation for one more week! |

# Ⅱ. Control group—minimal care

Participants in the control group only received the weekly app messages.

All sent messages for the control group ap are displayed in **Table S2-4**. The number of the characters were calculated for the content in Japanese.

### Table S2-4. The app messages sent to the intervention group.

| Day | Number of characters | Title | Content | English version |
| --- | --- | --- | --- | --- |
| 1 | 185 | ① はじめに | 今日から12週間の試験期間がスタートです！ 歩数と体重は毎日記録を録っていただきます。 歩数はヘルスヘアアプリやGoogle Fitとの連携で取得し、 体重は毎朝体重計に乗り、当日の体重をアプリに記入してください。 また、週に一度のペースでお知らせが届きますので、アプリのホーム画面右上のメールアイコンから確認してください。 それでは、12週間よろしくお願いいたします。 | ① Introduction Today marks the start of the 12-week trial period! You will be recording your daily step count and weight. Steps will be collected through integration with health care apps like HealthKit or Google Fit, and you are requested to weigh yourself every morning and enter your weight for the day in the app. Additionally, you will receive notifications on a weekly basis, so please check them by clicking on the mail icon at the top right corner of the app's home screen. We look forward to your participation over the next 12 weeks. |
| 8 | 400 | ② ぜいたくランチにご注意を！ | 試験期間２週目になりました！ 突然ですが、お昼ご飯、食べ過ぎていませんか？ お昼休憩に飲食店でランチする方も多いと思いますが、お腹いっぱいまでたくさん食べると、血糖値が急上昇し、消化・吸収のために休息を得ようとする体の応答が起き、血糖値が下がるタイミングで眠くなってしまいます。また、急激に血糖値が上下することで、より太りやすくなるとも言われています。 午後のお仕事や勉学に集中して取り組むためにも、ほどほどの量で済ませたいものです。 特に白ごはんやパン、パスタなど、糖質ばかりの食事にならないようにしましょう。 忙しい時はおにぎりや市販のパンの個食になりがちですから、要注意ですね。 サラダなどの野菜から先に食べる、脂質やタンパク質をバランスよく含む食事にする、よく噛んでゆっくり食べる、などの工夫で、血糖値の急激な上昇と降下を防ぐことができます。 快適な生活のためにも、ぜひ心掛けてみてください！ | ② Beware of Luxurious Lunches! We are now in the second week of the trial period! Suddenly, have you been overeating at lunch? Many people dine out during lunch breaks, but eating until you're full can cause a rapid spike in blood sugar levels, prompting your body to seek rest for digestion and absorption, which in turn can make you feel sleepy as your blood sugar level drops. Additionally, rapid fluctuations in blood sugar levels are said to make it easier to gain weight. To stay focused on your work or studies in the afternoon, it's best to eat in moderation. Especially avoid meals dominated by carbohydrates, such as white rice, bread, and pasta. Be cautious, as it's easy to resort to single servings of rice balls or store-bought bread when busy. Starting with vegetables like salads, having balanced meals with fats and proteins, and eating slowly by chewing well can help prevent sharp spikes and drops in blood sugar levels. For a more comfortable life, please keep this in mind! |
| 15 | 465 | ③ 運動して体力をつけるコツ！ | ３週目に突入しました！いつもアプリでの記録ありがとうございます。 アプリでは歩数の記録（自動）もお願いしていますが、これは運動量の参考にするためです。 試験期間は、それ以前と変わらない程度であれば、もちろん運動していただいて構いません。 適度な運動は、健康的な毎日に欠かせない習慣です。 運動をして体力をつけるためのポイントは、「負荷」と「休息」のメリハリをつけることです。 専門的には「過負荷の原理」と言いますが、自分の持つ体力以上の負荷を与えなければ、体力は向上しません。なので、ウォーキングよりもジョギングの方、ジョギングよりランニングの方が、一般的にトレーニング効果は高いのです。安全に行える範囲ならば、軽運動を長く続けるよりも中強度の運動を短時間しっかりやる方が、体力作りにとっては良いということになります（脂肪燃焼など、目的によっては逆になります）。 そして、もっと重要なのが「休息」。体力は運動する時にではなく、休んでいる時に向上します。 やるならやるでしっかり動き、疲れたらしっかり休む。これが体力作りの鉄則です。 | ③ Tips for Building Physical Strength through Exercise! We've reached week three! Thank you for consistently logging in the app. While the app automatically records your steps, this is meant to serve as a reference for your level of physical activity. During the trial period, you are of course welcome to continue exercising as you did before, as long as it's at a similar level. Moderate exercise is an essential habit for a healthy daily life. The key to building physical strength through exercise is to balance "load" and "rest." This is professionally known as the "principle of overload," meaning that without applying a load greater than what your body is accustomed to, your physical fitness will not improve. Therefore, jogging has a generally higher training effect than walking, and running more so than jogging. If safe to do so, doing moderate to intense exercise for shorter durations is better for building physical fitness than long periods of light exercise (though this may vary depending on goals, such as fat burning). What's even more important is "rest." Physical strength improves not while exercising, but during rest periods. If you're going to exercise, do it vigorously, and when tired, rest thoroughly. This is the golden rule for building physical strength. |
| 22 | 339 | ④ 食事は何も変えなくていいの？ | いつも研究へのご協力誠にありがとうございます。 普段のお食事ですが、基本的に研究前と変えていただく必要はありません。 とはいえ健康には気をつけたいもの。外食やコンビニ食が多くなりがちな方は、色とりどりの野菜や果物を食べるように心がけましょう。 カラフルな植物性食品には私たちの体調管理に必須のビタミンやミネラルが多く含まれている他、食物繊維も豊富ですので便秘予防にも役立ちます。 また、このような未加工の生鮮食品は、総じてよく噛まないと食べられないものが多いので、咀嚼回数が増えます。たったそれだけでも、満腹感を増やすことで食べ過ぎを防ぎ、消化と吸収の負担を和らげることができます。 寒暖差が大きく体調を崩しやすいこの季節、栄養のあるものをしっかり食べて体を整えてくださいね！ | ④ Do I Need to Change My Diet? Thank you always for your cooperation with our research. Regarding your usual diet, there is essentially no need to change anything from before the study began. However, it's important to pay attention to your health. If you frequently eat out or rely on convenience store meals, try to incorporate a variety of colorful vegetables and fruits into your diet. Colorful plant-based foods are rich in vitamins and minerals essential for managing our health and also provide plenty of dietary fiber, which can help prevent constipation. Additionally, these unprocessed, fresh foods generally require more chewing, which can increase the number of chews per bite. This simple change can enhance feelings of fullness, preventing overeating and easing the burden of digestion and absorption. During this season, with its significant temperature variations that can easily affect your health, make sure to eat nutritious foods to keep your body in good condition! |
| 29 | 186.00 | ⑤ いつもありがとうございます。 | 試験期間も３分の１が経過し、５週目に入りました。 毎日の体重測定とアプリへの記録にも、そろそろ慣れてきた頃ではないでしょうか？ 皆さんの協力によって得られた結果は、今後の減量プログラムの改善や生活習慣病予防の研究に活用していきます。 残り２ヶ月ですが、引き続きご協力をお願い申し上げます。 プログラム終了後の最終測定で、元気な皆さんにお会いできることを楽しみにしています！ | ⑤ Thank you always. We've now passed one-third of the trial period and have entered week five. By now, you might be getting used to daily weight measurements and recording them in the app. The results obtained from your cooperation will be utilized for future improvements in weight loss programs and research on the prevention of lifestyle diseases. With two more months to go, we kindly ask for your continued support. We're looking forward to seeing all of you healthy and well at the final measurement after the program ends! |
| 36 | 387 | ⑥ 「食事バランスガイド」を知っていますか？ | 厚生労働省と農林水産省が定めた「食事バランスガイド」は、特別な病気をお持ちでない方々の健康維持を目的に、食事の望ましい組み合わせとおおよその量をイラストでわかりやすく示したもので、きっと皆さんも一度は目にしたことがあるでしょう。 健康的なライフスタイルを、水・お茶の摂取を軸に、運動によって回転するコマに例えたこのイラストによれば、主食はごはん４杯程度、副菜（野菜、きのこ、いも、海藻料理）は５皿程度、主菜（肉、魚、卵、大豆料理）は３皿程度、乳製品は牛乳１本程度、果物２個程度を食べるのが良いとされています。 皆さんの普段のお食事はどうでしょう。このバランスを守れていますか？ このイラストの内容を意識して食べるだけで、適正体重の維持、必要な栄養素の充足、生活習慣病の予防にも役立ちます。 栄養バランスが気になるとき、明日の献立に迷ったときには、ぜひ活用してみてください！ | ⑥ Are you familiar with the "Food Balance Guide"? The "Food Balance Guide" established by the Ministry of Health, Labour and Welfare and the Ministry of Agriculture, Forestry and Fisheries is designed to help maintain the health of individuals without special medical conditions. It clearly illustrates the ideal combination and approximate amounts of food using illustrations, and you've likely seen it at least once. This guide, which compares a healthy lifestyle to a spinning top powered by exercise and centered on the intake of water and tea, suggests that a good diet consists of about four bowls of rice for the main dish, five plates of side dishes (vegetables, mushrooms, potatoes, seaweed dishes), three plates of main dishes (meat, fish, eggs, soy dishes), about one bottle of dairy products, and roughly two pieces of fruit.  How does your daily diet stack up? Are you maintaining this balance? Just being mindful of the guidance in this illustration can help maintain a healthy weight, fulfill the necessary nutrient intake, and prevent lifestyle-related diseases. When you're concerned about nutritional balance or wondering what to prepare for tomorrow's menu, consider using this guide as a reference! |
| 43 | 580 | ⑦ 怪しい健康情報にご注意を！ | テレビやSNSでは毎日のように、健康食品やサプリメントの宣伝が流れてきますが、中には不正確な情報や、誇大な表現が含まれているものも少なくはありません。 これを飲めば痩せる、頭皮にかければ髪が生える、がん細胞が死滅する等、いろんな噂は絶えませんが、そのような効果は、あくまで個人がたまたま体験した幸運だったり、マウスなどの実験動物において認められた程度のものだったりすることがほとんどです。 そのような限られた証拠だけでは、人間一般に直接当てはめられるものではないので、過度な期待は禁物です。 特に、「こんなすごい有名人も、このサプリを毎日飲んでます！」と言った論調には要注意。その方は高額なサプリを飲んでいるから健康なのではなく、もともと健康でよく働くから十分な収入があり、高いサプリを買えているだけなのかもしれません。 マッチョな人が毎日キュウリを食べているからといって、きゅうりが筋肉を増やす！と考える人はいませんよね？それと同じことです（笑）。 今のところ、これだけ飲めばみんな体調が良くなる！、たくさん食べて誰でも簡単に痩せる！ことが期待できそうなスーパーフードや食事法は見つかっていません。 健康的な食生活を、規則正しくバランスよく、という原則に従うのが一番で、そのための環境づくりに時間やコストをかけていくのが、王道かつ健康への近道といえそうです。 | ⑦ Beware of Dubious Health Information! Every day, TV and social media are flooded with advertisements for health foods and supplements, many of which contain inaccurate information or exaggerated claims. Claims like "Drink this and lose weight," "Apply this to your scalp and hair will grow," or "This kills cancer cells," are endless. However, such effects are often just fortunate personal experiences or observed in experimental animals like mice. Such limited evidence cannot be directly applied to humans in general, so it's important not to have unrealistic expectations. Be particularly cautious of narratives claiming, "Even this famous person drinks this supplement daily!" Their health likely isn't due to expensive supplements but rather because they are inherently healthy and earn enough to afford such luxuries. Just because a muscular person eats cucumbers daily doesn't mean cucumbers boost muscle growth, right? It's the same principle (laughs). So far, there are no superfoods or diets that promise improved health for everyone or allow anyone to lose weight easily by eating plenty. The best approach remains to follow the principle of a healthy diet: eating regularly and maintaining a balanced diet. Investing time and resources in creating an environment that supports such a lifestyle is the most reliable and direct path to health. |
| 50 | 438 | ⑧ 睡眠って大切！ | いつもご協力ありがとうございます。 もう少しで8週間が経過となり、プログラムも3分の2に達します。 ところで、夜遅くに食べすぎないほうがいいって、聞いたことありませんか？ 理由はいくつかありますが、その一つとして、深夜の食事は睡眠の質に悪影響を与えるのではないか、ということが言われています。 例えば、夜遅くまで会食を楽しんだ次の日には、目覚めが良くなかったり、睡眠不足を感じたりしますよね。 皆さんが寝ている間も臓器は働いています。消化中の食べ物が多い状態で眠ると、体は余計なエネルギーを使ってしまい、回復に集中することができないのです。 睡眠の質が低いと、満腹中枢がうまく働かなかったり、運動のやる気も出ないので、そのことが肥満と関連しているのかもしれません。 また、睡眠に与える影響の他にも、日中よりも夜に多く食べる方がそもそも体脂肪がつきやすいとも言われています。  できるだけ良い睡眠が取れるように、早めの「晩ごはん」（夜ごはんではなく）を心がけた方が良いようです。 | ⑧ Sleep is Important! Thank you always for your cooperation. We're nearing the 8-week mark, which means the program is two-thirds complete. Have you ever heard that it's better not to eat too late at night? There are several reasons for this, one being that eating late can negatively affect the quality of your sleep. For instance, you might have noticed feeling less refreshed or experiencing sleep deprivation the day after enjoying a late-night meal. Your organs are still working while you sleep. Sleeping with a lot of undigested food means your body uses extra energy, preventing it from focusing on recovery. Poor sleep quality can impair the function of the fullness center in the brain and reduce motivation for exercise, which could be linked to obesity. Additionally, eating more at night than during the day is said to increase body fat more easily. To ensure better sleep, it seems advisable to aim for an early "dinner" (not late night meal). |
| 57 | 371 | ⑨ お通じは良好ですか？ | 毎日スッキリと過ごしたいものですよね。 今日は普段から便秘がち、という方に、ちょっとしたアドバイスを。 ぜひ日頃から、食物繊維の摂取を意識してみましょう。 食物繊維といっても、食物繊維が添加されているジュースや加工食品ではダメで、自然食品から摂取するのがポイントです。 特に、便を柔らかくするといわれる水溶性食物繊維を豊富に含む、海藻類やキノコ類がおすすめです。 食物繊維は植物性食品に幅広く含まれていますが、意外と知られていないスーパースターが豆類やナッツ類です。 例えば茹で大豆は玄米の約6倍、白米の約20倍もの食物繊維が含まれています。 これは今流行りの押し麦やもち麦と比べても多い水準です。 ナッツも糖質が少なく食物繊維が豊富なので、健康的なおやつとしておすすめです。 ただし、脂肪やカロリーは高めなので食べ過ぎには注意しましょう。 | ⑨ How's Your Bowel Movement? Everyone wants to feel refreshed every day. Today, I have some advice for those who often experience constipation. Make a conscious effort to incorporate dietary fiber into your diet daily. It's important to note that dietary fiber from juices or processed foods with added fiber isn't the best choice; the key is to get it from natural foods. Specifically, seaweeds and mushrooms, which are rich in water-soluble dietary fibers known to soften stool, are highly recommended. Dietary fiber is widely found in plant-based foods, but legumes and nuts are the unsung heroes. For example, boiled soybeans contain about six times the dietary fiber of brown rice and about twenty times that of white rice, which is even more than what's found in trendy grains like barley or millet. Nuts are also low in carbohydrates and rich in dietary fiber, making them a healthy snack option. However, be mindful of their high fat and calorie content and avoid overeating. |
| 64 | 697 | ⑩ 生活習慣病とホルモンのお話（今日は少し難しいハナシ） | 現代型の食生活をしていると、糖尿病などの生活習慣病のリスクが高まります。 そのメカニズムの一つとして、代謝に関わるホルモンの調節があります。 みなさんは、肥満ホルモンともいわれる「レプチン」という、脂肪細胞から分泌されるホルモンをご存知ですか？ 肥満ホルモンと聞くと、悪い存在のように聞こえますが、食欲を抑制する働きを持っています。脂肪細胞が増える（太る）と、そこから分泌されるレプチンも増えるので、食欲が抑えられて、結果的に安定的な体重を維持することに役立ちます。 しかし、肥満や糖尿病の方ではこのレプチンの効きが悪くなっており（レプチン抵抗性）、過剰に分泌されているのにもかかわらず効いていない（さらに食べ過ぎて太ってしまう）状態にあるそうです。これは、２型糖尿病におけるインスリン抵抗性と一緒ですね。 このことが、慢性疾患である生活習慣病の背景にあるとされています。  一方、空腹時に胃から分泌されるホルモンとして、グレリンがあります。 グレリンは食欲を増加させる機能がありますが、レプチンと拮抗するホルモンであり、バランスよく働くことが求められます。 なんとグレリンは成長ホルモンを分泌させる働きがあり、これによって骨や筋肉や肌を健康に保ち、動脈硬化や生活習慣病も予防してくれます。 やっぱり元気と若さを保つには、ある程度の空腹の時間が必要なのかもしれません。 また、睡眠時間が減るとレプチンとグレリンのバランスが崩れ、食欲過剰になってしまうこともわかっています。 小さい子供を見習って、お腹が空くまで外で元気に動き、しっかり寝て休む、というメリハリのある生活が大事だといえるでしょう。 | ⑩ The Relationship Between Lifestyle Diseases and Hormones (A Bit Complex Today) Adopting a modern diet increases the risk of lifestyle diseases like diabetes. One of the mechanisms behind this involves the regulation of hormones related to metabolism. Are you familiar with "leptin," often referred to as the obesity hormone, which is secreted by fat cells? Despite sounding negative, leptin actually suppresses appetite. As fat cells increase (as one gains weight), the amount of leptin secreted also increases, which in turn helps suppress appetite and ultimately aids in maintaining a stable weight. However, in individuals with obesity or diabetes, the effectiveness of leptin decreases (known as leptin resistance), and despite its excessive secretion, it doesn't function properly, leading to overeating and further weight gain. This situation is similar to insulin resistance seen in type 2 diabetes. This phenomenon is considered to be a background factor in chronic diseases like lifestyle diseases. On the other hand, there's "ghrelin," a hormone secreted by the stomach when hungry. Ghrelin increases appetite but acts in opposition to leptin, and a balance between the two is necessary. Interestingly, ghrelin also stimulates the secretion of growth hormone, which helps maintain the health of bones, muscles, and skin, and also prevents arteriosclerosis and lifestyle diseases. It seems that maintaining some level of hunger might indeed be necessary to preserve vitality and youth. Furthermore, it's known that reduced sleep disrupts the balance between leptin and ghrelin, leading to excessive hunger. Mimicking the habits of young children—actively moving around until feeling hungry and then getting plenty of rest—could be essential for a balanced lifestyle. |
| 71 | 222 | ⑪ あともう少しです！ | 試験期間も残すところ約2週間となりました。 寒い日が続いておりますが、体調はお変わりありませんか？ 忙しいこの季節。インフルエンザなどの感染症や、ノロウイルスなどの食中毒も増え、体調を崩しがちです。 手洗い・うがいの実践、保温や加湿、栄養のある食事と十分な睡眠を心がけ、しっかりとした体調管理で師走を走り切りましょう。  また元気な姿で、近々みなさんにお会いできることを楽しみにしております！ （最終測定の予約日は今一度、ご確認くださいませ。） | ⑪ Just a Little More! There are about two weeks left in the trial period. The days are getting colder, but I hope your health remains stable. During this busy season, the risk of infections like the flu and food poisoning, including norovirus, increases, making it easier to fall ill. Practice handwashing and gargling, ensure proper warmth and humidity, eat nutritious meals, and get plenty of sleep to maintain your health through the end of the year. I look forward to seeing all of you healthy and well soon! (Please double-check the date of your final measurement appointment.) |
| 78 | 327 | ⑫ おめでとうございます！ | ついに試験期間最後の週です、お疲れ様でした！ お忙しい中、毎日の測定は大変だったかと思いますが、協力していただき誠にありがとうございました。 協力者の皆さんから様々なデータをお借りすることで、医学の発展に貢献することできます。 ぜひ最後までお付き合いください。 最終測定がまだ決まっていない、予定に変更がある方は、早めに私たち研究チームまでご連絡ください。 最終測定では、WEBフォームでのアンケート調査があります。皆様の体調に関してお聞きするほか、プログラム全体を通した感想・意見・気づき等を自由記載でお聞きいたしますので、これまでの経過を振り返っておいてください。 ご協力、本当にありがとうございました。それではあと1週間、よろしくお願いいたします！ | ⑫ Congratulations!  You've reached the final week of the trial period—well done! I understand that daily measurements may have been challenging amidst your busy schedule, but I sincerely thank you for your cooperation. By lending us various data, you've contributed to medical advancement. Please stay with us until the end. If you haven't yet scheduled your final measurement or need to change your appointment, please contact our research team as soon as possible. There will be a survey on a web form for the final measurement. We'll ask about your health and seek your overall impressions, opinions, and insights on the program, so please take a moment to reflect on your experience. Thank you very much for your cooperation. Let's make the most of this last week! |
