# Supplementary material for: A Brief Web-Based and Mobile Intervention of Intermittent Fasting With Meal Support for Weight Loss Among Adults With Overweight and Obesity in Japan: Pilot Randomized Controlled Trial
Source: JMIR Mhealth Uhealth. 2026 Jan 26;14:e58930. doi: 10.2196/58930 (PMC12887555; doi:10.2196/58930)
Supplement: Multimedia Appendix 3 [file mhealth_v14i1e58930_app3.pdf]

A Brief Web-Based Intervention of Intermittent Fasting with Meal Support for Weight Loss Among Overweight Japanese Adults: A Pilot Study of a Randomized Controlled Trial

Multimedia Appendix 3: Supplementary Information on the Research app

This is a Multimedia Appendix to a full manuscript published in the J Med Internet Res. For full copyright and citation information see http://dx.doi.org/10.2196/jmir.58930

I. Overview 2
II. Version and Bug Fixes Information 3
(1) iOS 3
(2) Android 3
III. Sign up 3
IV. Installation Manual Provided for Participants [in Japanese] 3

http://dx.doi.org/10.2196/jmir.58930

Ⅰ. Overview

Participants were instructed to install smartphone app 'Kenko-Nikki (Health Diary)'[1] provided by Healthtech Laboratory, Inc. (HTK). Originally, this app is an open Personal Health Record app available to the public on the App Store (Apple Inc., California, the U.S.) and Google Play Store (Google LLC, California, the U.S.). For this study, however, we used the closed test version tailored specifically for our study, which was published through Testflight (Apple Inc.) and DeployGate (DeployGate Inc., Tokyo, Japan).
HTK prepared the initial test version by duplicating the open version (ver.2.14.4) published on the App Store and Google Play Store on June 1, 2023. They established an independent server connected to the test app for this purpose. A bug-fixed version (ver.2.14.15) were published on June 7, 2023 and initially used in this study.
A representation of the Kenko-Nikki app interface is shown in **Figure S3-1**. In this app, users can log and record a wide range of health-related information, including anthropometric measures such as body weight, height, fat percentage, and clinical measures such as blood pressure, cholesterol levels, and COVID-19 status. The app also allows users to manage their health check-up data and medication status if needed. Users can also connect the app to other healthcare apps in order to automatically synchronize their health-related data. Users can check their records in a calendar or in graphical presentation.

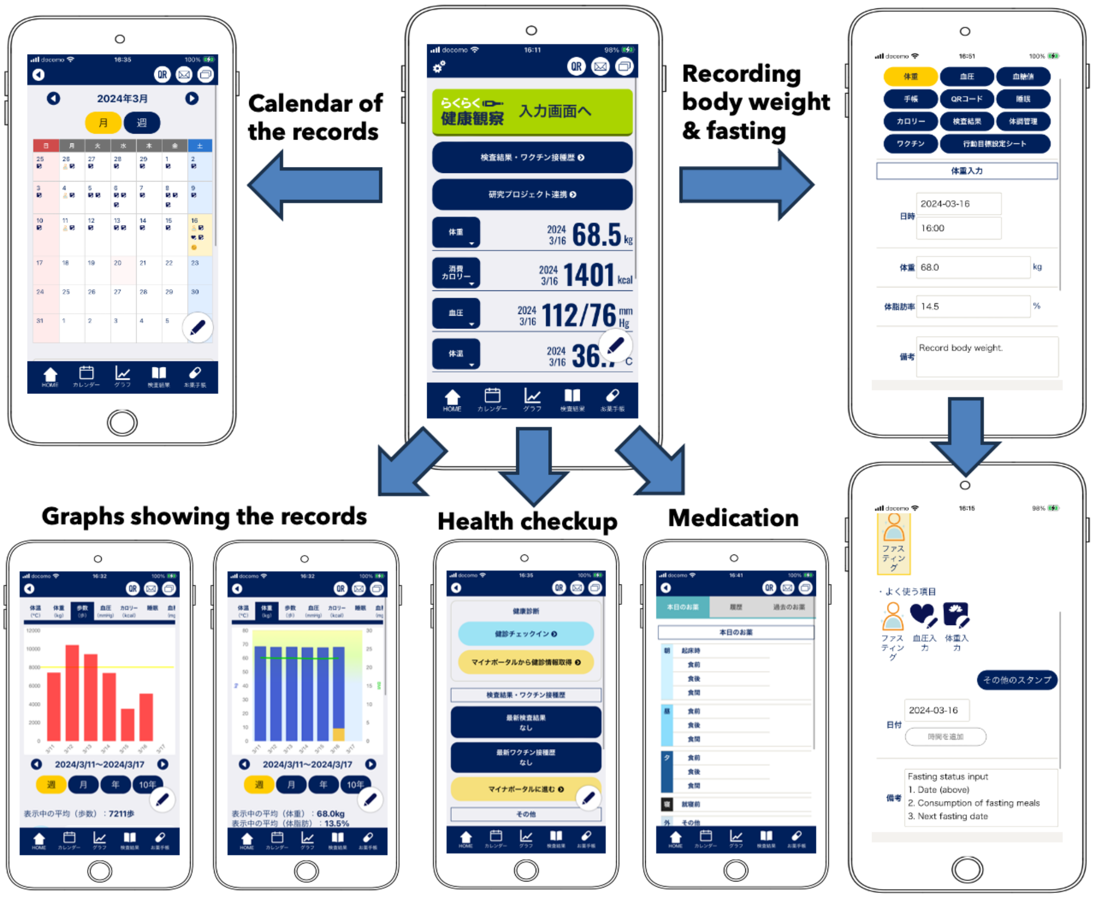

Figure S3-1. Kenko-Nikki app interface.

[1] Healthtech Laboratory, Inc.. Kenko-Nikki (Health Diary). https://htech-lab.co.jp/products/kenkounikki.html (Accessed February 21, 2024.)

In our study, participants were directed to log their step counts and body weight daily, either manually or through data-sharing with other apps. (We did not collect the information on whether participants recorded their step counts and weights manually or through data-sharing, though.)
In the home dashboard, users can proceed to the data-logging view by tapping the pencil icon on the bottom right.
To obtain exploratory data on the adherence to the intervention, a specific function for recording fasting status were developed for this test version app. Participants in the intervention group were directed to record the following information on their fasting day: the date, the consumption status of each fasting meal (extent to which they ate, and whether they ate other food or not), and the planned date of the next fasting-day. These data were predetermined to be assessed only exploratorily.

Ⅱ. Version and Bug Fixes Information

(1) iOS
June 1, 2023 (ver. 2.14.4): Initial test version.
June 7, 2023 (ver. 2.14.5): An error was identified where users were unable to register in version 2.14.4. This bug was fixed in version 2.14.5.
September 16, 2023 (ver. 2.15.341): Update required due to the 90-day expiration on Testflight.
December 16, 2023 (ver. 2.18.1): Update required due to the 90-day expiration on Testflight.

(2) Android
June 1, 2023 (ver. 2.14.4): Initial test version.
June 7, 2023 (ver. 2.14.5): An error was identified where users were unable to register in version 2.14.4. This bug was fixed in version 2.14.5.

Ⅲ. Sign up

We provided participants with an installation manual presented in Chapter IV, which included the essential URL and QR code for app registration. During the sign-up process, participants were required to create a password and to input their nickname, email address, birthdate, and place of residence (restricted to prefecture). Additionally, they were instructed to enable the data-sharing setting to automatically synchronize their step counts and body weight from other healthcare apps, such as the ‘Health’ app for iOS and the ‘Google Fit’ app for Android.

Ⅳ. Installation Manual Provided for Participants [in Japanese]

Instruction for iOS are followed by that for Android below.

研究用のためサーバの接続先がアプリストア版とは異なります。
アプリストアに公開されている健康日記アプリとのデータ引き継ぎが
できませんことご了承ください。

ファスティング試験用
アプリ

インストール・利用説明書
【iPhone】

機密

すでに（アプリストアに公開されている）健康日記を使っている方へ

＜実験開始時＞

１．今のアプリでバックアップを保存します。

※バックアップ方法は「機種変更時の操作：データのバックアップ」を参照ください。
（ログイン情報も保存しておいてください。）

２．今のアプリをアンインストールします。

３．本試験用アプリをインストールしてください。
※ここから他の参加者と同じ手順です。

＜実験終了後＞

１．本試験用アプリをアンインストールしてください。

２．AppStoreから「健康日記」をインストールしてください。

３．開始時１でメモしておいたログイン情報でログインしてください。

４．バックアップからデータを復元してください。

※アプリストアに公開している版と、本試験用アプリを併用することはできません。
※試験中のデータは消失します。

機密

初回インストール手順：試験用アプリのインストール

AppStoreに「TestFlight」というアプリを探してインストールします。
（下のQRコードからも
　インストール可能です）
※プッシュ通知も有効に
　してください。

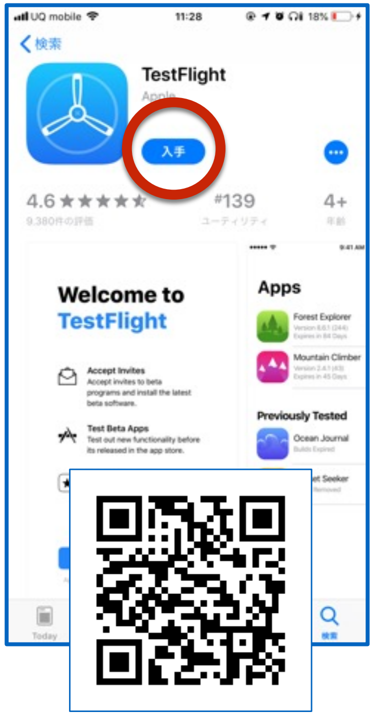

TestFlight用QR

カメラアプリで、こちらのQRコードを読み取ります。
読み取れない場合以下のURLに直接アクセスしてください。
https://testflight.apple.com/join/BM4yZSg5

モニターアプリ用QR

ステップ２の「テストを開始」ボタンをクリックします。
（この画面が表示されないこともあります）

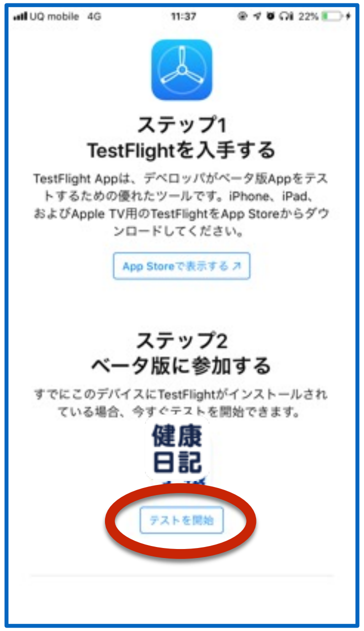

TestFlightのアプリが開くので「同意する」ボタンを押します。
インストールがはじまらない場合はさらに「インストール」ボタンを押してください。

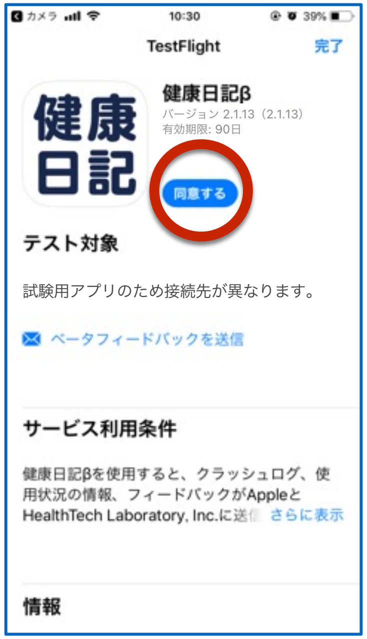

機密

初回インストール手順：試験用アプリのインストール

「次へ」ボタンを押してください。

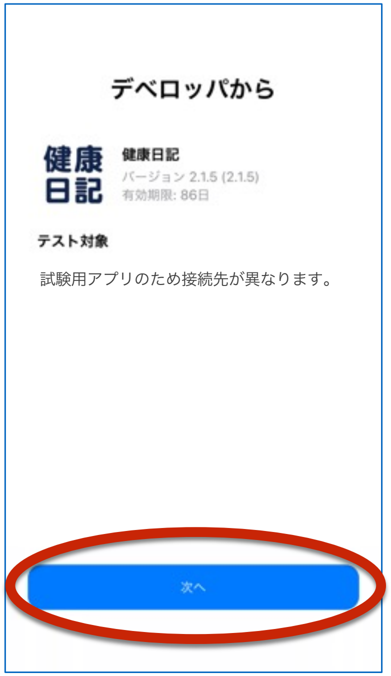

「テスト開始」を押してください。アプリが開きます。

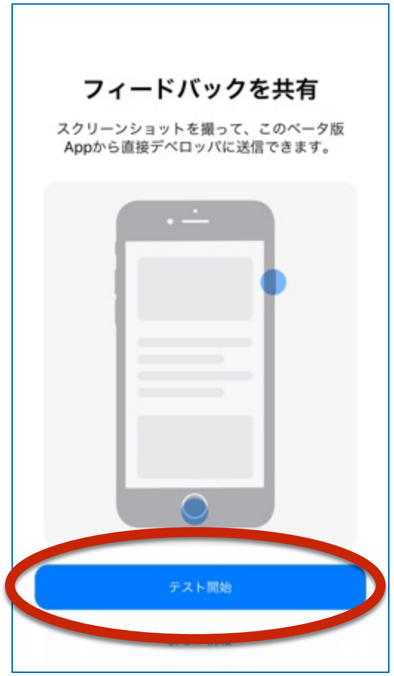

機密

初回インストール手順：アプリの開始

健康日記アプリを起動して
「設定する」からアプリを開始
してください。

利用規約とプライバシーポ
リシーをご確認の上、
「同意する」ボタンを
タップしてください。

「無料ユーザ登録」ボタン
をタップして登録してくだ
さい。

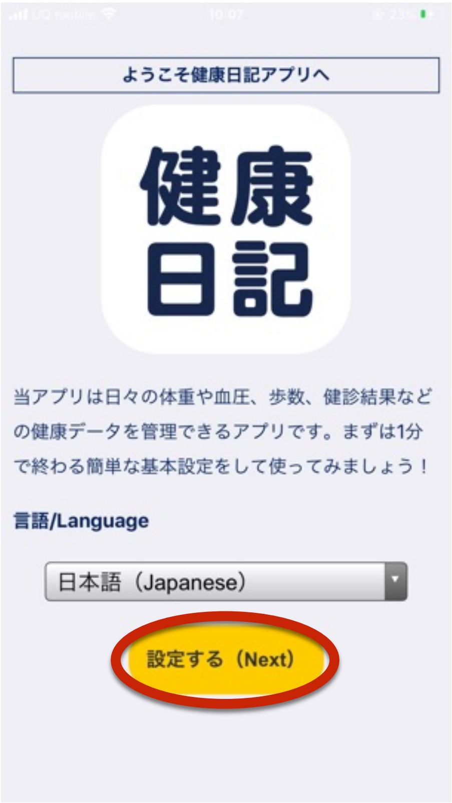

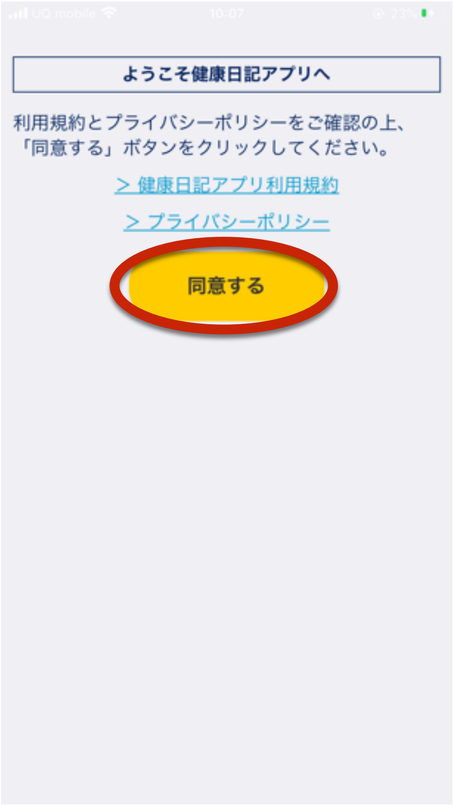

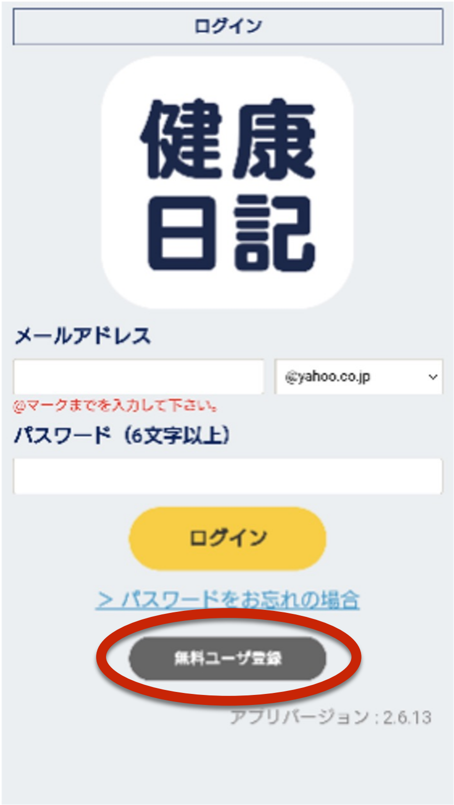

初回インストール手順：新規登録

プッシュ通知の許可の画面が出ます。「許可」をしてください。

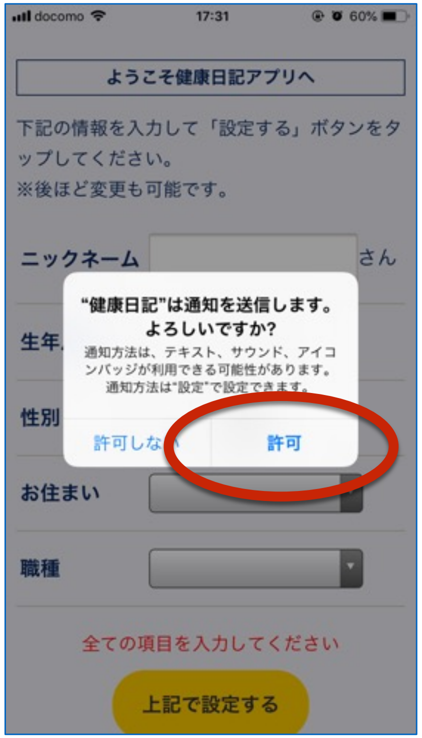

プロフィール情報設定画面が表示されたらログイン成功です。

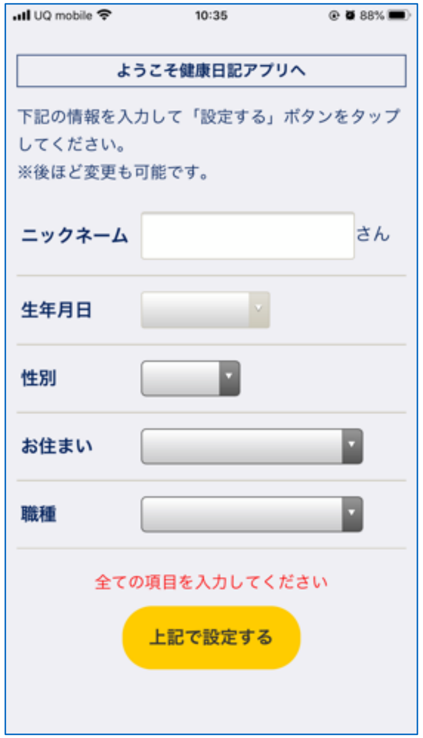

機密

初回インストール手順：初期設定（１／２）

プロフィール情報を入力して「上記で設定する」ボタンを押してください。
※後ほど変更も可能です。
※ニックネームは何でも大丈夫です。

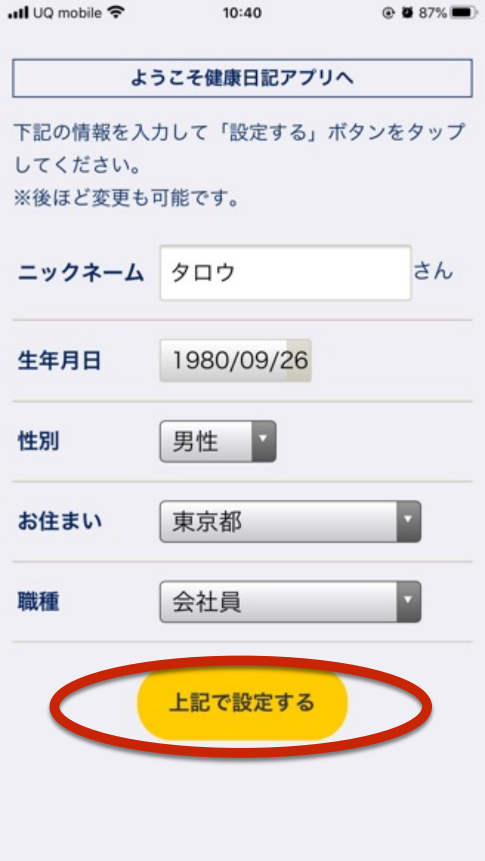

入力した内容に誤りがなければ「アプリをスタート」ボタンを押してください。

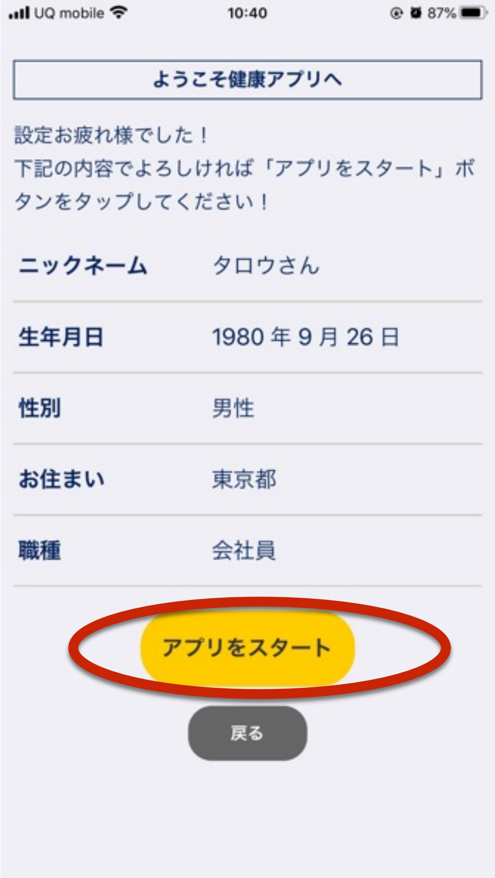

データのアクセス許可画面が表示されるのですべてのカテゴリをオンにしたあと「許可」ボタンを押してください。
※データは送信されません。

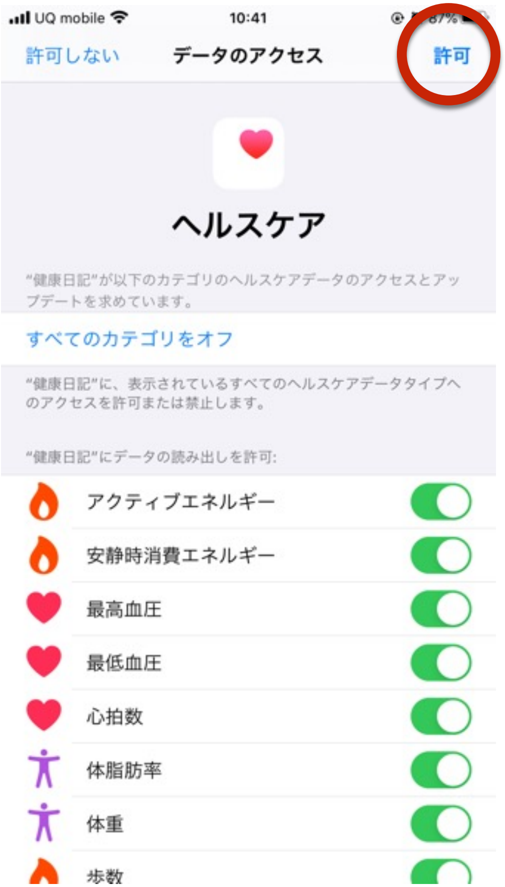

初回インストール手順：初期設定（２／２）

アプリの初期設定は完了です。

この画面は「HOME」です。
HOME画面に戻りたい場合は左下の「HOME」ボタンからお願いします。

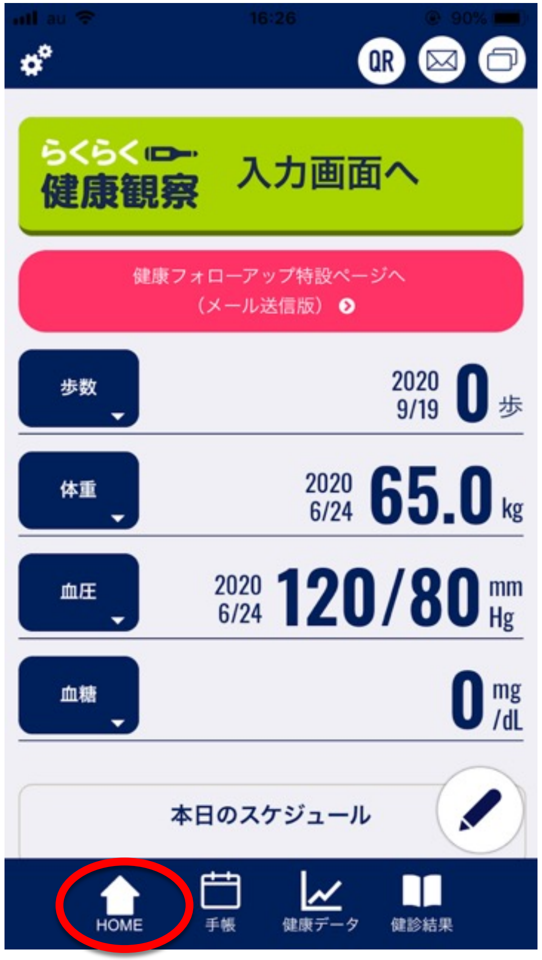

機種変更時の操作（1/2）：データのバックアップ

- ●機種変更などの際、データは自動では引き継がれません。
- ●データを引き継ぐためには、旧端末でまずバックアップを取ってください。
  （バックアップ用パスワードは保存しておいてください）

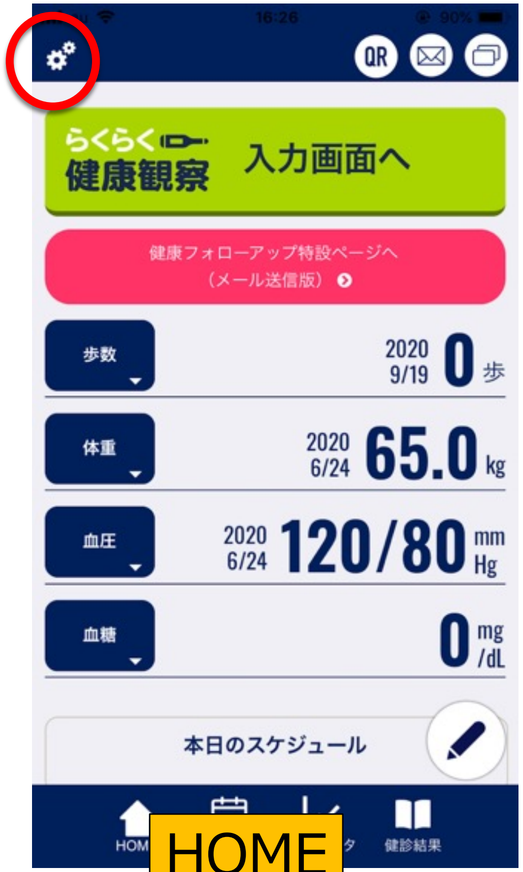

HOME

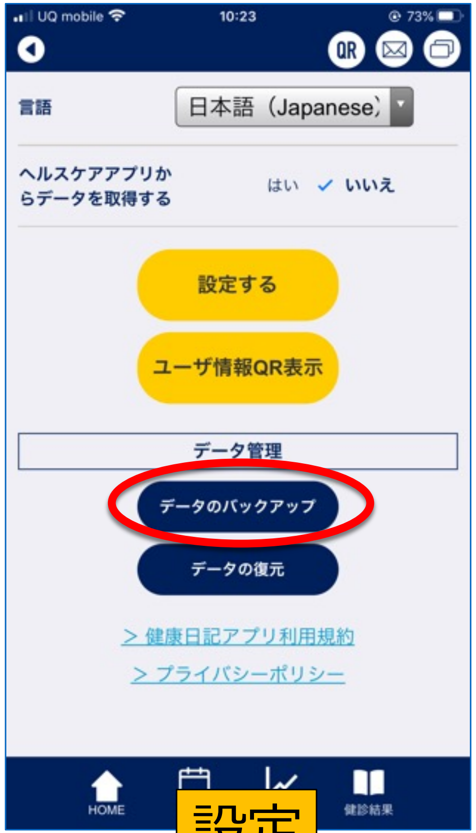

設定

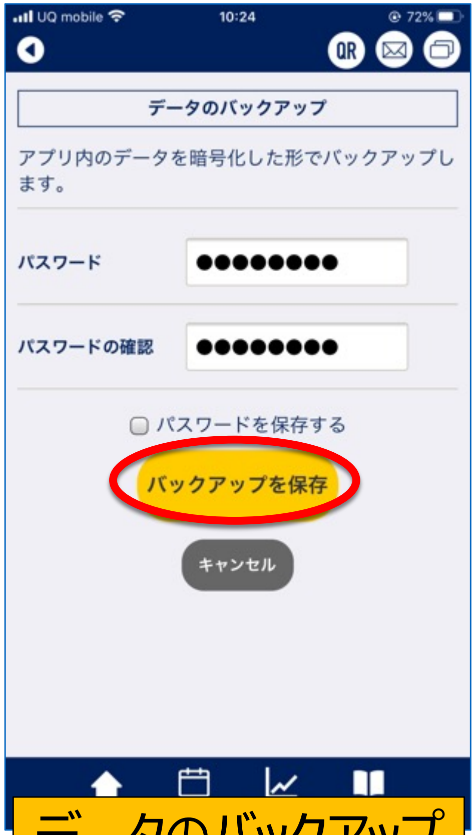

データのバックアップ

次ページへ続きます。

機種変更時の操作（2/2）：バックアップデータの復元

- 新端末にてログイン後、バックアップしたデータの復元を行ってください。
  （バックアップ保存時に設定したバックアップ用パスワードが必要です）

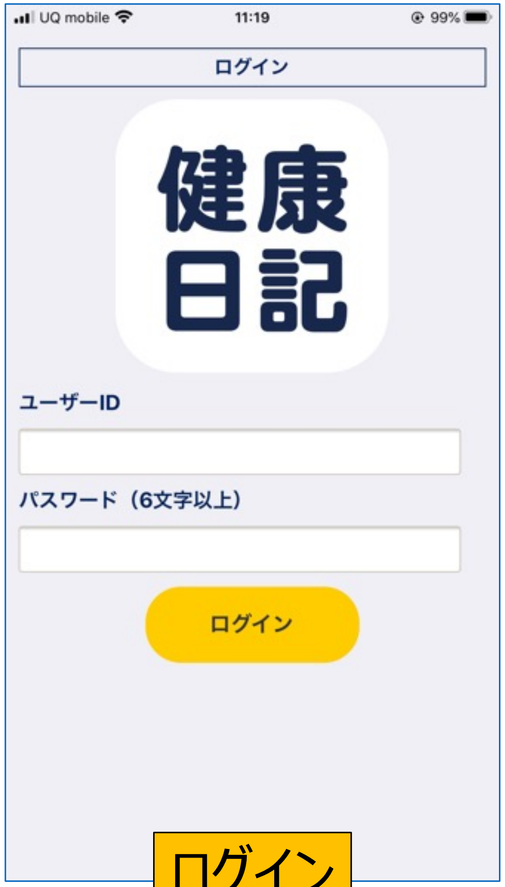

ログイン

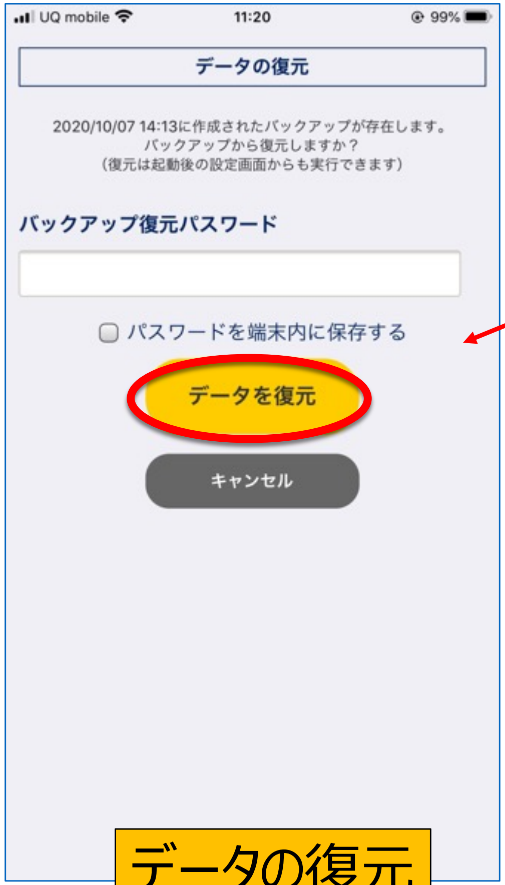

データの復元

バックアップをしている場合
ログイン後
こちらの画面が出現します。

研究用のためサーバの接続先がアプリストア版とは異なります。
アプリストアに公開されている健康日記アプリとのデータ引き継ぎができませんことご了承ください。

ファスティング試験用アプリ

インストール・利用説明書
【Android】

機密

すでに（アプリストアに公開されている）健康日記を使っている方へ

＜実験開始時＞

１．今のアプリでバックアップを保存します。

※バックアップ方法は「機種変更時の操作：データのバックアップ」を参照ください。
（ログイン情報も保存しておいてください。）

２．今のアプリをアンインストールします。

３．本試験用アプリをインストールしてください。
※ここから他の参加者と同じ手順です。

＜実験終了後＞

１．本試験用アプリをアンインストールしてください。

２．GooglePlayStoreから「健康日記」をインストールしてください。

３．開始時１でメモしておいたログイン情報でログインしてください。

４．バックアップからデータを復元してください。

※ GooglePlayStoreに公開している版と、本試験用アプリを併用することはできません。
※試験中のデータは消失します。

機密

初回インストール手順：実証実験用アプリのインストール（1/4）

こちらのQRコードを、QRコードリーダーアプリで読み取ります。読み取れない場合は以下のURLをブラウザで開いてください。
https://dply.me/hxlbwm

試験用アプリQR
（Android）

※QRコードリーダーアプリを入れていない場合は、Google Playストアから任意のQRコードアプリを入れてください。

ブラウザが開きますので「インストール」ボタンをタップしてください。

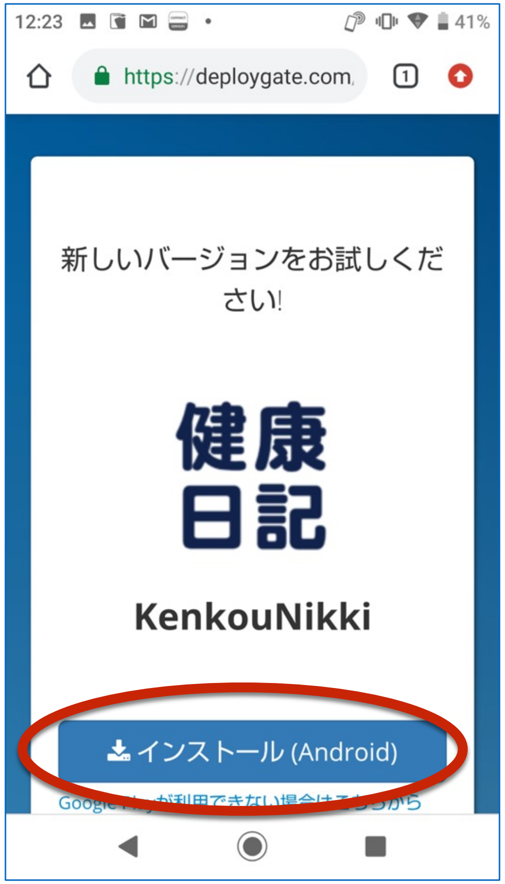

まずアプリ配布用ツールである「DeployGate」アプリのインストール画面に移動します。
「インストール」をタップします。

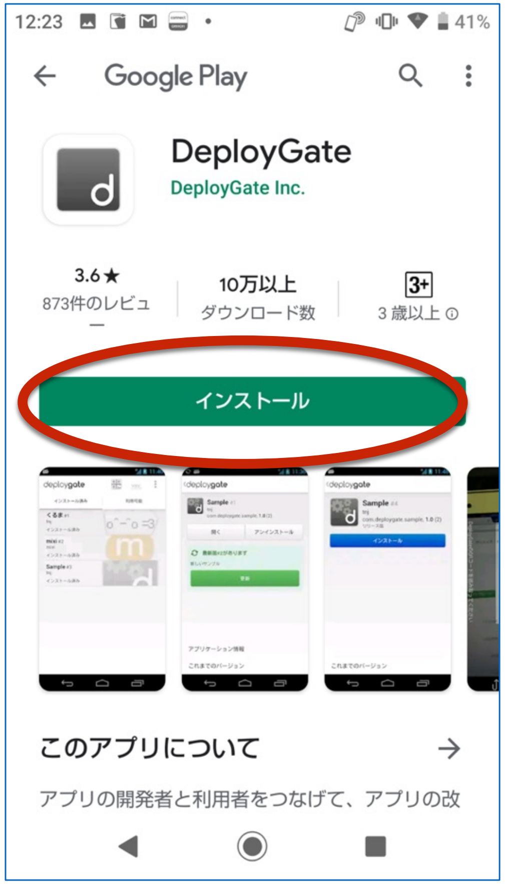

機密

初回インストール手順：実証実験用アプリのインストール（2/4）

「DeployGate」アプリの
インストールが完了したら
「開く」をタップします。

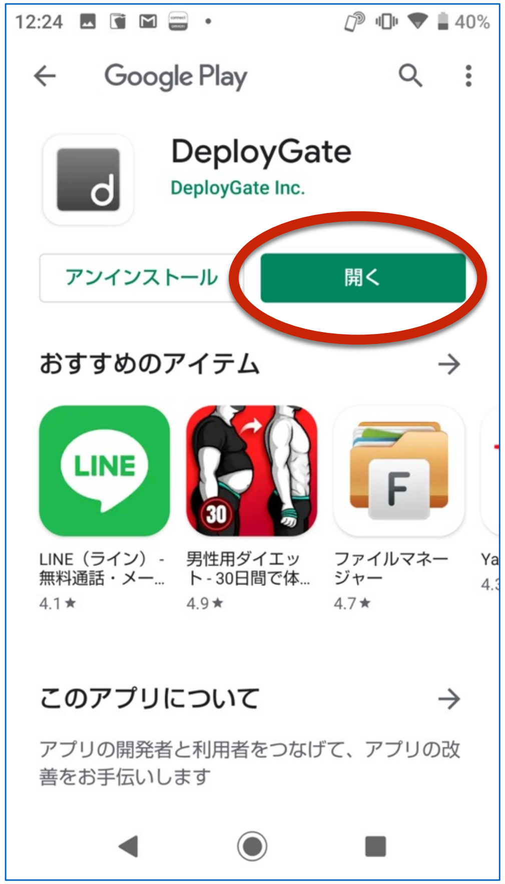

DeployGateアプリが開き、
睡眠プロンプト実験用アプリ
である「健康日記」のインス
トール画面が開きます。
「設定」をタップします。

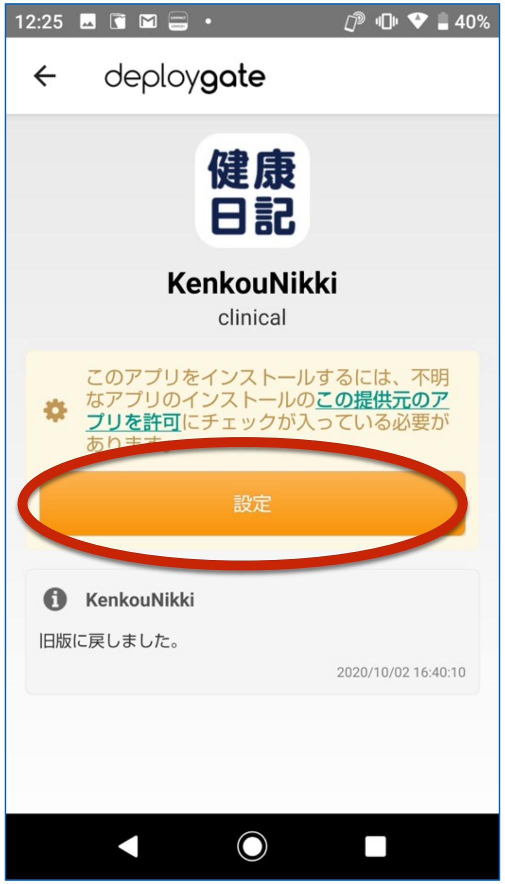

「この提供元のアプリを許
可」します。

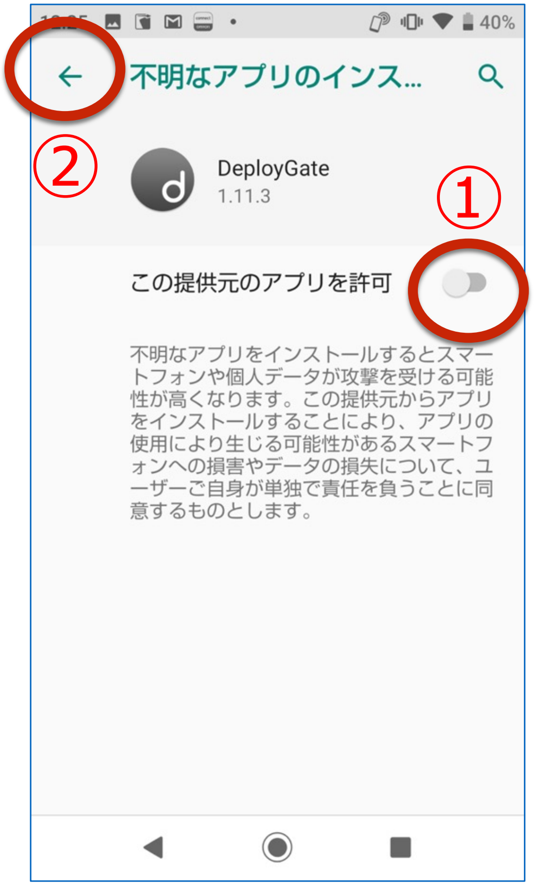

機密

初回インストール手順：実証実験用アプリのインストール（3/4）

DeployGateアプリに戻り

健康日記アプリの「インストール」ボタンをタップします。

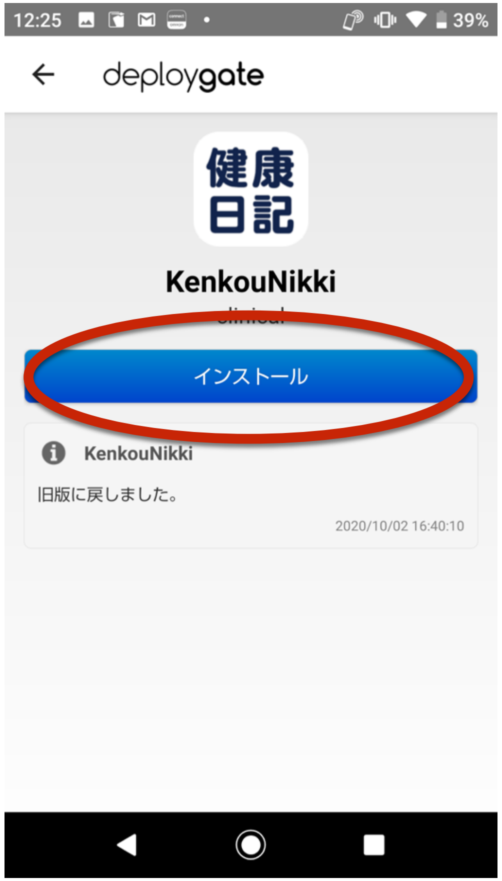

確認画面が出ますので「同意する」をタップします。

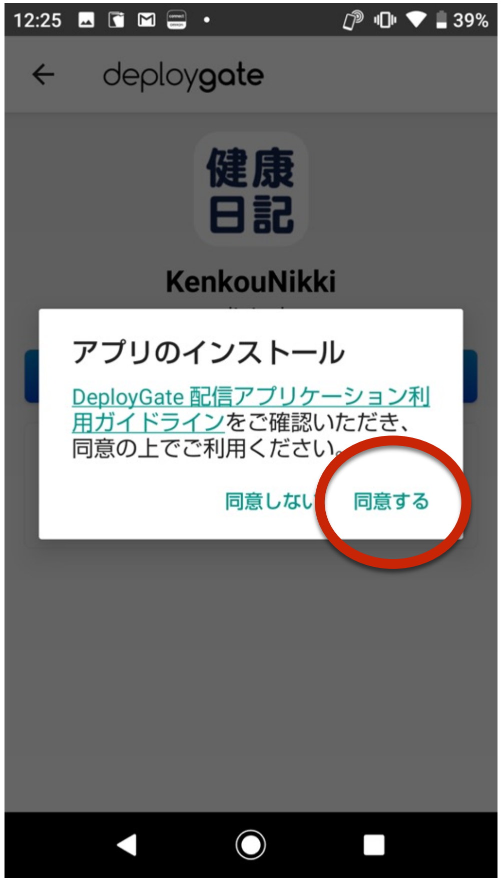

「インストール」をタップします。

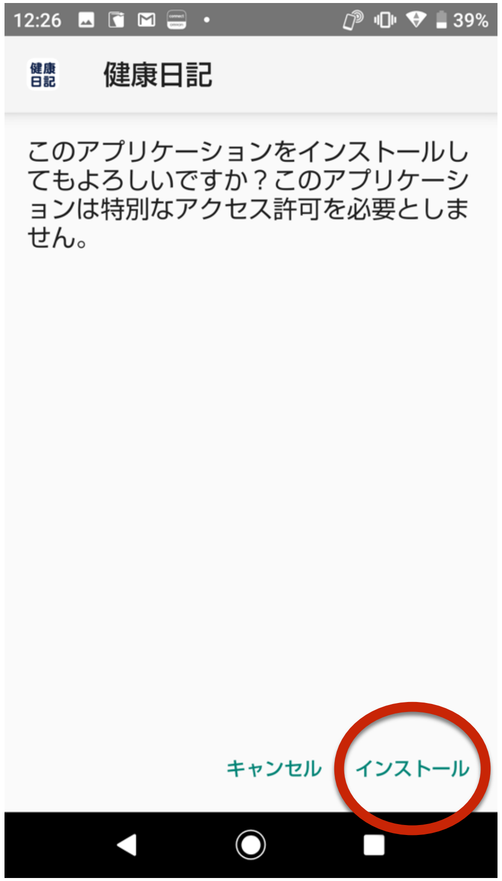

初回インストール手順：実証実験用アプリのインストール（4/4）

＜※ホーム画面への追加方法＞

次の画面が表示されたら、睡眠プロンプト実験用の「健康日記」アプリのインストールは完了です。

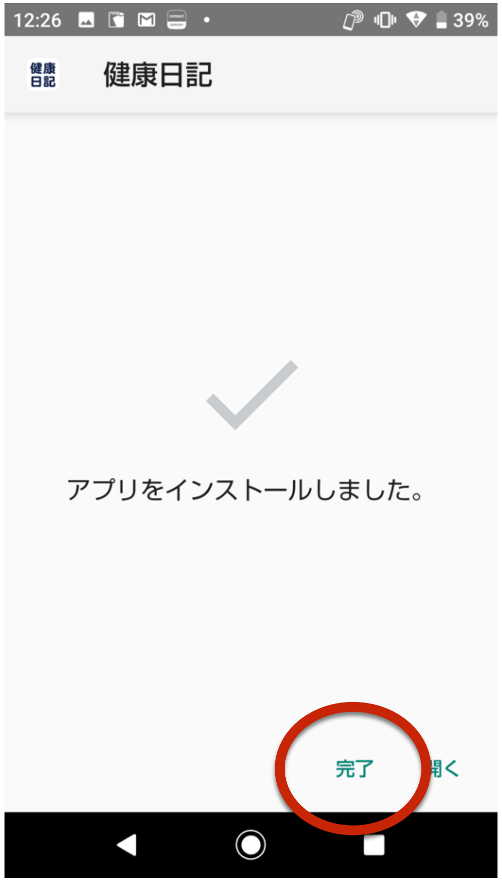

テスト用の配布方法のため、そのままではホーム画面に表示されません。
アプリ一覧画面を開いてください。

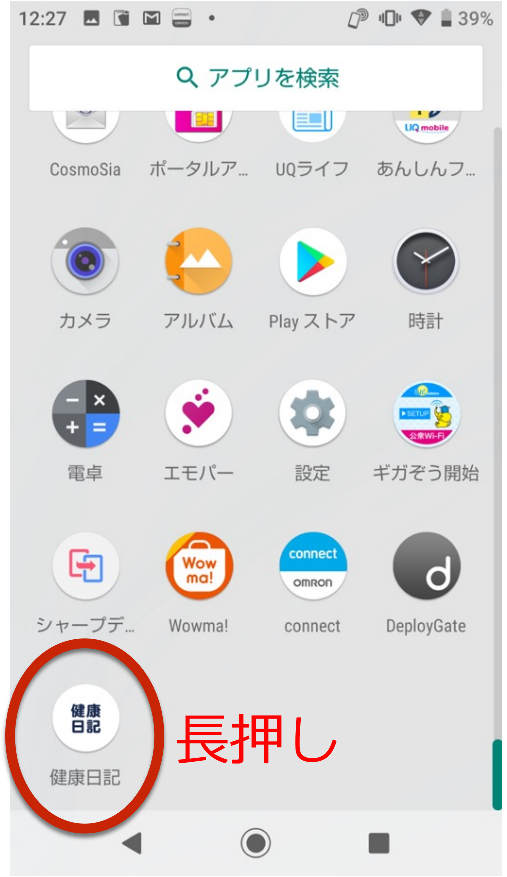

アプリ一覧画面で「健康日記」のアイコンを長押しすることで、ホーム画面に追加ができます。

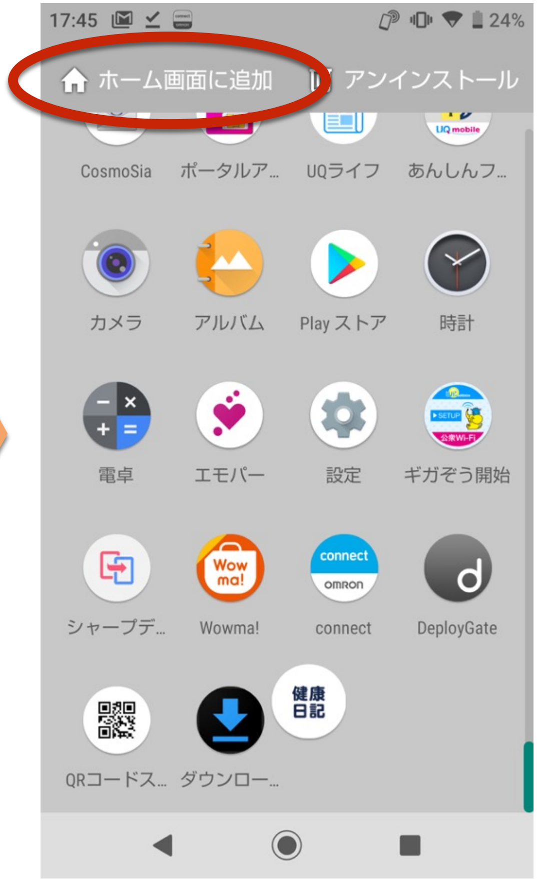

機密

初回インストール手順：アプリの開始

健康日記アプリを起動して
「設定する」からアプリを開始
してください。

利用規約とプライバシーポ
リシーをご確認の上、
「同意する」ボタンを
タップしてください。

「無料ユーザ登録」ボタン
をタップして登録してくだ
さい。

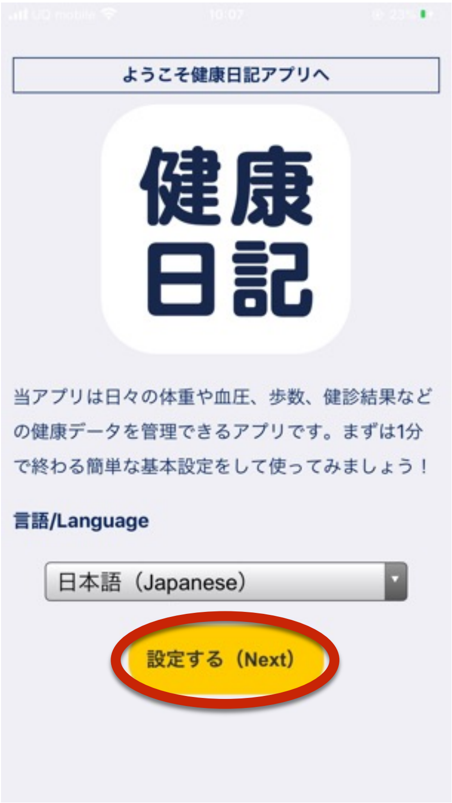

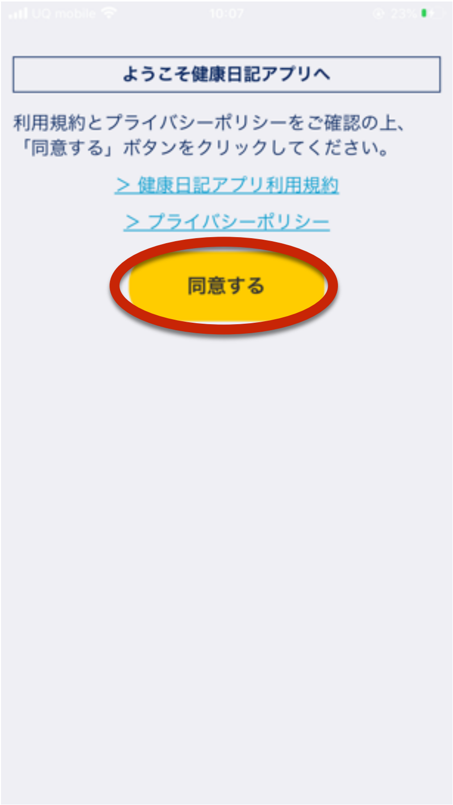

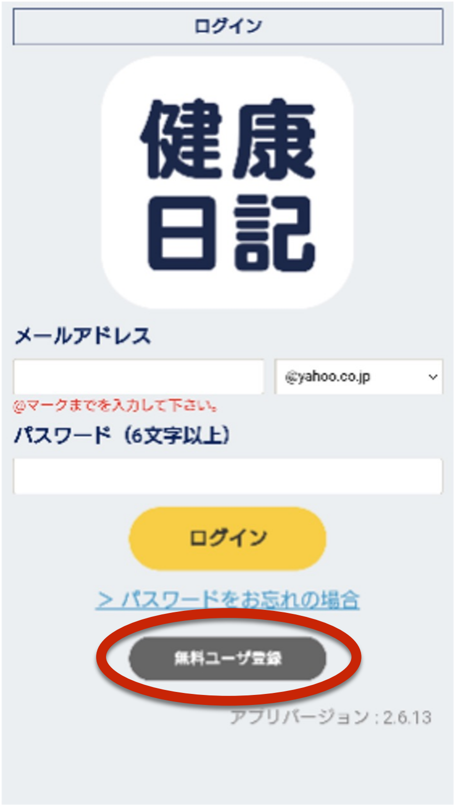

初回インストール手順：新規登録

プッシュ通知の許可の画面が出ます。「許可」をしてください。

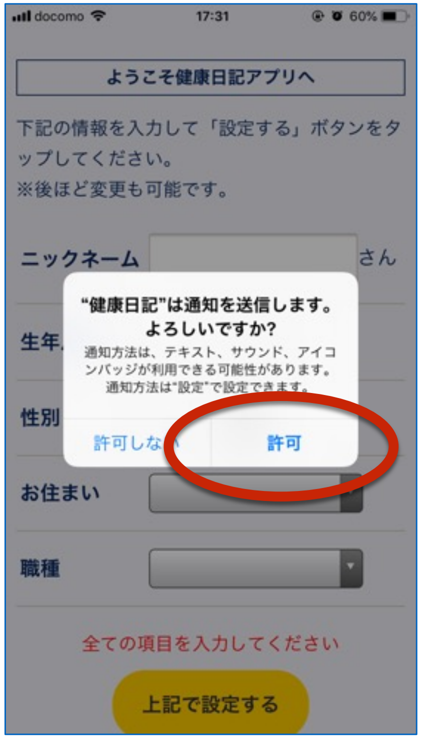

プロフィール情報設定画面が表示されたらログイン成功です。

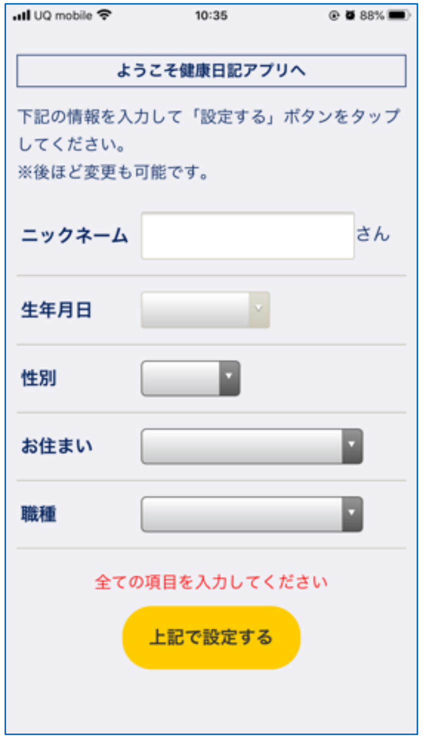

機密

初回インストール手順：初期設定（１／２）

プロフィール情報を入力して「上記で設定する」ボタンを押してください。
※後ほど変更も可能です。
※ニックネームは何でも大丈夫です。

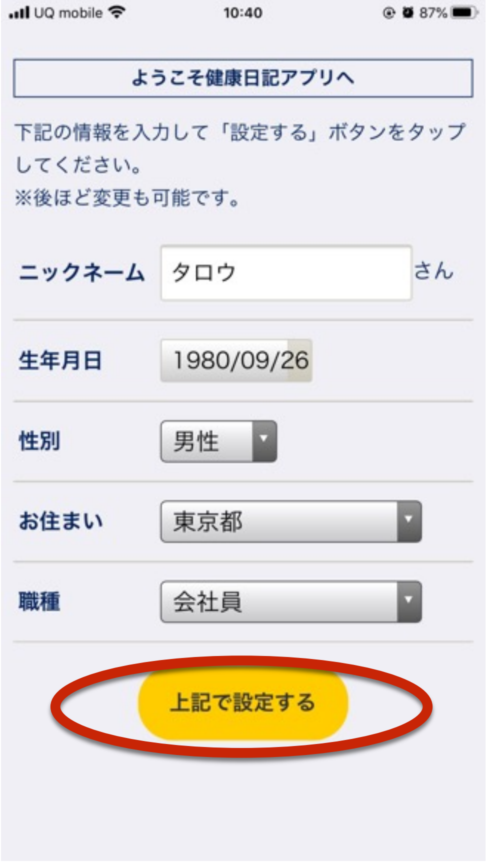

入力した内容に誤りがなければ「アプリをスタート」ボタンを押してください。

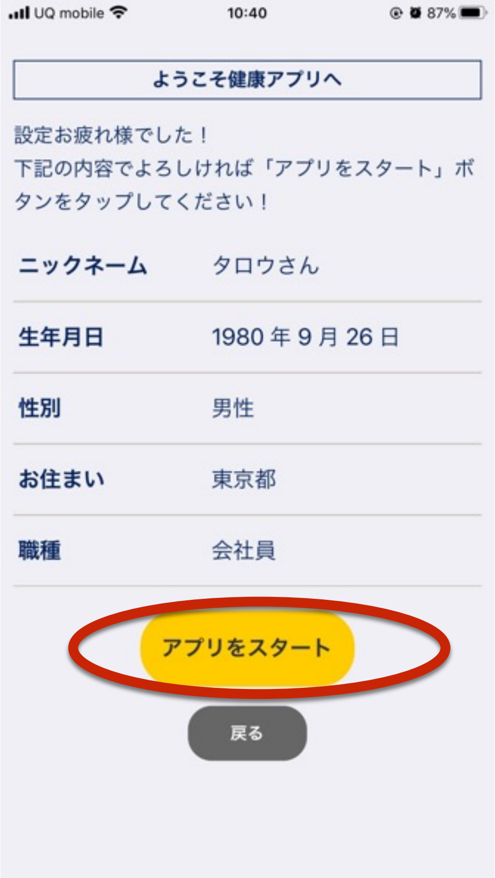

Google Fitがインストールされていない場合にはダイアログが表示されますが、無視して問題ありません。
※「OK」を押すとGoogle Fitのインストールページに飛びます。

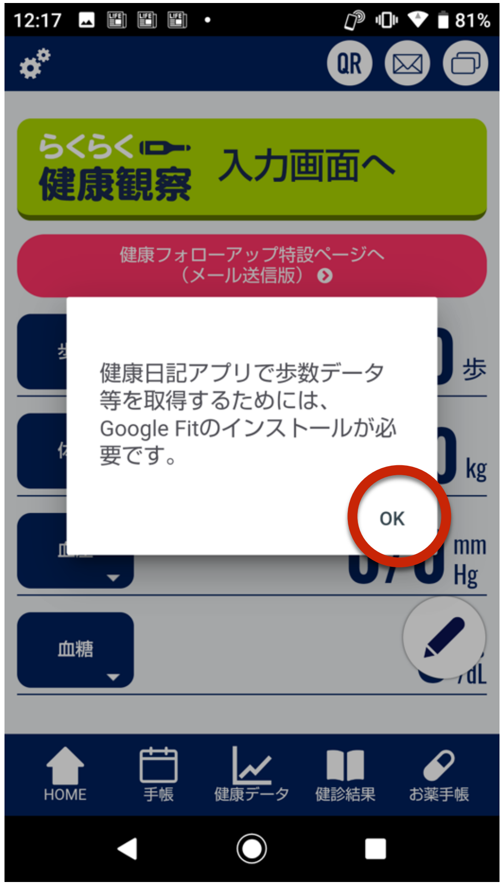

初回インストール手順：初期設定（２／２）

アプリの初期設定は完了です。

この画面は「HOME」です。
HOME画面に戻りたい場合は左下の「HOME」ボタンからお願いします。

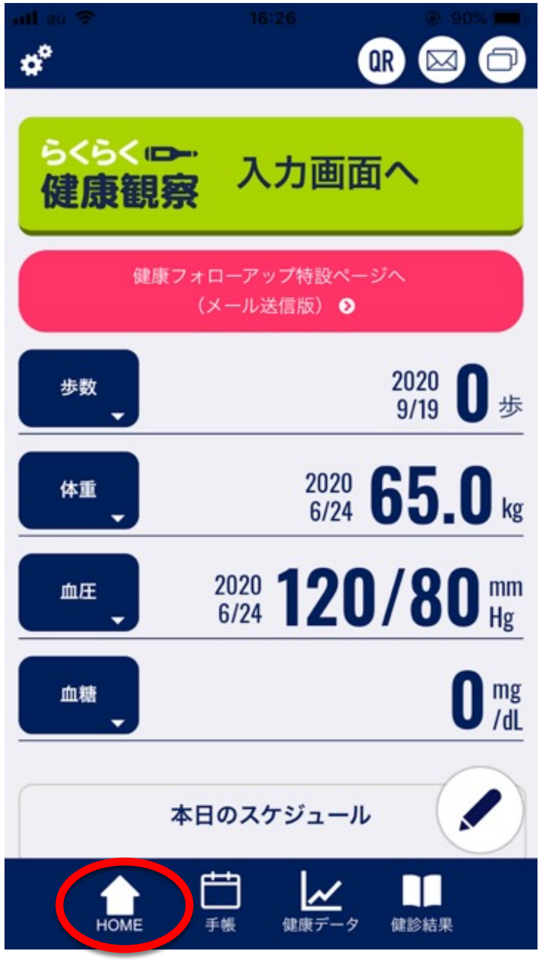

機種変更時の操作（1/2）：データのバックアップ

●機種変更などの際、データは自動では引き継がれません。
●データを引き継ぐためには、旧端末でまずバックアップを取ってください。
（バックアップ用パスワードは保存しておいてください）

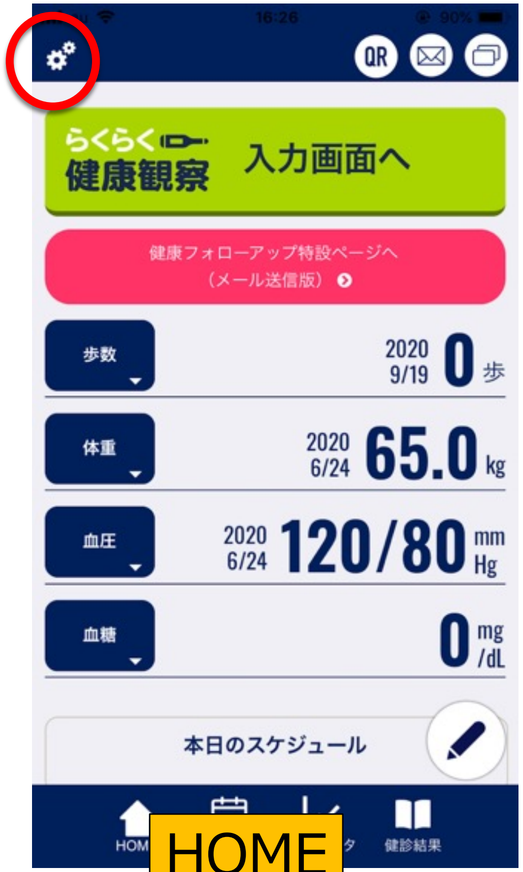

HOME

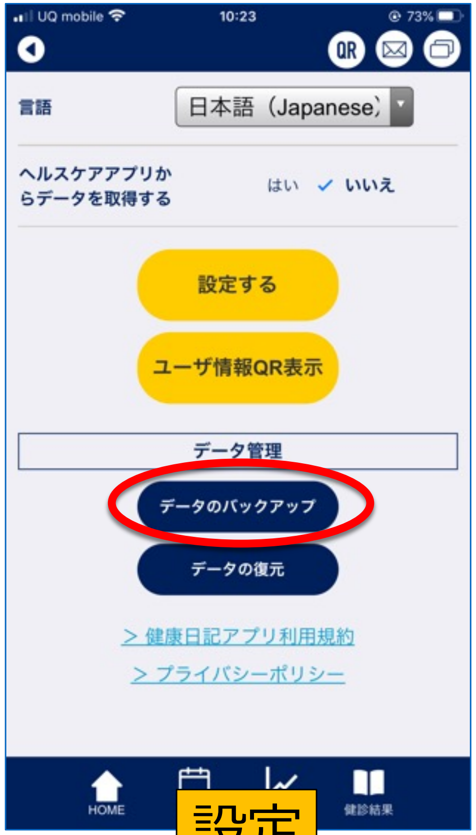

設定

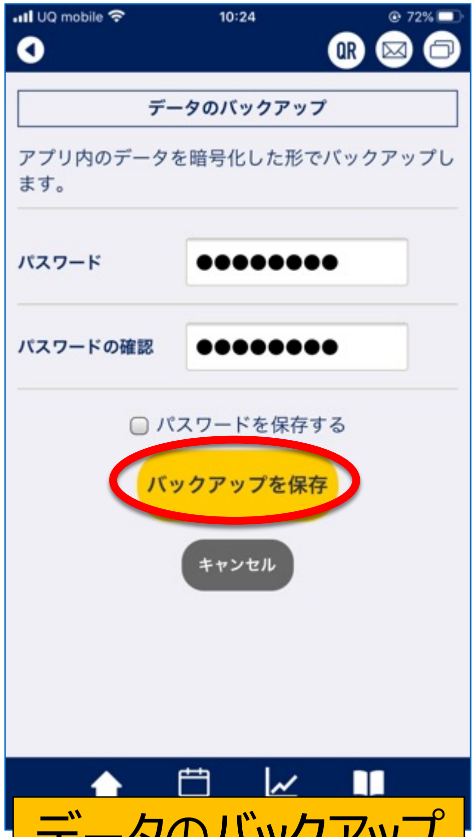

データのバックアップ

次ページへ続きます。

機密

機種変更時の操作（2/2）：バックアップデータの復元

- 新端末にてログイン後、バックアップしたデータの復元を行ってください。（バックアップ保存時に設定したバックアップ用パスワードが必要です）

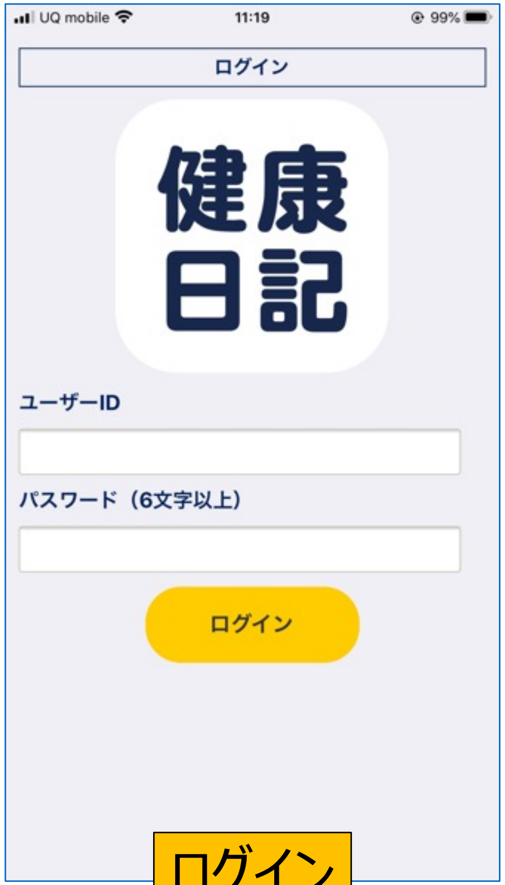

ログイン

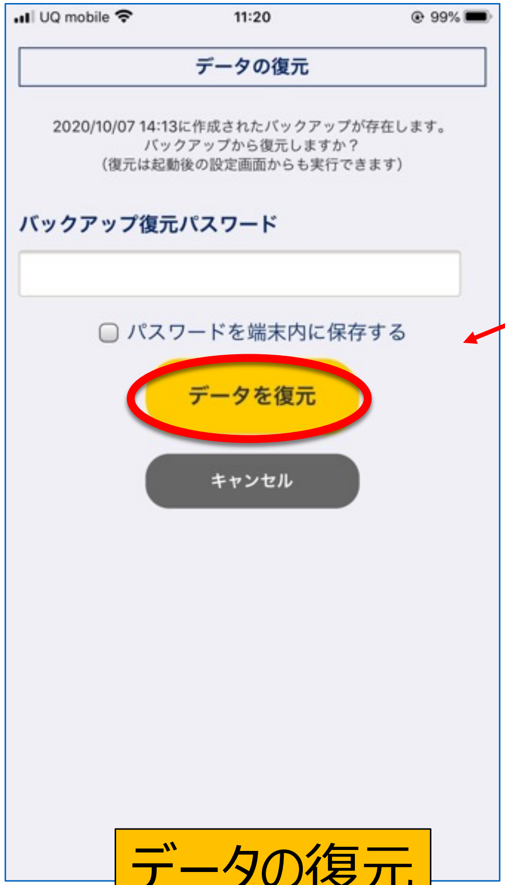

データの復元

バックアップをしている場合
ログイン後
こちらの画面が出現します。
