# Supplementary material for: A Brief Web-Based and Mobile Intervention of Intermittent Fasting With Meal Support for Weight Loss Among Adults With Overweight and Obesity in Japan: Pilot Randomized Controlled Trial
Source: JMIR Mhealth Uhealth. 2026 Jan 26;14:e58930. doi: 10.2196/58930 (PMC12887555; doi:10.2196/58930)
Supplement: Multimedia Appendix 4 [file mhealth_v14i1e58930_app4.pdf]

http://dx.doi.org/10.2196/jmir.58930
Multimedia Appendix 4
Statistical Analysis Plan
2024/04/03

A Brief Web-Based Intervention of Intermittent Fasting with Meal Support for Weight Loss Among Overweight Japanese Adults: A Pilot Study of a Randomized Controlled Trial

Multimedia Appendix 4:
Statistical Analysis Plan

This is a Multimedia Appendix to a full manuscript published in the J Med Internet Res. For full copyright and citation information see http://dx.doi.org/10.2196/jmir.58930

http://dx.doi.org/10.2196/jmir.58930
Multimedia Appendix 4
Statistical Analysis Plan
2024/04/03

A Brief Online Intermittent Fasting for weight loss: A Randomized Pilot Study.

General name： Online IF Study

Statistical Analysis Plan

Principal Investigator:
Professor, Taku Iwami
School of Public Health, Kyoto University
Department of Preventive Services

Author of this article:
Takashi Noda
School of Public Health, Kyoto University
Department of Preventive Services

Statistical Analysis Conductor:
Specific Assistant Professor, Norihiro Nishioka
School of Public Health, Kyoto University
Department of Preventive Services

January 11, 2024. Statistical Analysis Plan Ver.1.0
January 12, 2024. Statistical Analysis Plan Ver.1.1
January 13, 2024. Statistical Analysis Plan Ver.1.2
January 15, 2024. Statistical Analysis Plan Ver.2.0
February 28, 2024. Statistical Analysis Plan Ver.3.0 (This file)

http://dx.doi.org/10.2196/jmir.58930
Multimedia Appendix 4
Statistical Analysis Plan
2024/04/03

I. Overview 1
1. Primary Outcome 1
2. Secondary Outcomes 1
3. Safety Analysis 1
II. Statistical Protocol 1
1. Interim Analysis 1
2. Target Sample Size and its rationale 1
4. Criteria on Study Termination 2
5. Handling of Missing, Excluded, and Outlying Data 2
4. Changes to the original analysis plan 3
5. Definitions of Analysis Population 3
Ⅲ. General Statistics 4
1. Summary Statistics 4
Ⅳ. Statistical Analysis 5
1. Participants flow 5
2. Participants characteristics 5
3. Primary outcome 6
4. Secondary outcomes 6
5. Safety Analysis 7
Ⅴ. Signature 7
Ⅵ. Alterations after the study commencement 8
Ⅶ. Versions and Modifications 9
Ⅷ. Original Japanese Version 9

Ⅰ. Overview

This Statistical Analysis Plan elaborates on the details of Section 5.(4) "Overview of Analysis" in the research protocol for the randomized pilot study titled " A Brief Online Intermittent Fasting for weight loss: A Randomized Pilot Study. " Version 2.7.

1. Primary Outcome

Changes in body weight

2. Secondary Outcomes

1) Changes in continuous variables
   i. Body composition metrics (BMI, fat mass, muscle mass, basal metabolic rate)
   ii. Blood pressure indicators (systolic blood pressure, diastolic blood pressure)
   iii. Blood markers (hemoglobin A1c, triglycerides, total cholesterol, HDL cholesterol, LDL cholesterol)
   iv. Quality of Life (QoL) values (two component summary scores from SF-12v2)
   v. Physical activity levels (total physical activity as measured by the IPAQ short form)
2) Feasibility
   i. Drop-out rate, Adherence rate
   ii. Read rate and impact of weekly messages delivered via the research app
3) Exploratory Analysis
   i. Weight and step counts self-recorded by participants in the PHR app ‘Kenko-nikki’
   ii. Evaluation and feedback on the intervention program

3. Safety Analysis

1) Incidence, number of cases, and proportion of adverse events and minor symptoms.
2) Quality of Life (QoL) values (two component summary scores)

Ⅱ. Statistical Protocol

1. Interim Analysis

No interim analysis will be conducted.

2. Target Sample Size and its rationale

The target sample size is set at 40 participants (20 per group). Estimating an effect size (Cohen’s d) of 0.5 for future confirmatory randomized controlled trials based on this pilot study, Whitehead's recommendation would require 10 participants per group under conditions of a significance level of 0.05 and a power of 0.80. However, due to the objective of estimating the

proportion of participants who can adhere to the program with a simple online intervention in this pilot study, a sample size of twice that number, 20 participants per group totaling 40, is deemed necessary.

Estimated effect size for future confirmatory randomized controlled trials: Referring to the statistical results of NIPPON DATA90 and estimating the daily energy intake (requirement) of the target participants (adults aged 20-65 with BMI≧23) at about 2000 kcal, the intervention program being tested for effectiveness corresponds to an average energy restriction of about 1600 kcal per week. Therefore, the expected weight loss in the intervention group is approximately 2.7kg (=1600×12÷7200). Considering the results of recent online weight loss intervention studies conducted over 12 weeks using smartphone apps among Japanese populations, with an expected weight loss of 0.7kg in the control group and an assumed standard deviation of 4.0kg for both groups, the effect size (Cohen's d) is calculated to be 0.50.

3. Significance level

The significance level for tests will be set at a two-tailed 5%. The confidence intervals for outcomes will be based on 95% confidence intervals (95% CI) from two-tailed tests. P-values will be rounded to three decimal places, with values less than 0.001 displayed as <0.001 if rounding to three decimal places results in 0.000. No adjustment for multiplicity will be made in the analysis of secondary outcomes.

4. Criteria on Study Termination

The clinical study will not be stopped as a principle. However, it may be discontinued if serious adverse events occur that are suspected to be causally related to the intervention or observation, or if the research becomes difficult to conduct due to natural disasters, illness, or death of the researchers.

5. Handling of Missing, Excluded, and Outlying Data

1) Missing Data

For the primary outcome of weight, missing data at baseline measurement are unlikely due to the study design. Missing data at the final measurement due to dropout or loss to follow-up will be imputed using the Baseline Observation Carried Forward (BOCF) method, and analyzed based on the Intention to Treat (ITT) principle. This will constitute the primary analysis results. As a sensitivity analysis, an analysis will also be conducted where the intervention group is completed with the worst-case scenario (the largest positive change in weight among measured data) and the control group with the best-case scenario (the largest negative change). For secondary outcomes, self-recorded weight and step counts by participants using the research app will be imputed using the Last Observation Carried Forward (LOCF) method, while other missing data will be imputed using BOCF. Missing data for the read rate and impact of weekly messages delivered via the research app will be imputed as zero.

2) Excluded Data

Exclusion of outliers will not be conducted as a principle. If outliers are excluded, the details and reasons will be reported.

3) Outlying Data

For the primary outcome of weight, no determination of outlying data will be made, and data will not be excluded. For the secondary outcome of physical activity, according to IPAQ guidelines, responses less than 10 minutes (and their number of days) for each physical activity will be replaced with zero. Walking time, moderate physical activity time, and vigorous physical activity time exceeding "180 minutes" will be truncated to "180 minutes". For the secondary outcome of blood markers, the measurement range of the cobas b 101 plus instrument (Roche Diagnostics Ltd.) is as follows, and data outside this range will be displayed as NA (high / low). In such cases, if the error content is high, the upper limit of the measurement range will be used; if low, the lower limit will be adopted. For LDL cholesterol in cases of NA (when TG value is above 400mg/dL), it will be calculated using the Friedewald formula (CHOL−HDL−[TG÷5]), as is originally used by the instrument.

i. HbA1c: 4-14%
ii. Total Cholesterol (CHOL): 50-500 mg/dL
iii. HDL Cholesterol (HDL): 15-100 mg/dL
iv. LDL Cholesterol (LDL): 1-477 mg/dL

Triglycerides (TG): 45-650 mg/dL Other than mentioned above, outlying data will not be determined as a principle, and data will not be excluded. If data are excluded, the details and reasons will be reported.

4. Changes to the original analysis plan

In the event of changes to the original analysis plan, such as modifications to the research protocol that affect this Statistical Analysis Plan, the plan will be revised. The revisions will document the sections changed, the reasons for the changes, and these will be added to the change history.

5. Definitions of Analysis Population

The definitions of each analysis population are as follows. Analysis of primary and secondary outcomes will include all participants assigned, according to the Intention to Treat (ITT) principle. Participants who withdraw consent and stop participating in the study will be included in the baseline and final analysis if consent for the use of data collected before withdrawal (baseline measurement data) is granted. Participants who requested deletion of all their data will be excluded from baseline and final analysis but included in the numerator and denominator for calculations of dropout and adherence rates (described later in 4(a)). Sensitivity

analysis will also be conducted on the Per Protocol Set (PPS) to confirm the stability of the analysis results. Analysis of adverse events and mild symptoms related to safety will be conducted on the Completers Set (CS).

1) ITT

The entire group assigned, excluding participants who requested deletion of all data due to withdrawal of consent. Those who withdrew consent but allowed the use of baseline measurement data will be included in the analysis. Cases of loss to follow-up will be included in the analysis after imputing missing data due to the inability to perform the final measurement.

2) CS

The entire group from the ITT analysis population, excluding cases of loss to follow-up (those who completed the final measurement).

3) PPS

From the CS, the group comprising cases that meet the following criteria:

i. Intervention group

Those are ‘adherent’ and/or ‘fully adherent’ will be included to PPS, based on the response to the question, "How many times did you fast according to the instructions out of 12?" Participants who reported fasting 12 out of 12 times were categorized as ‘fully adherent,’ whereas those who fasted 10 or more were classified as ‘adherent.’

ii. Control group

Participants in the control group who adhered to the minimal care, whose who read at least 10 out of the 12 app messages (excluding the automatic message at registration), verified from the server data.

Ⅲ. General Statistics

1. Summary Statistics

The number and relative frequency (%) of males will be summarized, while other continuous variables will be summarized using median and interquartile range. Data for each variable will be organized by group for the intervention and control groups. Analysis of Outcomes: For body weight and other continuous variables, the mean change and standard deviation will be rounded to one decimal place beyond the number of decimal places +2. However, integers may also be used if deemed appropriate due to space constraints on paper or for the sake of table organization.

1) Frequency Distribution Tables

Relative frequencies will generally be rounded to one decimal place and presented as percentages. However, except for weight, BMI, and hemoglobin A1c, integers may also be used if deemed appropriate due to space constraints or for table organization.

2) Confidence Intervals

Two-sided 95% confidence intervals will be used. The number of decimal places for confidence intervals will follow the same rules as for point estimates.

3) Timing of Statistical Analysis

Analysis will be conducted after all data collection for all evaluation items is complete and the data is locked.

4) Statistical Analysis Environment

Takashi Noda, the researcher, will design analysis scripts on a MacBook Pro (macOS Ventura ver.13.6.1) using the software R (ver.4.3.2) and RStudio (ver.2023.12.0+369), ensuring the analysis can be conducted with test data in advance. After the final data collection and data locking, Norihiro Nishioka, the statistical analysis lead, will execute the scripts on a MacBook Pro Apple M2 using the software R (ver.4.3.2) and RStudio (ver.2023.12.0+369) and share the results.

Ⅳ. Statistical Analysis

1. Participants flow

In accordance with the CONSORT (Consolidated Standards of Reporting Trials) 2010 guidelines, a flowchart will illustrate the process of participant enrollment, allocation, follow-up, and analysis. This will include the number of participants who withdrew consent, were lost to follow-up, were unreachable, were included in the ITT (Intention to Treat) analysis population, completed the follow-up, and were part of the PPS (Per Protocol Set). Additionally, the dropout rate (the proportion of participants who withdrew consent or were lost to follow-up within the ITT analysis population) and the adherence rate (the proportion of the ITT analysis population that is part of the PPS) will be calculated for each group. If necessary, the p-value from the chi-square test will also be reported alongside these rates. Furthermore, within the intervention group, the proportions of "almost adherents" and "complete adherents," as defined in the protocol, will also be reported individually.

2. Participants characteristics

［Analysis population：ITT］

For sex, the number and relative frequency (%) of males will be calculated for each group. Continuous variables such as age, weight, BMI, height, fat mass, muscle mass, basal metabolic rate, systolic blood pressure, diastolic blood pressure, hemoglobin A1c, triglycerides, total cholesterol, HDL cholesterol, LDL cholesterol, QoL, and total physical activity will be summarized using median and interquartile range. For QoL, the electronic version of the SF-12v2 Japanese version provided by Qualitest Inc. will be used, summarizing the physical

and mental component summary scores (based on the 1995 nationwide survey in Japan) as summary indicators.

3. Primary outcome

［Analysis population：ITT, PPS］

The change in body weight, the primary outcome, will be analyzed as the main analysis using a generalized linear regression model, incorporating gender and age at randomization and weight at baseline as covariates. Point estimates of the difference in weight change between groups, along with 95% confidence intervals (CI) and p-values for the effect of the intervention, will be determined. Regression coefficients for each covariate will also be reported along with their p-values. As sensitivity analyses, comparisons of change without adjustment for covariates (using Welch's t test) and Per Protocol analysis in the PPS (with the same covariate adjustments as the main analysis) will be conducted. Additionally, if there are missing data for weight at the final measurement, analysis in the dataset where missing data are imputed with the worst-case scenario for the intervention group (corresponding to the least weight loss or the greatest weight gain among measured data) and the best-case scenario for the control group (corresponding to the greatest weight loss or the least weight gain among measured data) will be added as sensitivity analyses (both adjusted and unadjusted for covariates).

4. Secondary outcomes

［Analysis population：ITT］

For continuous data among secondary outcomes, the mean difference between groups without adjustment for covariates and the 95% confidence intervals (CI) in Welch's t-test will be calculated. The total physical activity, assessing lifestyle changes, will be analyzed for both between-group differences and within-group changes using the Wilcoxon signed-rank test.

The read rate of messages delivered via the research app (opening rate confirmed from server data) will be summarized by median and interquartile range for each group, and the p-value from the Wilcoxon rank-sum test will be reported. Additionally, the impact of messages delivered through the app on adherence to the program and lifestyle behaviors will be evaluated based on responses to the question in the final measurement web form survey, "To what extent did the messages delivered through the app influence your adherence to the program and lifestyle behaviors? (Please choose within the range of 0 [no influence at all] to 10 [greatly influenced])." The impact level of messages will be converted to a percentage from 0 to 100, summarized by median and interquartile range for each group, and the p-value from the Wilcoxon rank-sum test will be reported.

Self-measured weight and step counts will be analyzed as exploratory evaluations of the program's effects and feasibility, considering data from the first day of intervention (observation) as Day 1 through to Day 84 (12 weeks later).

5. Safety Analysis

［Analysis population：CS］

1) Adverse events and mild symptoms

For evaluation of the safety, the number of adverse events and the number of individuals experiencing them will be investigated. Participants were instructed to consult anytime throughout the study regarding any adverse events or symptoms. In addition, they will be asked at the 12-week measurement about the incidence of 20 symptoms pre-listed from prior study[1] (sleep difficulty, hunger, fatigue, headache, diarrhea, sensitivity to cold, dry mouth, back pain, bad breath, muscle pain, abdominal bloating, cravings, vertigo, blurred vision, restless leg, skin rash, nausea, palpitation, dyspepsia, muscular cramp) via a web form. We primarily evaluated the difference in the number of these 20 events and the number (%) of participants who reportedly experienced them. Additionally, at the final measurement, another web form questionnaire will ask, "During the trial period, regardless of its relevance to the study, did you experience any health-related adverse events (including accidents or injuries) or health problems?" to gather information on adverse events and symptoms not listed above.

From the data collected, the number of incidents, number of affected participants, and the incidence rate (%) for each group will be calculated. The incidence rate will be rounded to one decimal place as a percentage, with further rounding applied as necessary. The number of affected participants and incidence rates will be summarized by group in a table and reported along with the p-value from the chi-square test.

2) Quality of life（QoL）

As part of the safety assessment, changes in the two component summary scores for physical and mental aspects of QoL will be analyzed using the Wilcoxon signed-rank test for each group. Additionally, scores for each subscale will be evaluated exploratorily.

Ⅴ. Signature

I hereby affirm that the content of this Statistical Analysis Plan is appropriate as the statistical analysis plan for the "Randomized Controlled Trial of an Online Intermittent Fasting Program for Weight Loss: A Pilot Study," and that this Statistical Analysis Plan was finalized before the final data analysis.

Date:

____

[1] Wilhelmi de Toledo F, Grundler F, Bergouignan A, et al. Safety, health improvement and well-being during a 4 to 21-day fasting period in an observational study including 1422 subjects. PLoS One. 2019;14:e0209353 [Medline]

Ⅵ. Alterations after the study commencement

After the recruitment began, three collaborating facilities were added, and the initial eligibility criterion of having a BMI between 25 and less than 35 was modified to include individuals with a BMI between 23 and less than 35. This change required a revision of the protocol and a subsequent review by the Ethics Committee. Aspects of the statistical analysis not explicitly outlined in the original protocol were detailed in the statistical analysis plan (Multimedia Appendix 6), with its final version being established prior to the data lock. For example, baseline analysis was described in more detail, and a t-test was specified as Welch's t-test. The definitions of analysis population—ITT, PPS, CS—were also clearly defined in the statistical analysis plan.

Initially, adherence to the fasting practice was determined based on confirmation from any of the three sources: questionnaire, app, or a written note in a distributed leaflet. However, this definition was later changed to solely self-report via a questionnaire, due to the original criteria being deemed too complex and broad in scope. This modification was incorporated into the analysis plan, where it also served to define the PPS for the intervention group. Since adherence was originally defined only for the intervention group in the protocol, the analysis plan further clarified the adherence criteria for the control group, thereby concretely defining the PPS for this group.

Additionally, a post hoc analysis of the primary outcome was included to compare the number (%) of participants achieving a MIC.

Ⅶ. Versions and Modifications

Ver	Date	Author	Modifications and its Reasons.
Ver.1.0	2024/01/11	TN	Initiation
Ver.1.1	2024/01/12	TN	1. Changed the method for handling missing weight data from multiple imputation to BOCF (Baseline Observation Carried Forward) for a more conservative analysis and to simplify interpretation and analysis.<br>2. Removed mention of excluding physical activity data from secondary outcomes, as it is not subject to design-related cleaning issues.<br>3. Added a Completers Set (CS) to the analysis population. Safety analysis will focus on CS instead of ITT (Intention-to-Treat) to avoid diluting the proportions.<br>4. Included the statistical analysis environment, which was previously omitted.<br>5. Clarified that analyses in the worst-case completion scenario will also include multivariate analyses, unadjusted analyses, and per-protocol analyses.<br>6. Changed the analysis method for within-group differences in physical activity and Quality of Life (QoL) from paired t-tests to Wilcoxon signed-rank tests (to avoid assuming normality).<br>7. Adjusted the analysis of message read rates and impact from chi-square tests to Wilcoxon rank-sum tests, as the measure is a percentage but not a binary outcome of "present/absent"
Ver.1.1	2024/01/13	TN	1. For the changes in Version 1.1, point 5, exclude the worst-case completion from per-protocol analysis since completed samples are excluded from the PPS (Per Protocol Set), making it irrelevant.<br>2. Update the version information for R Studio.
Ver.2.0	2024/01/15	TN	1. Updated with the modification above into new version.
Ver.3.0	2024/02/27	TN	1. Translated Ver.2.0 into English.<br>2. Added the alterations after study commencement.<br>3. Attached the original Japanese version below.

Ⅷ. Original Japanese Version (Ver.2.0)

簡易型オンライン間欠的断食プログラムの減量効果に関するランダム化比較試験：パイロット研究

統計解析計画書

略称： オンライン IF 研究

研究責任者：京都大学大学院医学研究科 社会健康医学専攻
予防医療学分野 教授
石見 拓

作成者：京都大学大学院医学研究科 社会健康医学専攻
予防医療学分野 専門職学位課程
野田 貴志

統計解析責任者：京都大学大学院医学研究科 社会健康医学専攻
予防医療学分野 特定助教
西岡 典弘

2024 年 01 月 11 日 統計解析計画書 Ver.1.0
2024 年 01 月 12 日 統計解析計画書 Ver.1.1
2024 年 01 月 13 日 統計解析計画書 Ver.1.2
2024 年 01 月 15 日 統計解析計画書 Ver.2.0

【作成・変更履歴】

版番号	作成日	作成者	変更理由・概要
Ver.1.0	2024 年 01 月 11 日	野田貴志	新規作成
Ver.1.1	2024 年 1 月 12 日	野田貴志	1. 体重の欠測を多重代入法から BOCF に変更(保守的な解析、解釈と解析を単純化するため)<br>2. 副次アウトカムの身体活動量のデータ除外については、クリーニングのデザイン上生じる可能性がないため記載を削除<br>3. 解析対象集団に Completers Set(以下、「CS」)を追加。安全性解析は割合が薄まらないよう、ITT ではなく CS を対象とする。<br>4. 統計解析の実施環境が抜けていたので追記。<br>5. 最悪の補完ケースにおける解析でも、多変量解析、未調整の解析、per protocol 解析をそれぞれ実行することを明記<br>6. 身体活動及び QoL の群内差の解析手法を、対応のある t 検定から、ウィルコクソンの符号付き順位検定に変更(正規性を仮定しないため)。<br>7. メッセージの既読率と影響度については、カイ二乗検定からウィルコクソンの順位和検定に修正(%指標ではあるが「あり/なし」の2値アウトカムではないため)
Ver.1.1	1 月 13 日	野田貴志	1. Ver1.1 の変更点 5 について、最悪の補完は per protocol 解析では意味を持たないので除外(補完されるサンプルは PPS から除かれるため)<br>2. R Studo のバージョン情報を更新
Ver.2.0	1 月 15 日	野田貴志	上記変更を更新

目次

1　はじめに 1
(ア)　主要アウトカム 1
(イ)　副次アウトカム 1
(ウ)　安全性評価項目 1
2　統計的な解析 1
(ア)　中間解析 1
(イ)　目標症例数の設定根拠 1
(ウ)　用いられる有意水準 2
(エ)　臨床研究の中止基準 2
(オ)　欠測、不採用及び異常データの取り扱い 2
①　欠測データの取り扱い 2
②　不採用データの取り扱い 2
③　異常データの取り扱い 2
(カ)　当初の解析計画を変更する場合 3
(キ)　解析の対象となる臨床研究の対象者の選択 3
①　ITT 解析集団の定義 3
②　CS の定義 3
③　PPS の定義 3
3　一般的事項 4
4　統計解析項目及び方法 4
(ア)　参加者の内訳 4
(イ)　参加者の背景情報 4
(ウ)　主要アウトカム 5
(エ)　副次アウトカム 5
(オ)　安全性評価 5
①　有害事象や軽微な症状等 5
②　Quality of life（QoL） 6
5　署名 6

1 はじめに

本統計解析計画書は、研究計画書「簡易型オンライン間欠的断食プログラムの減量効果を検証するランダム化パイロット研究」Ver.2.7「5.(4) 解析の概要」についての詳細を述べたものである。

(ア) 主要アウトカム

ベースライン測定から 12 週間後の最終測定時の体重の変化量。

(イ) 副次アウトカム

① 以下の連続データの変化量。
- 体組成指標（BMI、脂肪量、筋肉量、基礎代謝量）
- 血圧指標（収縮期血圧、拡張期血圧）
- 血液指標（ヘモグロビン A1c、トリグリセリド、総コレステロール、HDL コレステロール、LDL コレステロール）
- QoL 値（SF-12v2[1]における 2 コンポーネントサマリースコア）
- 身体活動量（IPAQ short 版[2]における「総身体活動量」）

② 実行可能性の評価
- 脱落率及び遵守率
- 研究用アプリで配信した週次メッセージの既読率及び影響度

③ 探索的評価項目
- 参加者が PHR アプリ「健康日記」（以下、「研究用アプリ」）に自己記録した体重及び歩数
- 介入プログラムに対する評価や感想等

(ウ) 安全性評価項目

① 有害事象および軽微な症状の発症件数、発症例数、発症割合。

② QoL 値（2 コンポーネントサマリースコア）

2 統計的な解析

(ア) 中間解析

中間解析は実施しない。

(イ) 目標症例数の設定根拠

ターゲットサンプルサイズは 40 名(各群 20 名)とする。このパイロット研究を経て行われる将来の検証的なランダム化比較試験において想定される効果量(Cohen's d)を以下の通り 0.5 と見積もると、Whitehead らの推奨[3]では単群 10 名ずつとなる。（有意水準 0.05、検出力 0.80 の条件下）。しかし今回のパイロット研究では、オンラインによる簡易な介入で、どの程度の割合の参加者がプログラムを遵守できるかを見積もりたいという目的から、サンプルサイズはその 2 倍の、単群 20 名ずつの合計 40 名が必要だと判断した。

- 将来の検証的なランダム化比較試験において想定される効果量：

NIPPON DATA90 の統計結果[4]を参考に参加対象者（BMI≧23）の成人(20～65)の推定エ

ネルギー摂取量(必要量)を 2000kcal 程度と見積もると、今回効果を検証する介入プログラムは週に平均 1600kcal 強のエネルギー制限に相当するので、介入群の体重減少量は約 2.7kg (=1600×12÷7200)と想定される。近年日本人を対象に行われた、スマートフォンアプリによる 12 週のオンライン減量介入研究[5]の結果を参考に、対照群の体重減少量は 0.7kg、標準偏差はともに 4.0kg と想定すると効果量(Cohen's d)は 0.50 と計算される。

(ウ) 用いられる有意水準

検定の有意水準は両側 5%とする。アウトカムの推定区間は両側検定に基づく 95%信頼区間(以下、「95% CI」)とする。P 値は小数第 3 位未満を四捨五入して小数第 3 位まで表示する。ただし、小数第 3 位未満を四捨五入した値が 0.000 となる場合は必要に応じて<0.001 と表示する。副次的アウトカムの評価において、多重性の調整は行わない。

(エ) 臨床研究の中止基準

原則臨床研究は中止しないが、介入・観察との因果関係が疑われる重篤な有害事象が生じた場合や、自然災害や研究実施者の病気・死亡の場合など、研究の実施が困難だと研究者が判断した場合、臨床研究を中止することがある。

(オ) 欠測、不採用及び異常データの取り扱い

① 欠測データの取り扱い

主要アウトカムである体重については、研究デザインの性質上、ベースライン測定時の欠測は生じ得ない。脱落や追跡不能による最終測定時の欠測については、BOCF(Baseline Observation Carried Forward、以下「BOCF」)法によって補完した上で Intention to Treat(以下、「ITT」)の原則に基づき解析し、その結果を主解析の結果とする。感度分析の1つとして、介入群における最悪のケース(測定データの中で、正の方向に最大の体重変化)、対照群における最良のケース(測定データの中で、負の方向に最大の変化)で補完した解析も行う。

副次アウトカムにおいて、研究用アプリで参加者自身が自己記録した体重及び歩数については、欠測データは LOCF(Last Observation Carried Forward)法により補完し、それ以外の欠測は BOCF 法により補完する。研究用アプリで配信した週次メッセージの既読率及び影響度については、欠測データは 0 を値として補完する。

② 不採用データの取り扱い

外れ値の除外は原則行わない。外れ値の除外を行った場合は、その内容と理由を報告する。

③ 異常データの取り扱い

主要アウトカムである体重については、異常データであるかどうかの判定は行わず、データは除外しない。

副次アウトカムである身体活動量については、IPAQ ガイドライン[6]に従い、以下のように処理する。

- 各身体活動について、10 分未満の回答(とその日数)は 0 と置き換える。
- 「180 分」を超える歩行時間、中等度の身体活動時間、強い身体活動時間はデータを切り捨て、「180 分」とする。

副次アウトカムである血液指標において、計測器 cobas b 101 plus(ロシュ・ダイアグノスティックス株式会社)の測定範囲は以下のとおり[7]であり、この範囲外のデータは NA(high / low)として

結果表示される。その場合、エラー内容がhighであれば測定範囲の上限値、lowであれば測定範囲の下限値を採用する。LDL コレステロールについては NA の場合（TG 値が 400mg/dL 以上の場合）、機器で元々用いられている推定方法の通り Friedewald（CHOL−HDL−[TG÷5]）の式で計算する。

- HbA1c: 4-14%
- 総コレステロール（CHOL）: 50-500 mg/dL
- HDL-コレステール（HDL）: 15-100 mg/dL
- HDL-コレステール（LDL）：1-477 mg/dL
- トリグリセライド（TG）: 45-650 mg/dL

上記に述べた以外は、原則的に異常データであるかどうかの判定は行わず、データは除外しない。データを除外した場合は、その内容と理由を報告する。

(カ) 当初の解析計画を変更する場合

研究計画書の変更等により本統計解析計画書に変更が生じた場合は、統計解析計画書を改訂し、変更箇所・変更理由等を作成・変更履歴に記載する。

(キ) 解析の対象となる臨床研究の対象者の選択

各解析対象集団の定義を以下に示す。主要アウトカム及び副次アウトカムの解析は、ITT の原則に則り、割り付けられた全参加者を解析対象に含める。同意撤回により研究参加を取りやめる者については、撤回前のデータ（ベースライン時の測定データ）利用を許可された場合、ベースライン解析および最終解析の解析対象に含める。全データの削除を求めた参加者に関しては、ベースライン解析および最終解析から除外するが、脱落率及び遵守率の計算の際の分子及び分母には含める（4（ア）に後述）。また、Per Protocol Set (以下、「PPS」) を対象とした感度分析も実施し、解析結果の安定性を確認する。安全性に関する有害事象・軽度症状の解析については、追跡完了者 Completers Set（以下、「CS」）を対象として解析する。

① ITT 解析集団の定義

割り付けられた全集団のうち、同意撤回に伴う全データ削除を求めた参加者を除外した集団。同意撤回例のうち、ベースライン時の測定データの利用を許可した者については、解析対象に含めるものとする。追跡不能（脱落）例は、最終測定が行えないことによる欠測データを補完した上で、解析対象に含める。

② CS の定義

上記 ITT 解析集団のうち、追跡不能（脱落）例を除いた（最終測定を終えた）全集団。

③ PPS の定義

上記 CS のうち、以下に該当する例をまとめた集団。

- 介入群：最終測定時の WEB フォーム調査において、"「ファスティングの実施」について、計12 回中、指示通りにファスティングを行ったのは何回でしたか？"という質問への回答に基

づき、「完全遵守者（12 回中 12 回以上）」または「ほぼ遵守した者（（12 回中 10 回以上）」に該当する者。

- 対照群：研究用アプリで週に1通配信するメッセージの 8 割以上の既読（登録時の自動メッセージを除き、12通中10通以上）が、サーバー上のデータベースから確認できる者。

3 一般的事項

1) 要約統計量

ベースライン解析：性別は男性の数および相対度数（%）、その他の連続変数は中央値及び四分位範囲で要約する。各データは介入群と対照群について群ごとに整理する。

アウトカムの解析：体重およびその他連続変数については、変化量の平均値及び標準偏差を、小数点以下の桁数+2 桁を四捨五入して+1 桁で表示する。ただし、紙面の制約や表の整理の都合上など、適切だと判断した場合は少数第 1 位も丸めて整数表示も用いる。

2) 度数分布表

相対度数は原則、は小数第 1 位未満を四捨五入して小数第 1 位までの%表示とする。ただし、体重、BMI、ヘモグロビン A1c を除き、紙面の制約や表の整理の都合上など、適切だと判断した場合は少数第 1 位も丸めて整数表示する。

3) 信頼区間

両側 95%信頼区間とする。信頼区間の小数点以下の表示桁数は点推定値と同じ規則とする。

4) 統計解析の実施時期

全ての評価項目に係るデータの収集が完了し、データ固定後に解析を実施する。

5) 統計解析の実施環境

研究実施者である野田貴志が MacBook Pro（macOS Ventura ver.13.6.1）上のソフトウェア R（ver.4.3.2）および R studio（ver.2023.12.0+369）で解析スクリプトを設計し、テストデータにて解析が可能であることを事前に確認する。最終例のデータ取得後にデータを固定し、統計解析責任者である西岡典弘が MacBook Pro Apple M2 上のソフトウェア R（ver.4.3.2）および R studio（ver.2023.12.0+369）でスクリプトを実行してその結果を共有する。

4 統計解析項目及び方法

(ア) 参加者の内訳

CONSORT（Consolidated Standards of Reporting Trials）2010[8] に基づき、参加者の組入れ、割り付け、追跡、解析の流れをフローチャートで図示し、同意撤回例、脱落例、追跡不能例、ITT 解析対象集団、追跡完了者、PPS をその数とともに明示する。また、群ごとに脱落率（ITT解析集団のうち同意撤回及び脱落例の割合）及び遵守率（ITT 解析集団のうち PPS の割合）を求め、必要に応じてカイ二乗検定における p 値とともに報告する。また、介入群における遵守率については、2（キ）②で定義したように、「ほぼ遵守した者」および「完全遵守者」の割合もそれぞれ報告する。

(イ) 参加者の背景情報

[解析対象集団：ITT]

性別については、群ごとに男性の数および相対度数（%）を算出。連続変数である年齢、体重、BMI、身長、脂肪量、筋肉量、基礎代謝量、収縮期血圧、拡張期血圧、ヘモグロビン A1c、トリグリセ

リド、総コレステロール、HDL コレステロール、LDL コレステロール、QoL、総身体活動量については、中央値及び四分位範囲で要約する。QoL については、Qualitest 株式会社が提供する SF-12v2 日本語版の電子版システムを用い、身体的側面及び精神的側面の 2 コンポーネント・サマリースコア（1995 年日本全国調査に基づく）を要約指標として用いる。

(ウ) 主要アウトカム

［解析対象集団：ITT、PPS］

主要アウトカムである体重の変化量は、主解析として、ランダム化の際の層化因子である性別と年齢、ベースライン時の体重を共変量として用いて一般化線形回帰モデルで解析する。介入の効果について体重変化の群間差の点推定値と 95% CI、p 値を求める。各共変量の回帰係数も p 値と共に報告する。感度分析として、共変量調整なしでの変化量の比較（ウェルチの t 検定）及び、PPS における Per Protocol 解析も行う（共変量調整は主解析と同じ）。また、最終測定時の体重について欠測が生じた場合には、介入群における最悪のケースで（測定データの中で、最小の体重減少あるいは最大の体重増加に相当するように）、対照群における最良のケースで（測定データの中で、最大の体重減少あるいは最小の体重増加に相当するように）補完したデータセットにおける解析（共変量調整済みの解析、未調整での解析）も感度分析に追加する。

(エ) 副次アウトカム

［解析対象集団：ITT］

副次アウトカムのうち連続データについては、共変量調整を用いない平均群間差およびウェルチの t 検定における 95% CI を求める。生活習慣の変化を評価するための総身体活動量については、群間差だけでなく群内変化自体についても、（ウィルコクソンの符号付き順位検定）で解析する。

研究アプリで配信したメッセージの既読率（サーバーデータから確認されるメッセージの開封率）について、中央値及び四分位範囲で群ごとに要約し、（ウィルコクソンの順位和検定）における p 値を報告する。また、最終測定時の WEB フォーム調査において、“アプリで配信されたメッセージは、プログラムの遵守や生活行動に、どの程度影響を与えましたか？（0[全く影響しなかった]から 10[大いに影響した]の範囲で選択ください。）”という質問に対する答えに基づき、メッセージの影響度を 0 から 100 の%として変換し、中央値及び四分位範囲で群ごとに要約し、（ウィルコクソンの順位和検定）における p 値を報告する。

自己測定の体重及び歩数については、介入（観察）開始日を 1 日目として、12 週間後の当日（84 日目）までのデータを解析対象とし、プログラムの効果や実行可能性についての考察材料として探索的に評価する。

(オ) 安全性評価

［解析対象集団：CS］

① 有害事象や軽微な症状等

試験期間中の有害事象については、逐次報告するように求める。軽微な症状については先行研究[9]を参考に、ファスティングにおいて生じる可能性の高い「入眠のしずらさ、空腹感、疲労感、頭痛、下痢、冷え、口の渇き、腰痛、口臭、筋肉痛、腹部の膨満感、耐えられないほどの食欲、めまい、目のかすみ、脚がむずむずする、皮膚の発疹、吐き気、動悸、消化不良、筋肉の痙攣」について、最終測定時に WEB フォーム上の質問紙にて尋ねる。具体的には、“試験期間中、普段とは異なる、以下の症状を経験したことがありましたか？なければ無記入か「0」を、あればその回数を数

字で答えてください。”という質問への回答として取得する(回答欄に入力できる上限値は 90 とする)。

また、同じく最終測定時に、別の WEB フォーム上の質問紙にて、“試験期間中、研究の内容に関係があるかどうかに関わらず、健康上の有害事象(事故や怪我を含む)や体調不調・健康上のトラブル等はありましたか？”という質問によって、上記にない有害事象や症状等についての情報を取得する。このようにして取得した試験期間中の有害事象や症状等について、群ごとに発症件数、発症例数、発症割合(%)を算出する。発症割合は小数第 1 位未満を四捨五入して小数第 1 位までの%表示とするが、必要に応じて小数点以下も丸める。発症例数と発症割合については群ごとに表にまとめ、カイ二乗検定における p 値とともに報告する。

② Quality of life(QoL)

安全性評価としての QoL については、身体的側面及び精神的側面の 2 コンポーネント・サマリースコアについて、各群における変化を(ウィルコクソンの符号付き順位検定)で解析する。その他、各下位尺度得点については探索的に評価する。

5 署名

本統計解析計画書の内容が、「簡易型オンライン間欠的断食プログラムの減量効果に関するランダム化比較試験：パイロット研究」に対する統計解析計画書として妥当であること、並びに、本統計解析計画書が最終データ解析前に固定されたことを保証する。

2024年 1月 15日

[illegible] (印)

---

[1] Qualitest 株式会社. SF-12®. http://www.qualitest.jp/qol/sf12.html(2023 年 12 月 25 日 最終閲覧)

[2] Murase N, et al. Validity and reliability of Japanese version of International Physical Activity Questionnaire. Journal of Health and Welfare Statistics. 2002;49:1-9. [In Japanese]

[3] Whitehead AL, Julious SA, Cooper CL, Campbell MJ. Estimating the sample size for a pilot randomised trial to minimise the overall trial sample size for the external pilot and main trial for a continuous outcome variable. Stat Methods Med Res. 2016;25:1057-73.

[4] Yoshita K, Arai Y, Nozue M, et al. Total energy intake and intake of three major nutrients by body mass index in Japan: NIPPON DATA80 and NIPPON DATA90. J Epidemiol. 2010;20 Suppl 3:S515-23.

[5] Nakata Y, Sasai H, Gosho M, et al. A Smartphone Healthcare Application, CALO mama Plus, to Promote Weight Loss: A Randomized Controlled Trial. Nutrients. 2022;14:4608.

[6] 東京医科大学公衆衛生学分野. 国際標準化身体活動質問票のデータ処理および解析に関するガイドライン. https://www.tmu-ph.ac/news/data/180327_1.pdf (2023 年 12 月 31 日最終閲覧)

[7] ロシュ・ダイアグノスティックス株式会社. コバス b 101 プラス システムのご紹介～製品特徴と操作方法を中心に～ https://rocheacademy.jp/assets/pdf/poct/product/cobas_b_101_plus_product_introduction_material.pdf (2024 年 1 月 12 日最終閲覧)

[8] Equator network. CONSORT2010 Statement: updated guidelines for reporting parallel group randomised trials. https://www.equator-network.org/reporting-guidelines/consort (2024 年 1 月 3 日最終閲覧)

[9] Wilhelmi de Toledo F, et al. Safety, health improvement and well-being during a 4 to 21-day fasting period in an observational study including 1422 subjects. PLoS One. 2019;14:e0209353
